# A Review of Approaches to the Synthesis of DCL-Based Prostate-Specific Membrane Antigen Ligands

**DOI:** 10.3390/ijms27104642

**Published:** 2026-05-21

**Authors:** Nikolai Y. Zyk, Nina S. Butakova, Aleksei E. Machulkin, Elena K. Beloglazkina

**Affiliations:** Chemistry Department, Lomonosov Moscow State University, Leninskie Gory, Building 1/3, GSP-1, Moscow 119991, Russia

**Keywords:** prostate cancer, PSMA, peptide synthesis, PSMA ligands

## Abstract

Prostate-specific membrane antigen (PSMA) ligands are currently one of the popular platforms for creating drugs targeted at prostate cancer tumor cells. In this review, we have collected and systematized current approaches to the synthesis of PSMA ligands based on N-[N-[(*S*)-1,3-dicarboxypropyl]carbamoyl]-(*S*)-L-lysine (DCL), the most widely used scaffold in the design of such ligands. The main approaches to each stage of the synthesis of PSMA inhibitors with various structures are considered in detail, and the existing synthetic techniques are compiled and analyzed. We also review examples of using different synthetic pathways for diagnostic and therapeutic drugs based on PSMA ligands. We propose an algorithm for selecting a synthetic route based on the structure of the target ligand.

## 1. Introduction

**Figure 1:**
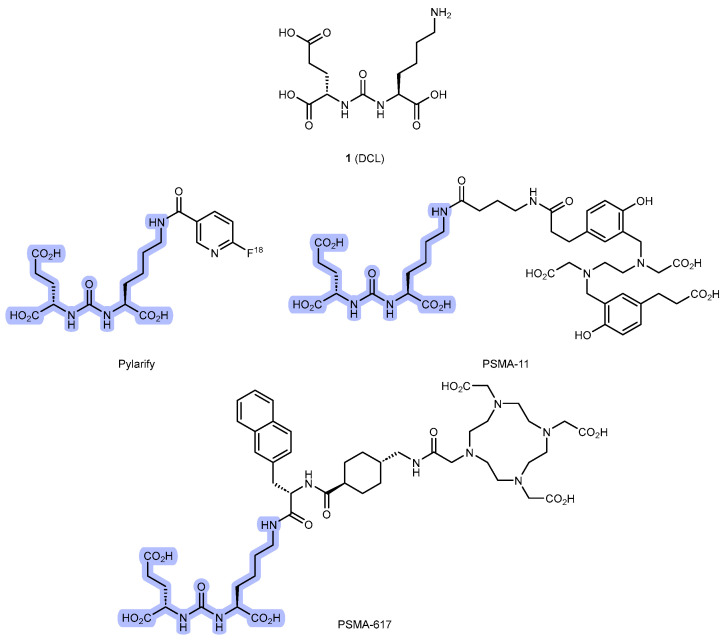
The structure of the DCL ligand and compounds based on it, approved by FDA. The DCL urea fragment is highlighted in color.

Prostate cancer (PCa) is the second most common cancer diagnosed in men [1]. Due to the serious side effects of classical treatment methods [2,3,4], recent years have seen active development of drugs capable of selectively delivering therapeutic or diagnostic payloads to tumor tissues. Compounds targeting prostate-specific membrane antigen (PSMA) have become widely used in PCa therapy, as this protein is overexpressed in tumor tissues [5,6,7,8]. The literature features a wide range of PSMA ligands of various natures and their conjugates [9,10,11,12,13,14,15,16]; however, compounds based on N-[N-[(*S*)-1,3-dicarboxypropyl]carbamoyl]-(*S*)-L-lysine (DCL, compound **1**, Figure 1) hold a special place among them. Based on the DCL ligand, the FDA-approved drugs PSMA-11, PSMA-617, and Pylarify™ were developed for PCa treatment and diagnosis [17,18,19]. Nevertheless, the search for new agents for PCa therapy and diagnostics continues, in part due to disease forms that are more resistant to treatment such as metastatic castration-resistant prostate cancer [20]. To date, several reviews have detailed the inhibitory activity of DCL-based PSMA ligands [9,10,16] and the impact of specific functional fragments on this parameter [21]. The biological activity of diagnostic and therapeutic conjugates based on these ligands has also been systematized and analyzed [11,12,13,14,15,22,23]. The aim of this work is to systematize literature approaches to the preparation of DCL urea-based PSMA inhibitors suitable for conjugation with therapeutic and diagnostic agents. Additionally, this review formulates an algorithm for selecting a synthetic route based on the target ligand’s structure. Since numerous reviews have addressed PSMA-targeted conjugates [12,13,15,24,25,26], including their synthesis methods, this aspect is not covered in depth here. However, complete synthesis schemes are provided for compounds such as PSMA-11, PSMA-617, Pylarify™, and select others.

**Figure 2:**
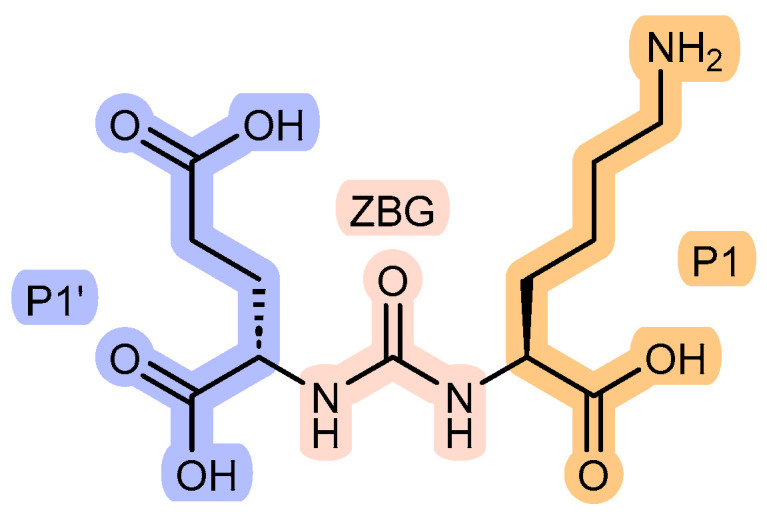
The structure of the DCL ligand with graphical isolation of fragments P1, P1′ and ZBG.

The DCL ligand is a urea derivative of glutamic acid and lysine. Based on literature data on PSMA’s structure and the binding mechanism of various ligands to this protein [9,27,28,29], DCL’s structure can be divided into three fragments (Figure 2): P1′ is the glutaric acid residue responsible for binding to the S1′ pocket; the ZBG (zinc-binding group) is the urea moiety that interacts with zinc atoms; P1 is the 6-aminohexanoic acid fragment that binds to the S1 pocket [16]. Notably, only the carboxyl group in the P1 fragment directly engages the protein, enabling diverse modifications of the ε-amino group.

Effective PSMA binding requires three carboxyl groups in the P1 and P1′ fragments [30,31]. This imposes restrictions on synthetic strategies, necessitating protecting groups (PGs) at these positions for DCL-based ligands. More complex PSMA ligands may include additional functional groups in the linker or extra fragments, which also require protection to avoid side reactions.

**Figure 3:**
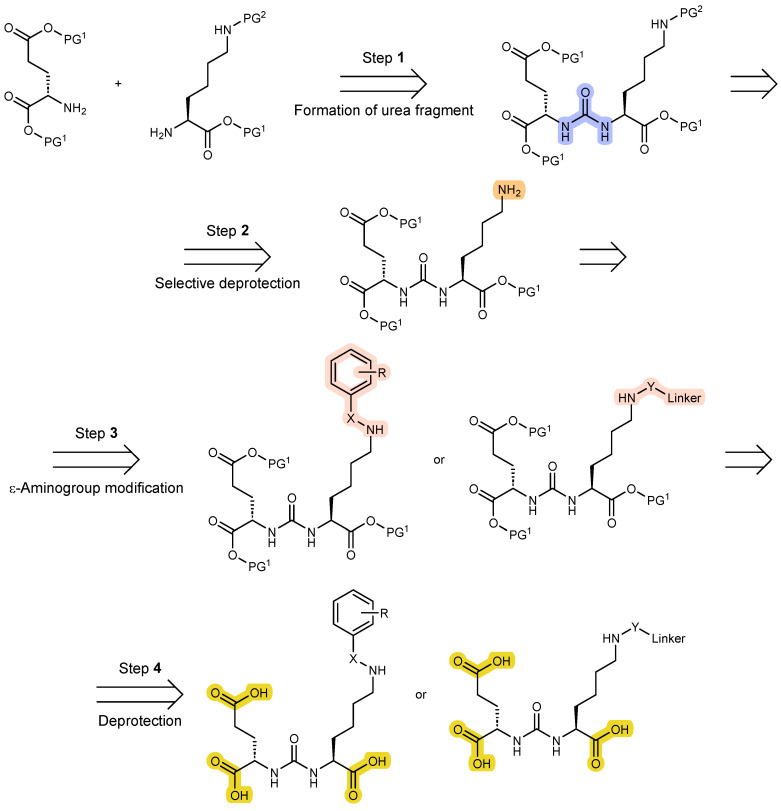
Graphical representation of the main stages of synthesis of PSMA ligands. PG^1^ and PG^2^—different protective group.

Given the above and the fact that synthetic strategy selection depends on the target PSMA inhibitor and conjugate design, four main synthesis stages can be distinguished (Figure 3):

1. Preparation of protected DCL urea, where the lysine ε-amino PG is orthogonal to those on the carboxyl groups for selective deprotection.
2. Removal of the lysine nitrogen PG to enable modification.
3. Modification of the resulting primary amine according to the target inhibitor design, potentially introducing an aromatic ε-amino fragment and/or linker.
4. Global deprotection to yield the target ligand; however, conjugation with a therapeutic or diagnostic agent may precede this in some cases.

Solid-phase synthesis strategies for PSMA ligands and their conjugates are also noteworthy. This approach differs slightly from the overall scheme outlined above and will be discussed separately in Section 4.

## 2. Methods for the Preparation of Protected DCL Urea

As noted above, the first step in PSMA ligand synthesis is the formation of the protected DCL urea. Most published syntheses use di-*tert*-butyl-protected glutamic acid and lysine protected as the *tert*-butyl ester with a Cbz group on the ε-amino group. Alternative protecting groups are also reported in the literature.

### 2.1. Synthesis of the Urea Fragment

The most common strategy employs tri-*tert*-butyl-protected DCL urea, with the lysine ε-amino group being Cbz-protected. The literature describes four main approaches: triphosgene (most widespread) [32,33,34,35,36,37,38,39,40,41,42,43,44,45,46,47,48,49,50,51,52,53,54,55,56,57,58], CDI (carbonyldiimidazole) [46,59,60,61,62,63], DSC, and *p*-nitrophenyl chloroformate (less common) [64,65,66,67,68,69].

**Scheme 1:**
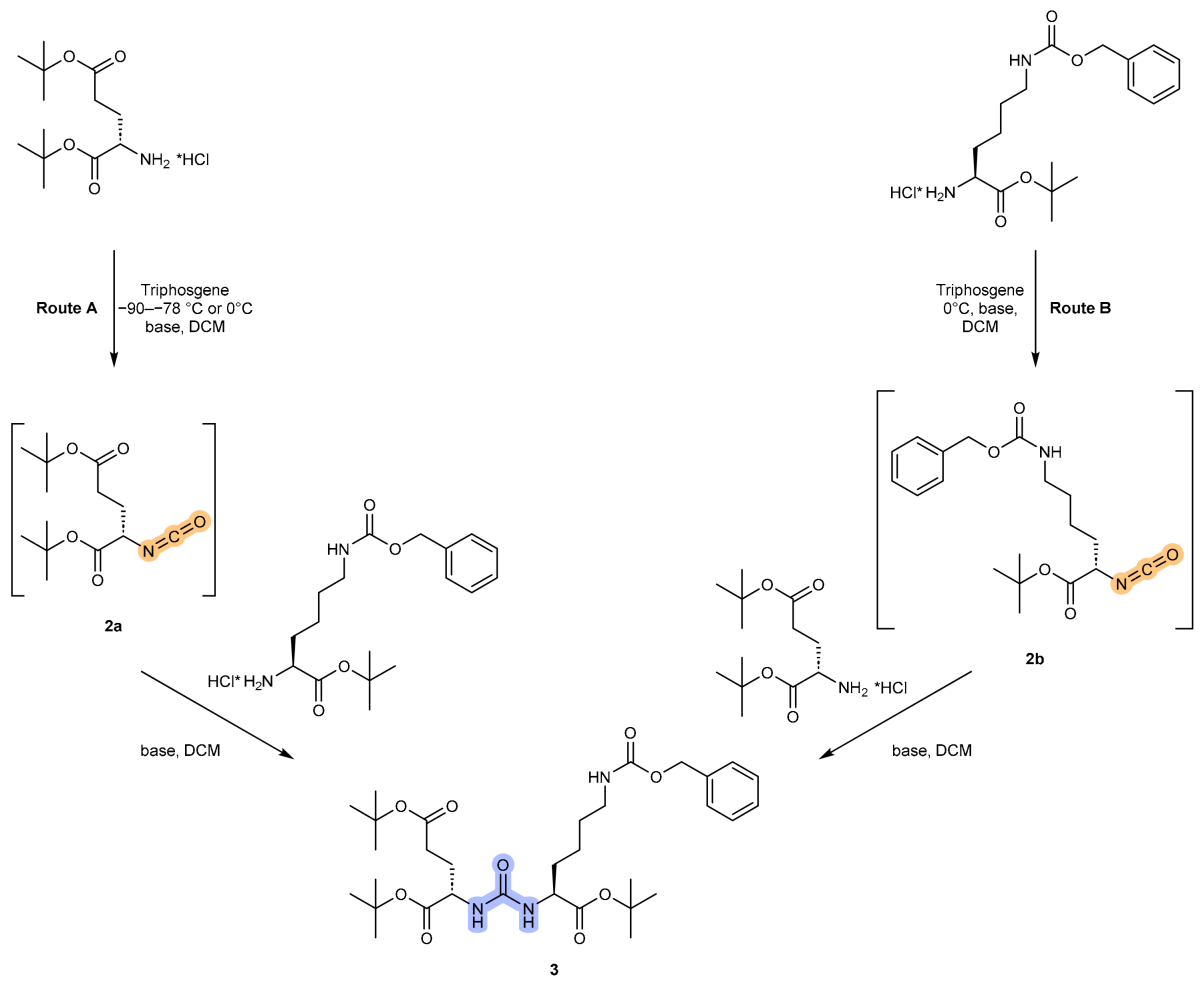
Synthesis of compound **3** using triphosgene.

The triphosgene method first involves preparation of either di-*tert*-butyl *L*-glutamate isocyanate **2a** (Route A) or N-ε-Cbz-*L*-lysine *tert*-butyl ester isocyanate **2b** (Route B), followed by reaction with the second amino acid to yield protected DCL **3** (Scheme 1).

Hydrochlorides of protected amino acids are used as starting materials, requiring excess base both to liberate the free amine and to neutralize HCl generated during isocyanate formation. DIPEA and Et_3_N are the most common bases, though pyridine, NaHCO_3_, or combinations thereof are also employed in some cases.

**Figure 4:**
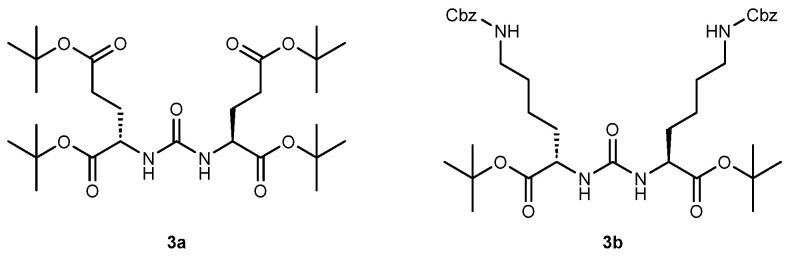
The structure of the main by-products of the urea formation reaction.

The main side reaction is formation of symmetrical ureas **3a** and **3b** (Figure 4), resulting from reaction between two molecules of protected glutamic acid or lysine, respectively.

To minimize symmetrical urea formation, two strategies are employed:

1. Slow triphosgene addition to *L*-glutamic acid di-*tert*-butyl ester at −78 to −90 °C (56–92% yield) [32,33,34,35,36,37,38,39,40,41,42,43,44,45].
2. Slow lysine addition to triphosgene at −20 to 0 °C (28–82%) [46,47,48,49,50,51,52,53,54,55,56,57,58].

It is worth noting that a more labor-intensive approach with cooling to −78 °C and below allows us to obtain the target compound **3** with a slightly higher yield (in the sources presented, the yield at this stage is 56–92%). At the same time, the preparatively simpler version with moderate cooling produces slightly lower yields (28–82%). Both described methods are easily scalable and allow synthesis of the target compound **3** in quantities from hundreds of milligrams [35,51] to tens of grams [42,58].

No clear correlation exists between yields at this stage and the base employed. For strongly cooled reactions (−78 to −90 °C), Et_3_N is most commonly used, while DIPEA is preferred for moderate cooling (−20 to 0 °C) reactions.

Notably, the lysine-isocyanate intermediate **2b** is isolated by extraction in [51], while it is generated in situ in other reports.

Compound **3** is consistently purified by column chromatography across all of the literature examples (where mentioned).

**Scheme 2:**
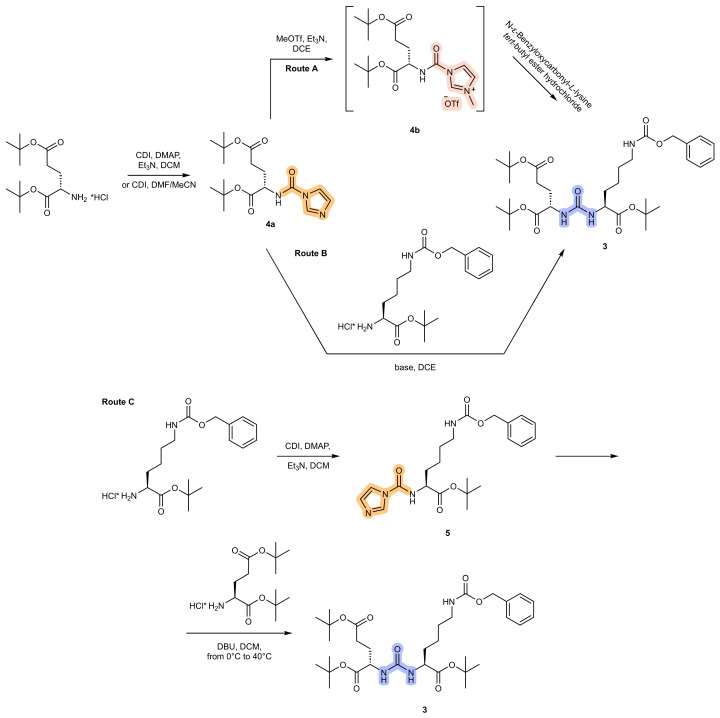
Synthesis of compound 3 using CDI.

The CDI method (Scheme 2) [46,59,60,61,62,63] is also widely used for urea **3** synthesis. In the first stage, the starting protected amino acid is reacted with CDI to produce an activated derivative **4a** (Route A and B) or **5** (Route C). After that, compound **4a** or **5** is reacted with the second amino acid to produce the target urea **3**. In a number of studies, the resulting derivative **4a** was reacted with methyl triflate (MeOTf) to produce the corresponding methylimidazolyl triflate **4b** (Scheme 2, Route A) [46,60], which in turn was reacted with the second amino acid to form target urea.

In all reports except one ([61]), the second amino acid and base were added at 0 °C, followed by heating to 40 °C. No correlation was found between yield and heating time, with compound **3** obtained in a 69–95% yield. This approach scales well, affording hundreds of milligrams [62] to tens of grams [63].

Most methods use Et_3_N/DMAP for **4a** formation in DCM. Notably, ref. [61] reports a base-free, DMAP-free protocol in DMF/ACN (1:5) at RT, achieving the highest yield (95%) among the mentioned works.

Route C (Scheme 2) is rarely used, with only one example in [63]. Here, derivative **5** reacts with di-*tert*-butyl *L*-glutamate using DBU as a base, affording compound **3** in 80% yield, so it does not give any significant improvement over standard routes.

**Scheme 3:**
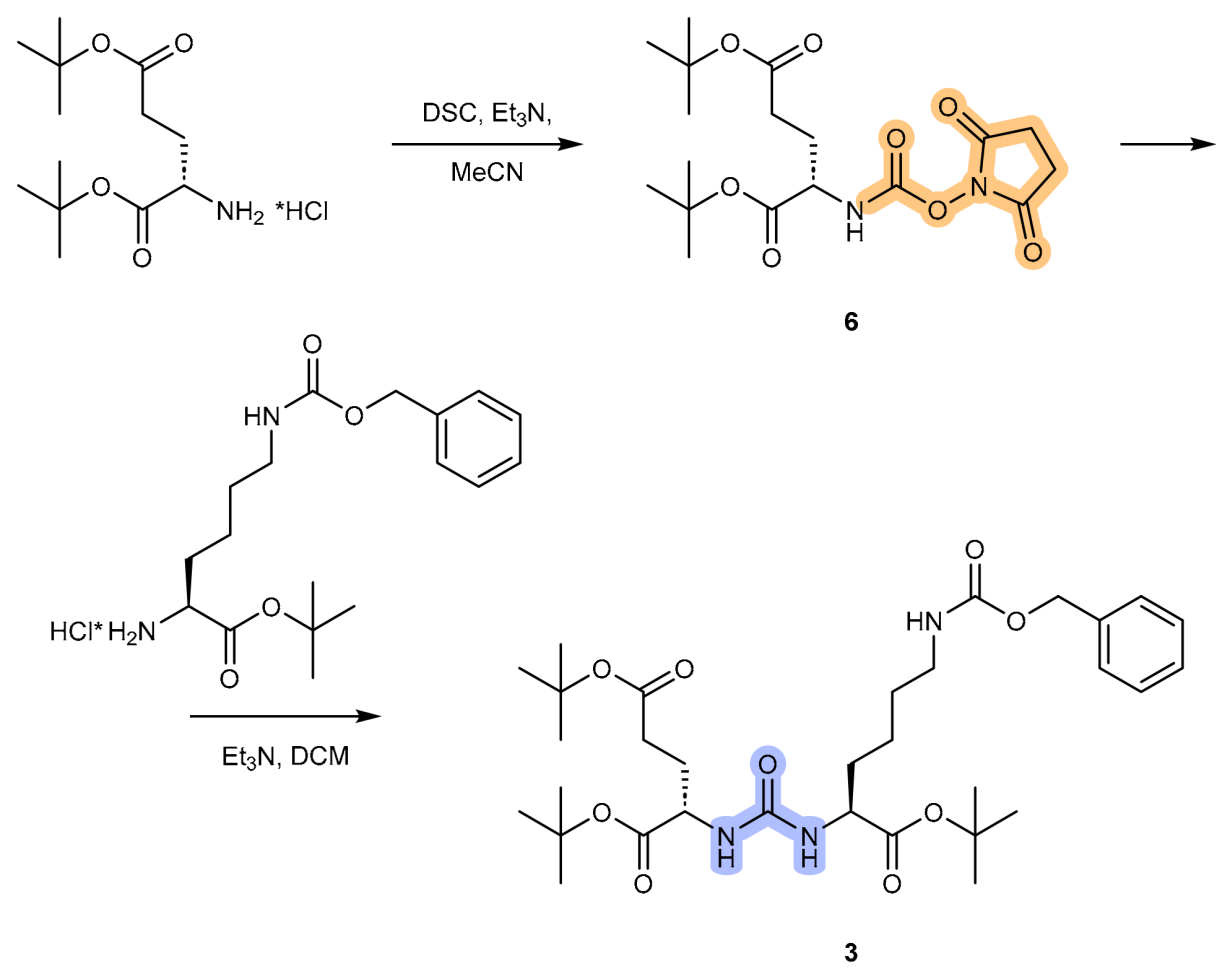
Synthesis of protected urea **3** using DSC.

Another approach used for the synthesis of compound **3** involves reaction with disuccinimidyl carbonate (Scheme 3) [64,65,66].

The DSC method (Scheme 3) involves reaction of protected glutamic acid with disuccinimidyl carbonate to form compound **6**, which is isolated and then reacted with protected lysine in the presence of triethylamine in DCM. Yields range from 53 to 76%. The available literature data is insufficient to assess the scalability of this approach.

**Scheme 4:**
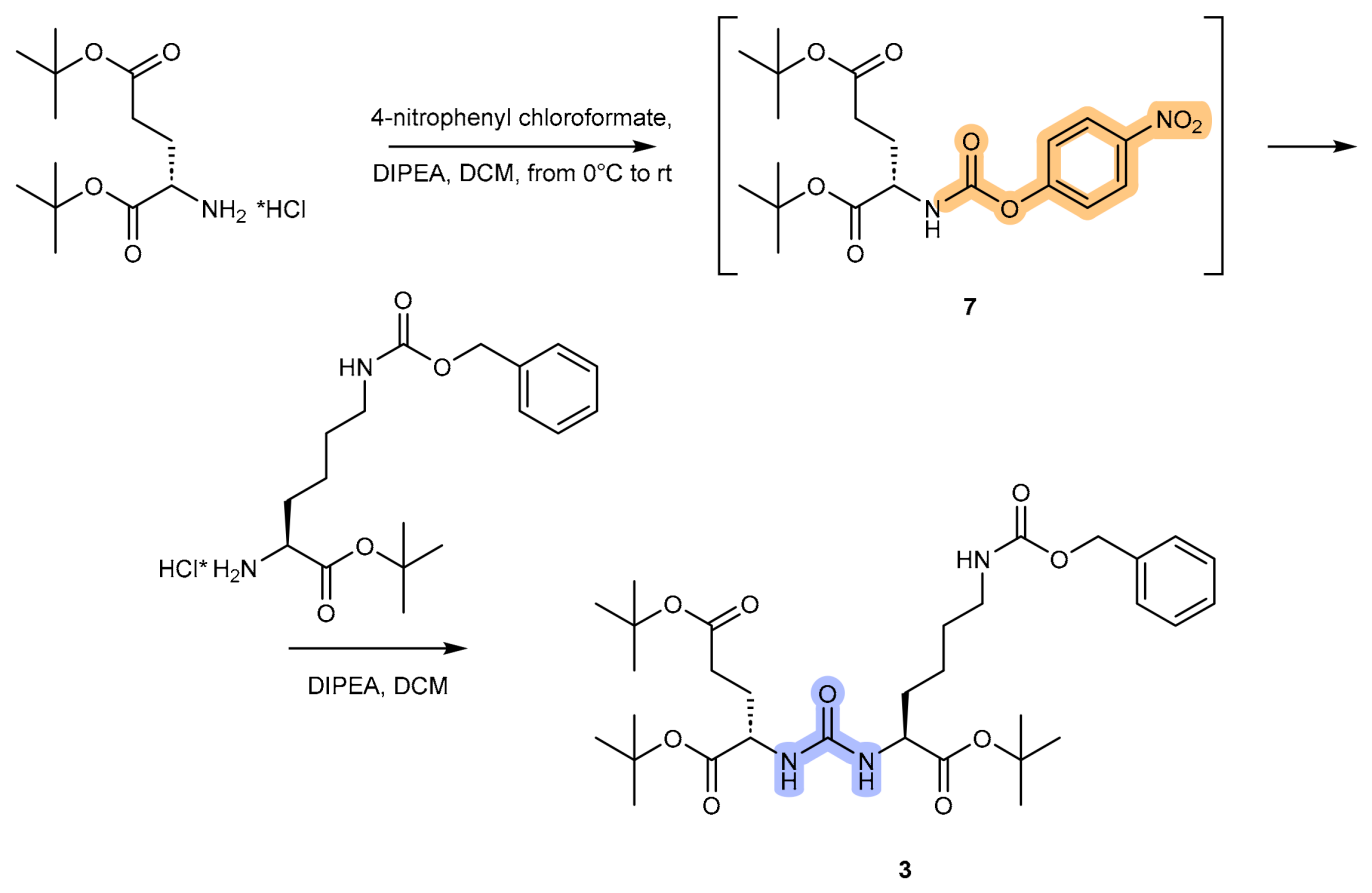
Preparation of compound **3** using 4-nitrophenyl chloroformate.

Another approach for compound **3** synthesis uses *p*-nitrophenyl chloroformate [67,68,69]. Protected glutamic acid reacts with *p*-nitrophenyl chloroformate to form carbamate **7** in situ (Scheme 4), which then reacts with protected lysine. This method is adapted from earlier G2 carboxypeptidase inhibitor syntheses [70].

This method affords protected DCL urea in high yields (86–90%). However, the available literature data is insufficient to assess scalability.

**Scheme 5:**
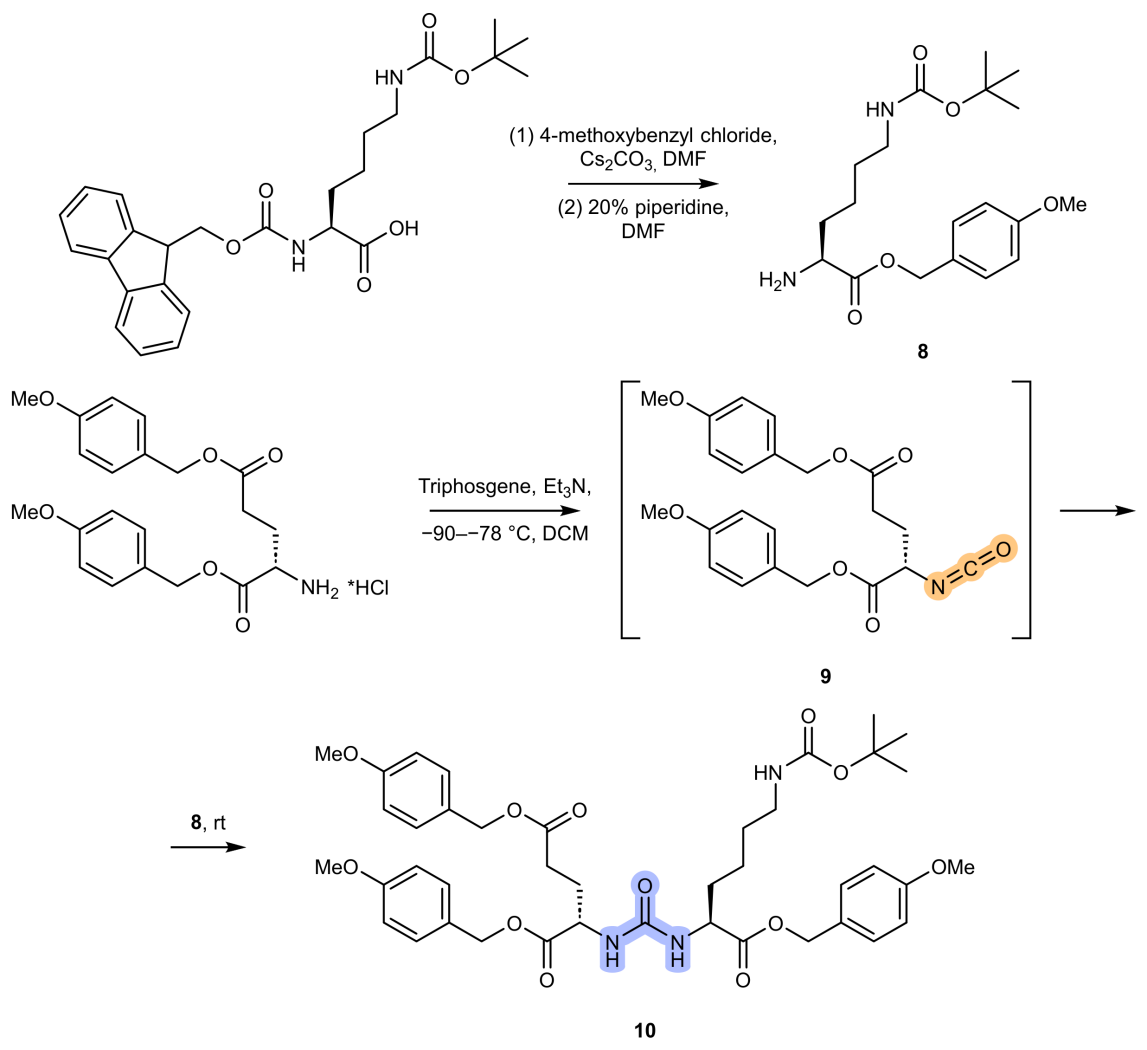
Scheme for obtaining PMB-protected form of DCL **10**.

Beyond the patent literature, a single non-*t*Bu approach employs *para*-methoxybenzyl (PMB) esters for all three carboxyl groups and Boc protection for the lysine ε-amino group [71,72,73,74]. The route begins with Nα-Fmoc-Nε-Boc-*L*-lysine, which is converted to the PMB-ester followed by selective Fmoc deprotection to furnish free amine **8** (Scheme 5). Separately, *L*-glutamic acid bis-PMB ester hydrochloride is reacted with triphosgene under standard conditions (Scheme 1, Route A) (−78 °C, Et_3_N) to generate isocyanate **9** in situ. Addition of amine **8** to compound **9** yields the fully protected DCL urea **10** in 62% yield for the final step. Overall yield over three steps: 48% [71].

Comparative data on methods for preparing protected DCL ureas (compounds **3** and **10**) are summarized in Table 1.

Table 1 highlights methods affording compound **3** with the highest yields: triphosgene with strong cooling, CDI activation, and *p*-nitrophenyl chloroformate. The preparatively simplest DSC approach, requiring neither cooling nor heating, provides yields ≤ 76%. All methods enable synthesis of protected DCL urea on the scale of grams to tens of grams; however, the limited number of DSC and *p*-nitrophenyl chloroformate examples precludes definitive assessment of their scalability.

For compound **10**, only the protocol with triphosgene/strong cooling is reported. The employed starting materials are, however, compatible with alternative methods developed for compound **3** synthesis.

### 2.2. Removal of the Protective Group at the ε-Amino Group of Lysine

ε-Amino group deprotection of compound **3** involves selective removal of the lysine Cbz group, as reported in the majority of literature examples. Boc protection is occasionally employed, with deprotection conditions discussed separately.

**Scheme 6:**
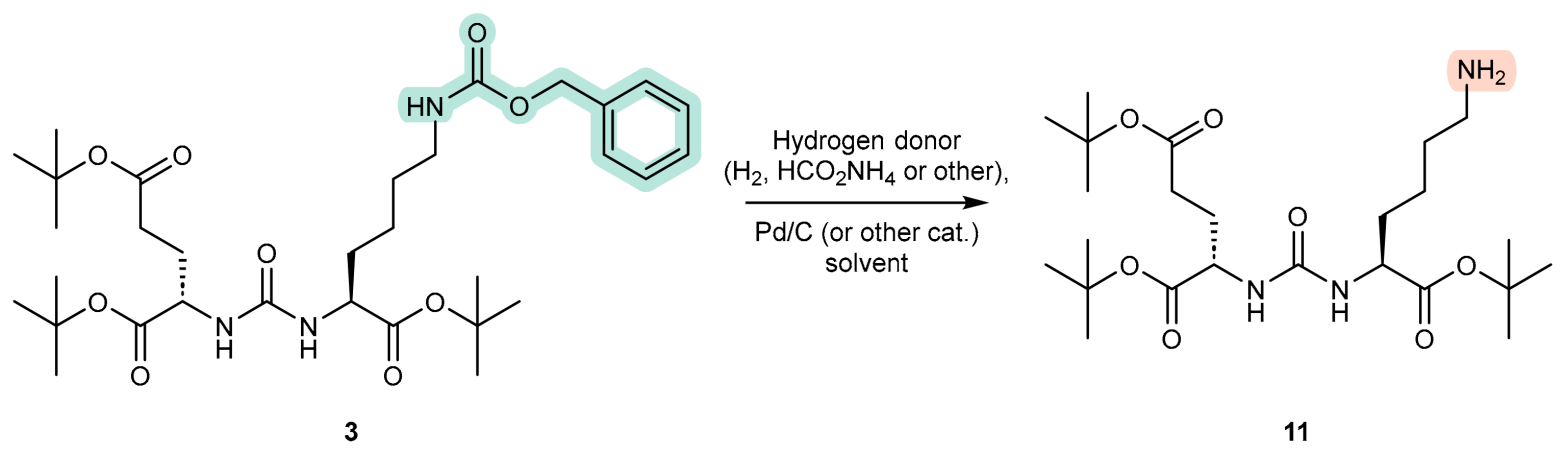
General scheme for obtaining compound **11**.

Since acid-labile *t*Bu esters protect the carboxyl groups, acid-mediated Cbz cleavage (hydrochloric acid [75,76], hydrobromic acid [75], trifluoromethanesulfonic acid [77] or methanesulfonic acid [78]) is precluded. To obtain compound **11**, the main approach is hydrogenolysis (Scheme 6). Methods using hydrogen and palladium on charcoal as a catalyst have become the most widespread [32,33,34,35,36,37,39,40,41,43,44,49,50,51,54,55,57,58,61,63,64,66,68,79]. An approach using ammonium formate as a hydrogen donor is also often used [42,45,46,47,48,53,56,60,80,81,82]. In addition to these two most common methods, there are examples of the use of 1,4-cyclohexadiene and hydrazine hydrate [52,65]. Yields typically exceed 90% [43,46]; however, some reports note yields <70% [36,47].

Among methods employing molecular hydrogen, 10% palladium on charcoal is the most frequently utilized catalyst across the literature. One example describes the use of 5% Pd/C [63]; however, the corresponding yield of deprotected amine **11** was not disclosed. Palladium hydroxide on carbon (Pd(OH)_2_/C) serves as an effective alternative [51], yielding compound **11** in an excellent 99% yield under standard hydrogenolysis conditions.

The reaction is conducted in different solvents including methanol, methanol/water mixtures, ethanol, ethanol/toluene, or ethyl acetate. While solvent choice varies widely across reports, no clear correlation between the solvent system and the isolated yield of compound **11** could be established from the available data. Methanol remains the most commonly employed solvent.

In cases employing ammonium formate as a hydrogen donor, 10% Pd/C catalysis in ethanol is standard across literature reports.

Hydrogenolysis with 1,4-cyclohexadiene requires an inert atmosphere (N_2_ or Ar) with Pd/C as a catalyst [65]. Compound **11** was isolated with 85% yield.

The hydrazine hydrate method also involves the use of Pd/C and makes it possible to obtain the target compound **11** with a yield of 89% [52].

In the vast majority of examples, catalyst removal is achieved by filtration through Celite (Kieselguhr), effectively eliminating Pd/C residues. Additional column chromatography for compound **11** purification was typically unnecessary, streamlining the process.

In the overwhelming majority of cases, filtration was used to purify the product in order to get rid of catalyst residues. However, in many examples, additional purification of compound **11** using column chromatography was not performed.

Selective removal of the Cbz group from the lysine ε-amino position represents a well-optimized, thoroughly documented process in the literature. All four described approaches afford compound **11** in yields exceeding 80%. The predominant H_2_ and ammonium formate methods exhibit excellent scalability, enabling production from hundreds of milligrams to tens of grams. While molecular hydrogen offers robust reactivity, it requires specialized equipment and enhanced safety precautions. Ammonium formate provides a safer, more convenient alternative that eliminates hydrogen gas handling while maintaining comparable efficiency. Although 1,4-cyclohexadiene and hydrazine hydrate methods offer operational simplicity versus H_2_, their yields do not surpass ammonium formate systems, explaining their limited adoption.

**Scheme 7:**
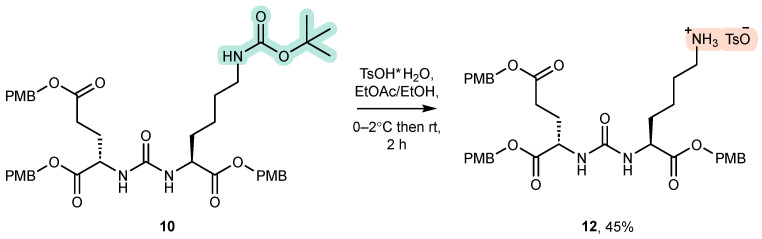
Removal of the Boc protective group to obtain compound **12**.

For compound **10**, selective removal of the Boc group from the lysine ε-amino position follows Scheme 7 [71,72,73,74]. Both Boc and PMB protecting groups are acid-labile. Under the authors’ Boc-deprotection conditions, the yield was only 45% [71], which is significantly lower than the >80% yields typically observed for Cbz-protected systems.

The reported Boc deprotection protocol involved dissolving compound **10** in ethyl acetate, cooling to 0–2 °C, and adding a solution of *p*-toluenesulfonic acid monohydrate (*p*-TsOH·H_2_O) in ethanol. Stirring continued at room temperature for 2 h, after which product **12** was isolated as the tosylate salt by column chromatography.

## 3. Strategies for Modification of the ε-Amino Group of the Lysine Fragment

Two major classes of ε-amino modification of the lysine fragment can be distinguished.

The first class involves introduction of an aromatic fragment at this position. This modification is motivated by the presence of a hydrophobic subpocket adjacent to the S1 pocket, which can interact with aromatic fragments and thereby enhance ligand binding to PSMA [83]. Based on this, such fragments are often introduced into the ligand structure to increase affinity for PSMA. At the same time, these aromatic fragments can serve as sites for subsequent functionalization with halogen radioisotopes (^125^I, ^211^At, or ^18^F), provided they contain an appropriate leaving group (e.g., Sn(*n*-Bu)_3_ or NMe_3_^+^).

The second major class of lysine ε-amino modifications involves incorporation of a linker into the ligand structure. This serves to distance the therapeutic or diagnostic moiety in the final conjugate from the urea fragment responsible for PSMA active site binding. Additionally, linker side chains can engage hydrophobic pockets in the access tunnel to the active site, further enhancing binding affinity [84]. For the last purpose, amino acid fragments containing aromatic fragments (e.g., phenylalanine and tyrosine) can be introduced into the linker. Also, the structure of the linker has a significant effect on the physical, physico-chemical and pharmacokinetic properties of the final PSMA inhibitors and conjugates based on them. In this regard, in some cases linkers have a rather complex structure.

Some established PSMA ligands incorporate both an aromatic fragment at the ε-amino position and a linker, which influences their synthetic strategies.

### 3.1. Existing Approaches to Modification of the ε-Amino Group of Lysine by Aromatic Fragments

**Scheme 8:**
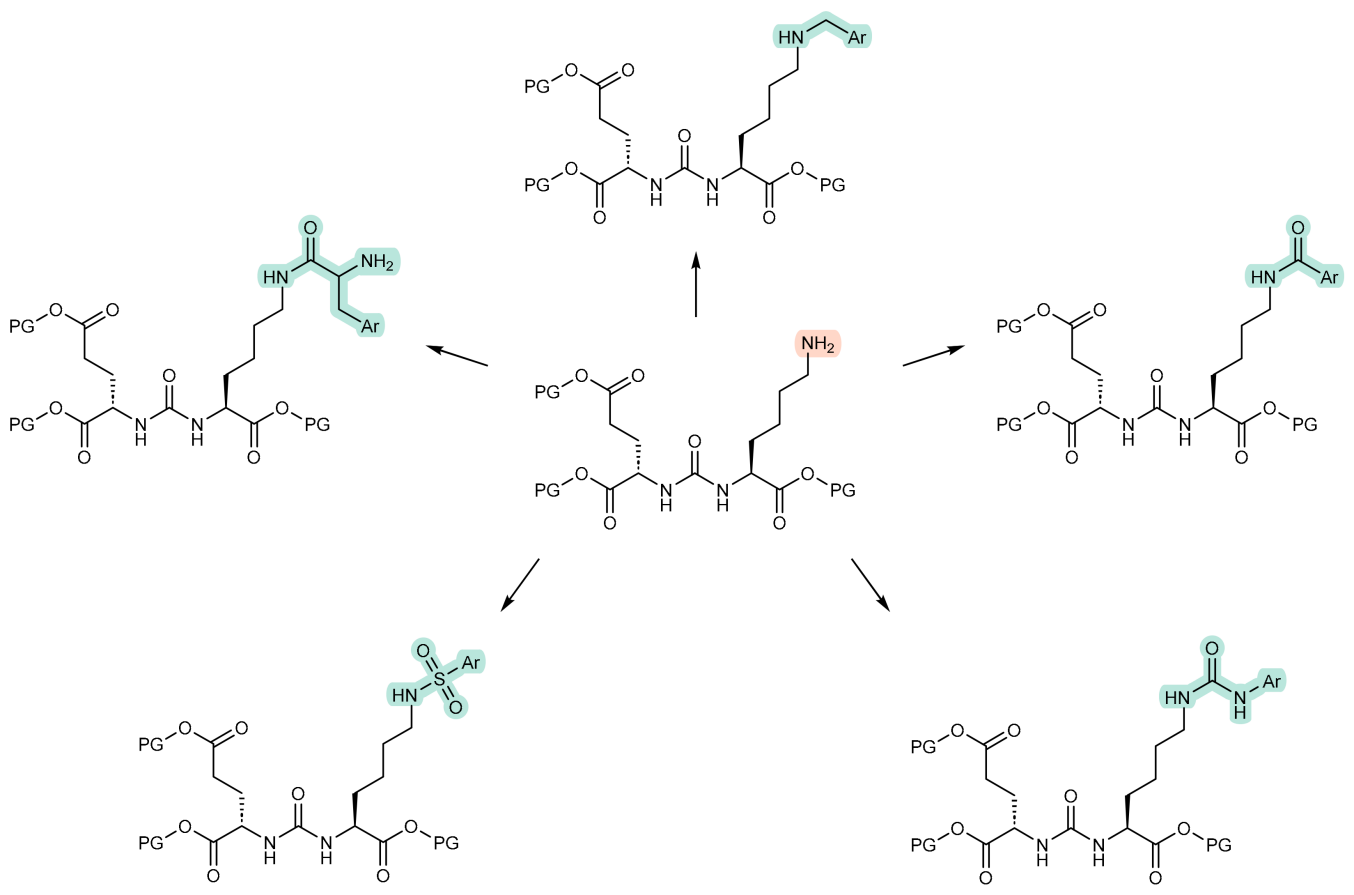
General scheme of possible modifications of the ε-amino group of protected urea DCL by aromatic fragments.

Five principal approaches to modification of the DCL urea ε-amino group with aryl-containing substituents can be distinguished (Scheme 8). The first involves N-alkylation via reductive amination with the corresponding benzaldehyde. The second employs acylation of the amino group with aromatic carboxylic acids or their derivatives. The third and fourth modification pathways involve formation of a urea or sulfamide bridge, respectively, between the aromatic fragment and the lysine residue. The fifth pathway also proceeds through acylation of the ε-amino group but utilizes amino acids bearing aromatic side chains (e.g., *L*-naphthylalanine in PSMA-617, Figure 1).

**Scheme 9:**
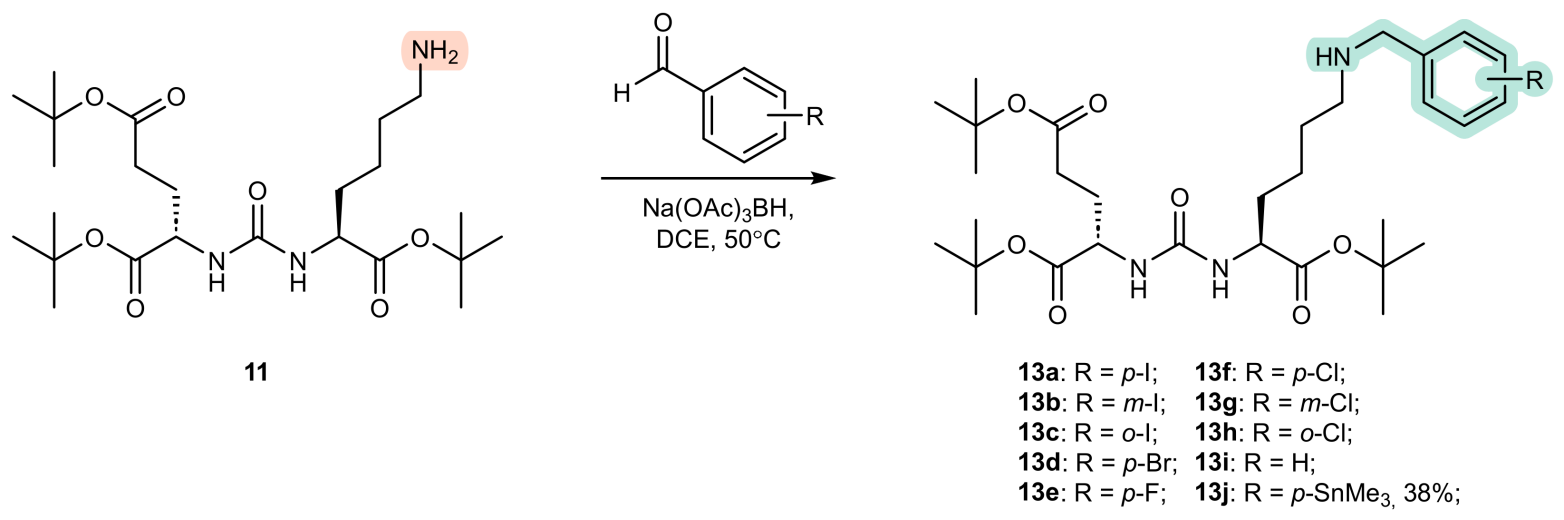
Obtainment of derivatives **13a**–**j**.

The alkylation of the ε-amino group of lysine via reductive amination makes it possible to further modify the nitrogen atom by introducing a linker into the ligand structure and is widely described in the literature. In this manner, **13a**–**j** derivatives were synthesized in [46] (Scheme 9).

The reaction between aldehyde and urea **11** was carried out in dichloroethane, and sodium triacetoxyborohydride was used as a reducing agent. Notably, only for compound **13j**—bearing a *para*-trimethylstannyl group—was the yield of this stage explicitly reported (38%).

The technique using STAB was also applied in [37] to obtain compounds **13d**–**f**. Also, a similar compound with a hydroxyl group in the *para*-position was synthesized. The yield of the target compounds ranged from 40% to 74%.

The synthesis of compound **13d** using sodium cyanoborohydride as a reducing agent was described in [85,86]. The reaction was carried out in methanol with the addition of acetic acid, and the yield was 48%.

Reductive amination can be performed using sodium borohydride. Thus, compounds **13d**, **13f**–**g**, and **13i** were synthesized using NaBH_4_ in [43,87] (Scheme 9). To prevent the side reaction of benzaldehyde reduction to benzyl alcohol, NaBH_4_ was added portionwise. This makes the procedure somewhat more labor-intensive than approaches using STAB or NaBH_3_CN. Under the proposed conditions, the yield of these compounds was 68–90%. At the same time, new compounds containing halogen atoms in the benzyl fragment or functional groups of a different nature (carboxy-, nitro-, alkoxy- and others) were synthesized. The reaction yield for the new ligands ranged from 30 to 88%.

**Scheme 10:**
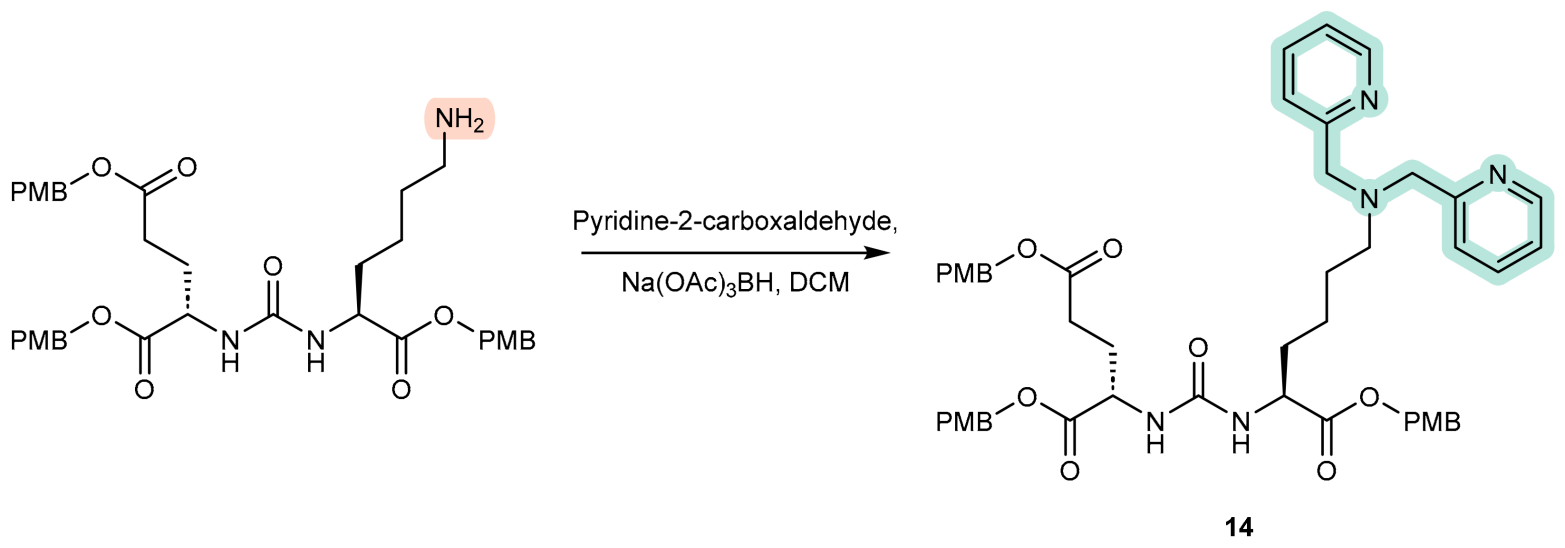
Synthesis of compound **14**.

In addition to substituted benzyl fragments, heterocyclic structural motifs can also be introduced into the ligand structure by this method. In [71], compound **14** was obtained using reductive amination (Scheme 10). The reaction was carried out under the conditions to obtain a dialkylated product. Two pyridine fragments were necessary to subsequently obtain the complexes with rhenium and technetium based on compound **14**.

**Scheme 11:**
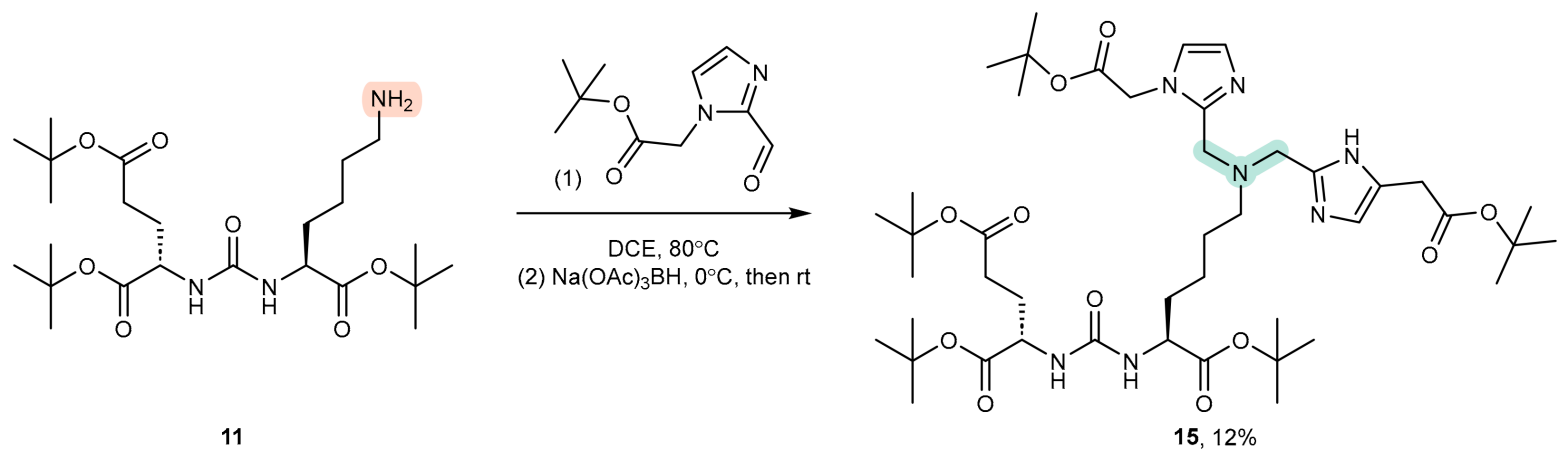
Synthesis of compound **15**.

Using a similar technique, compound **15** containing two fragments of N-substituted imidazole was synthesized [88]. However, a target compound was obtained only with a low yield (12%) (Scheme 11).

Later, in Section 3.2, some approaches to the modification of such alkylated derivatives of DCL urea will be considered.

The second approach involves acylation of the lysine ε-amino group with aryl- and hetaryl-containing acids. Various carboxylic acid derivatives (NHS- and TFP-esters) or coupling agents (HBTU, HATU, etc.) are employed for this transformation.

**Scheme 12:**
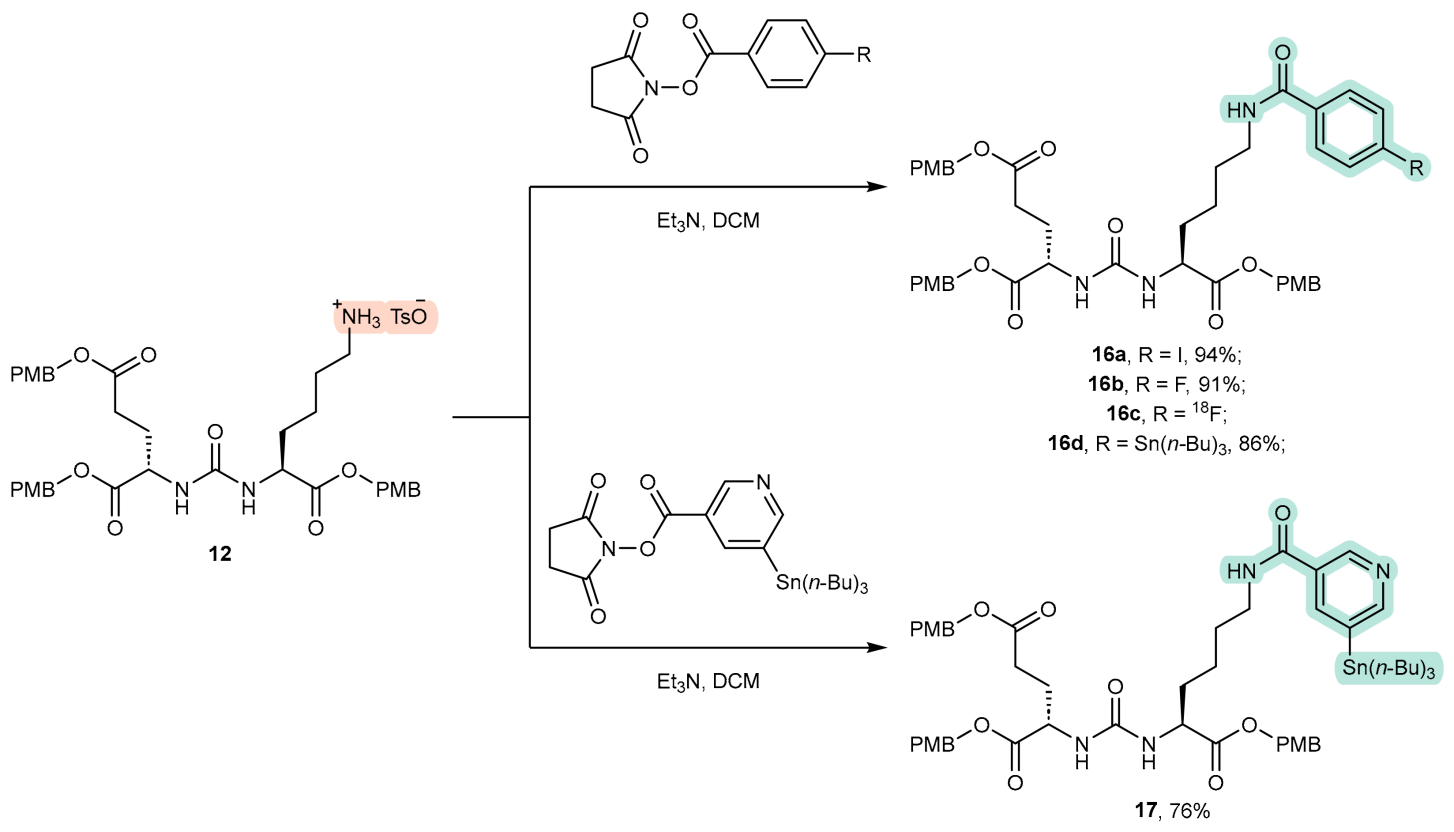
Synthesis of derivatives **16a**–**d** and **17**.

This approach often lets us introduce fragments suitable for creating compounds labeled ^18^F or ^125^I on their basis. Thus, in [72], the authors synthesized compounds **16a**–**d** and **17** by acylation of compound **12** with the appropriate NHS-esters (Scheme 12). Compounds **16a**,**b** contained non-radioactive isotopes of iodine and fluorine and were synthesized to study the inhibitory activity of compounds of such a structure. Compound **16c** was a protected derivative labeled ^18^F. Compound **16d** contained a tri-*n*-butylstannyl group in its structure, which was subsequently necessary for labeling with the isotope ^125^I. Compound **17** also had a tin-containing fragment; however, unlike the series of compounds **16**, it was a derivative of nicotinic acid.

Since compound **12** was employed as the tosylate salt, acylation reactions required triethylamine as a base. In all reported cases the acylations proceeded with fairly high yields. The exception was compound 16c: it was carried forward to the next stage without additional purification, and no yield was reported for this step. It is also worth noting that these reactions proceed quite quickly—from 30 min to 2 h. Moreover, except for compound **16c**, they were carried out without heating.

**Scheme 13:**
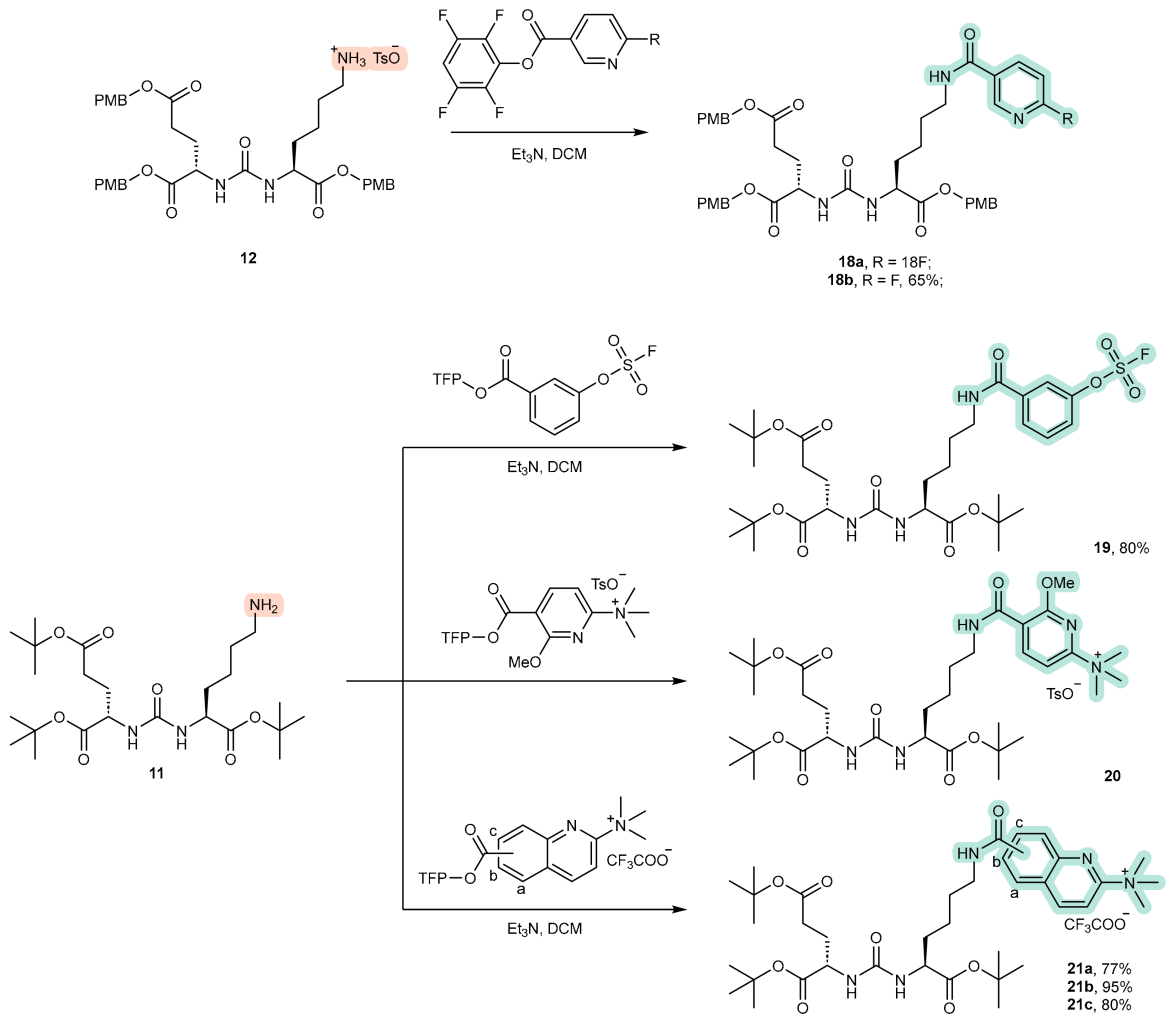
Approaches to DCL modification using TFP-esters.

TFP-esters can also be used instead of NHS-esters. This variant of acylation is somewhat more represented in the works dedicated to the modification of DCL. For example, a similar approach was used in the synthesis of the Pylarify drug (compound **18a**, Scheme 13) [74]. In the case of compound **18a**, the authors did not provide a yield, as they did not isolate it individually. In the case of an analog with a natural isotope of fluorine **18b**, the yield of the acylation reaction was 65%. The PMB-protected form of DCL **12** was used as the initial compound.

Similar conditions were applied to obtain compound **19**, which contains an (fluorosulfonyl)oxy group [89]. Also, the authors obtained dimeric derivatives containing two carboxyl groups in meta-positions to the SO_3_F group. In similar conditions, they also used TFP-ester and the product was isolated with 75% yield.

TFP esters can alternatively be used to introduce aromatic fragments into a structure that contains a trimethylammonium group in the ring; this group is subsequently replaced by ^18^F (compounds **20** and **21a**–**c**) [90,91]. Article [91] did not provide synthetic details of the preparation of compound **20**. In [90], derivatives **21a**–**c** was obtained with 77–95% yields. The reactions were carried out at room temperature for 2 h, and the isolation did not include column chromatography, which makes the proposed method quite simple from a preparative point of view.

**Scheme 14:**
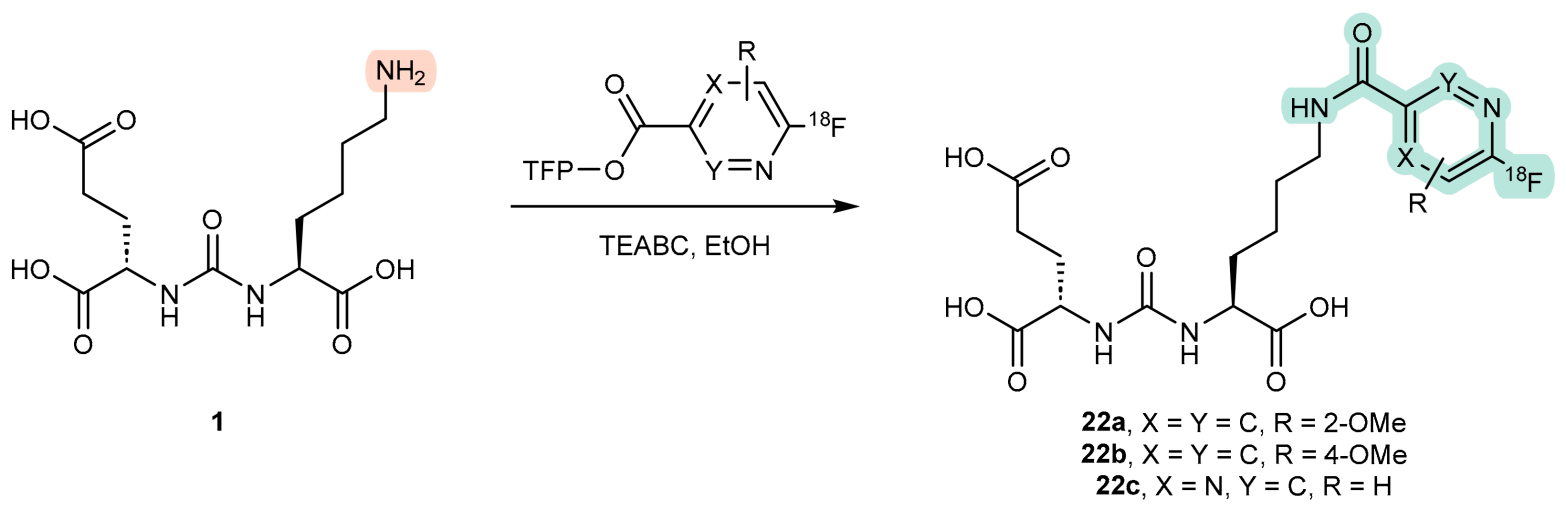
Synthesis of radiolabeled **22a**–**c** derivatives based on DCL urea.

It should be mentioned that TFP-esters can be reacted directly with DCL urea **1**, which does not contain protective groups. In [92], the authors obtained in this manner a series of derivatives **22a**–**c** (Scheme 14) with the ^18^F isotope.

The authors note that the acylation stage was possible only in anhydrous ethanol. In aprotic or aqueous conditions, there was a significant decrease in radiochemical conversion (0–15%). Subsequent purification of the target compounds using solid-phase extraction or high-performance liquid chromatography allowed to obtain products with reasonable radiochemical yields (12–25%, non–decay-corrected) and radiochemical purity (>98%).

**Scheme 15:**
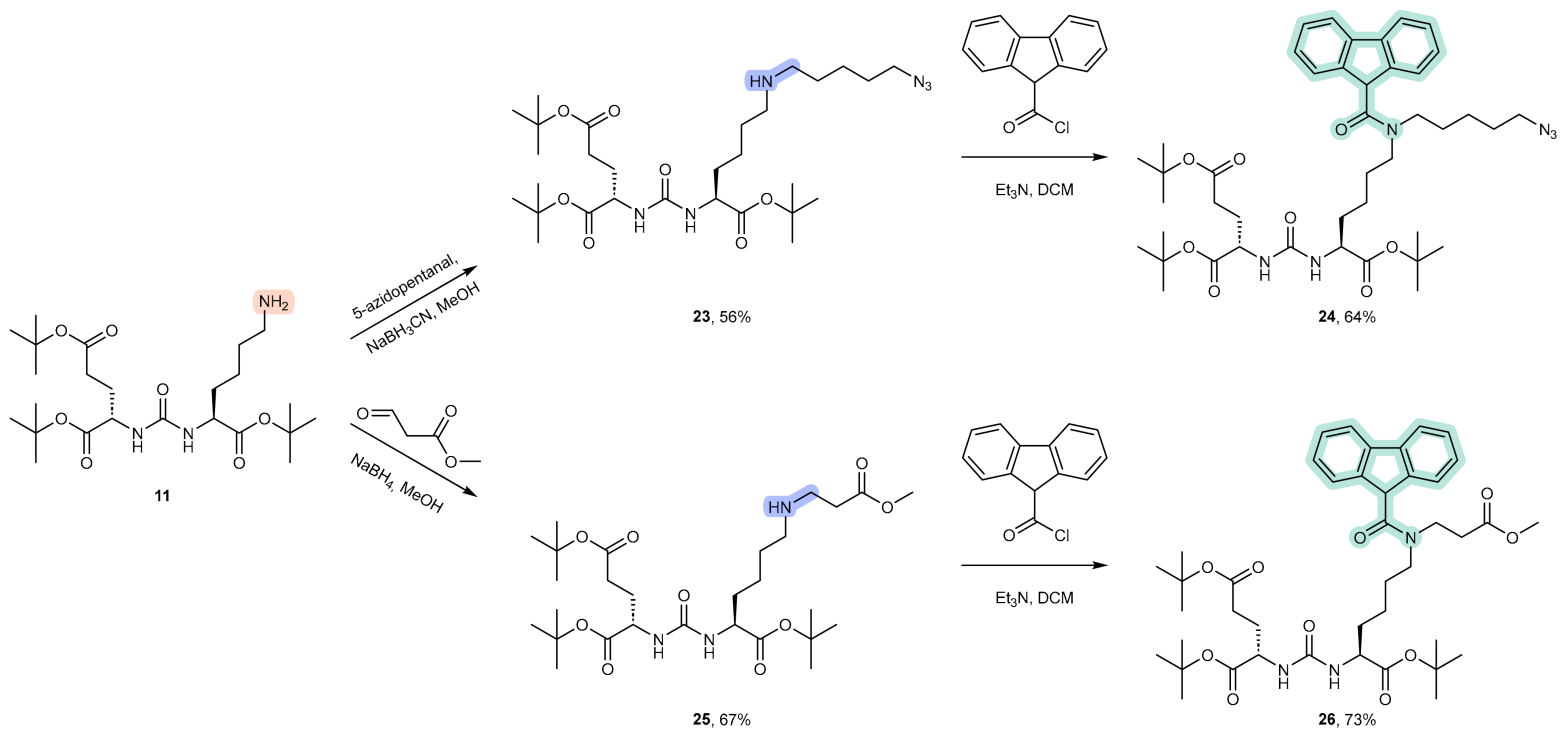
The introduction of a 9*H*-carboxy-9-fluorene residue into the structure of the PSMA ligand due to the acylation reaction.

Acyl chlorides can also be employed for acylation of the ε-amino group of lysine, as demonstrated in [93,94] on the example of 9*H*-carboxyfluorene (Scheme 15). A distinctive feature of these studies is that the aromatic fragment was introduced after the amino group of lysine was modified by the linker fragment. The introduction of the linker was carried using reductive amination reaction. In [93], 5-azidopentanal was used as a fragment of the linker, producing secondary amine **23** (yield 56%). This amine was reacted with 9*H*-fluorene-9-carbonyl chloride to obtain compound **24** with 64% yield. Further approaches to linker modification will be discussed in Section 3.2.

In [94], the authors carried out the reductive amination reaction of compound **11** and the methyl ester of 3-oxopropanoic acid to obtain amine **25** (yield 67%), which was then acylated with 9*H*-fluorene-9-carbonyl chloride. Thus, compound **26** was obtained with a 73% yield.

**Scheme 16:**
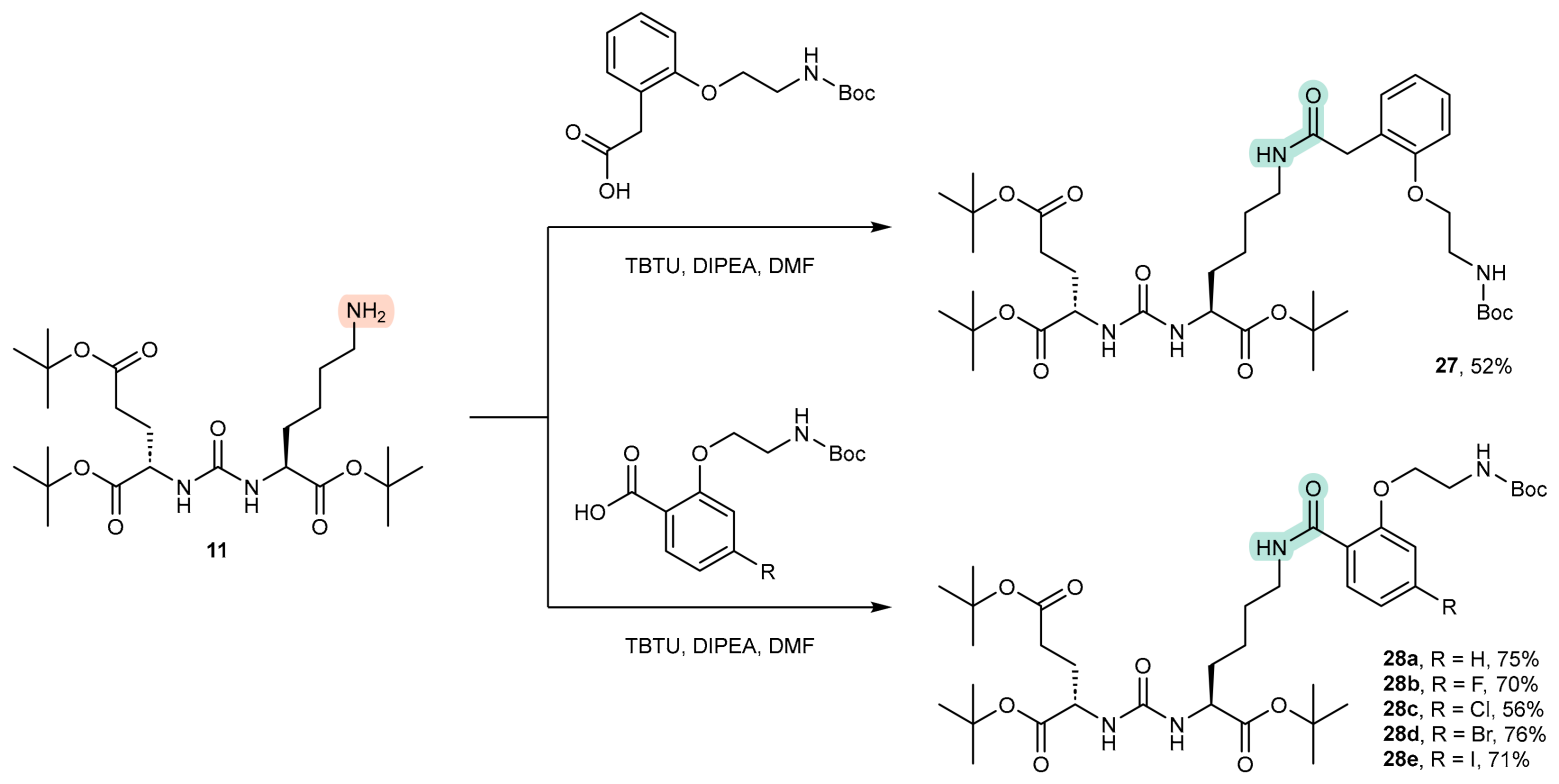
Variants of modification of protected urea DCL, presented in [85].

The presence of *tert*-butyl substituents for the carboxyl groups of urea **11** also allows acylation using various amide coupling agents (TBTU, HATU and others). In [85], the authors proposed a method for the acylation of urea **11** using TBTU as an activator (Scheme 16).

In the case of substituted phenylacetic acid, protected DCL **11** was introduced into the reaction and the proposed reaction conditions made it possible to obtain product **27** with 52% yield. At the same time, the acylation of urea **11** with different disubstituted benzoic acids made it possible to obtain compounds **28a**–**e** with higher yields (≥70% for all examples except **28c**). In all cases, the reaction was carried out in the presence of DIPEA.

**Scheme 17:**
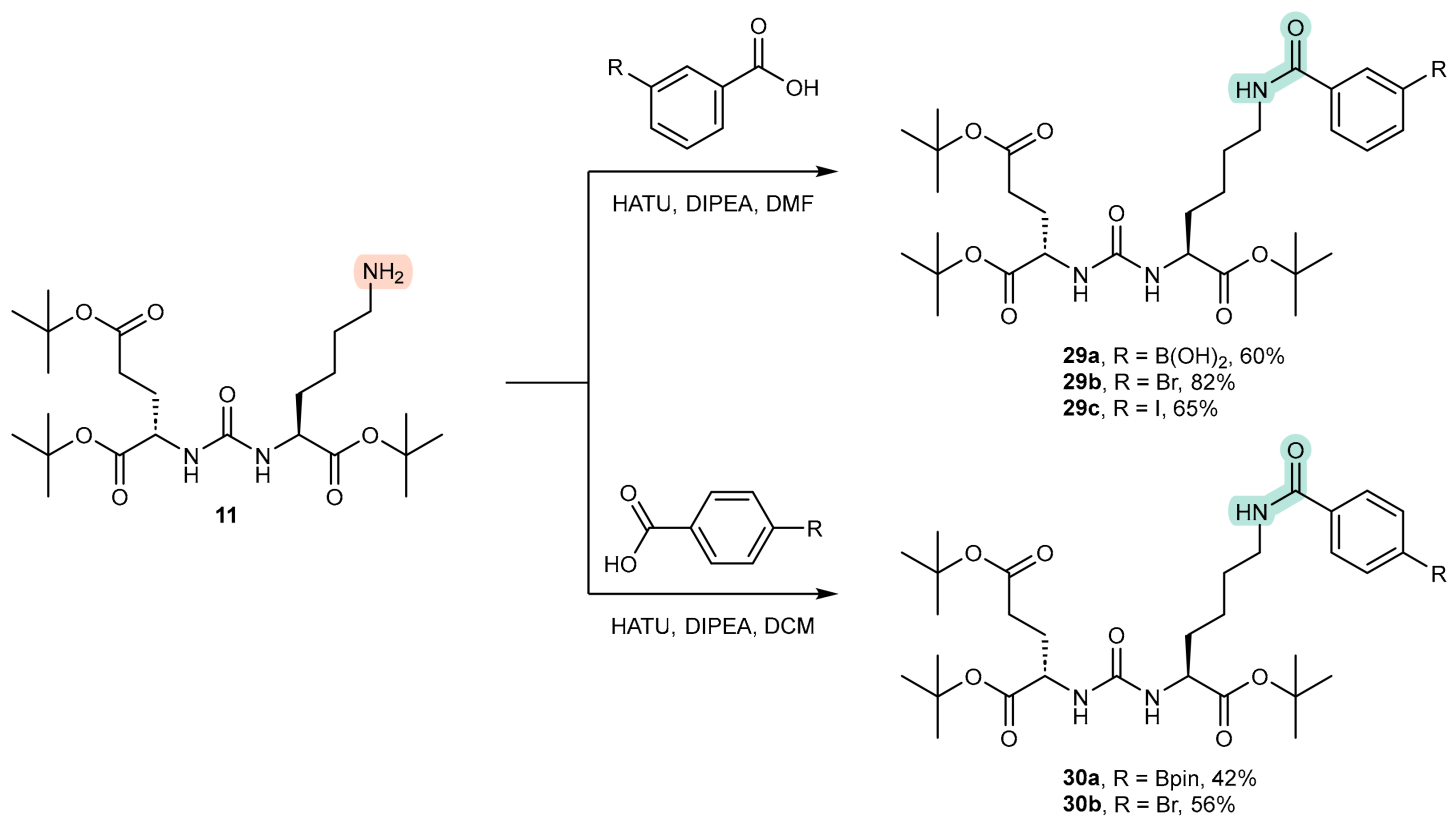
Synthesis of **29a**–**c** and **30a**,**b** by acylation in the presence of HATU.

A similar technique for acylation of protected urea DCL was proposed in [95]. The authors introduced three different *meta*-substituted benzoic acids into the acylation reaction with compound **11** (Scheme 17). HATU was used as an activator, and DIPEA was used as the base. This method made it possible to obtain compounds **29a**–**c** with 60–82% yields.

The approach with HATU has also been used to produce derivatives **30a**,**b** [45]. In contrast to the method of synthesis of compounds **29a**–**c**, DCM rather than DMF was used as a solvent. Derivatives **30a**,**b** were isolated in 42% and 56% yields, respectively.

**Scheme 18:**
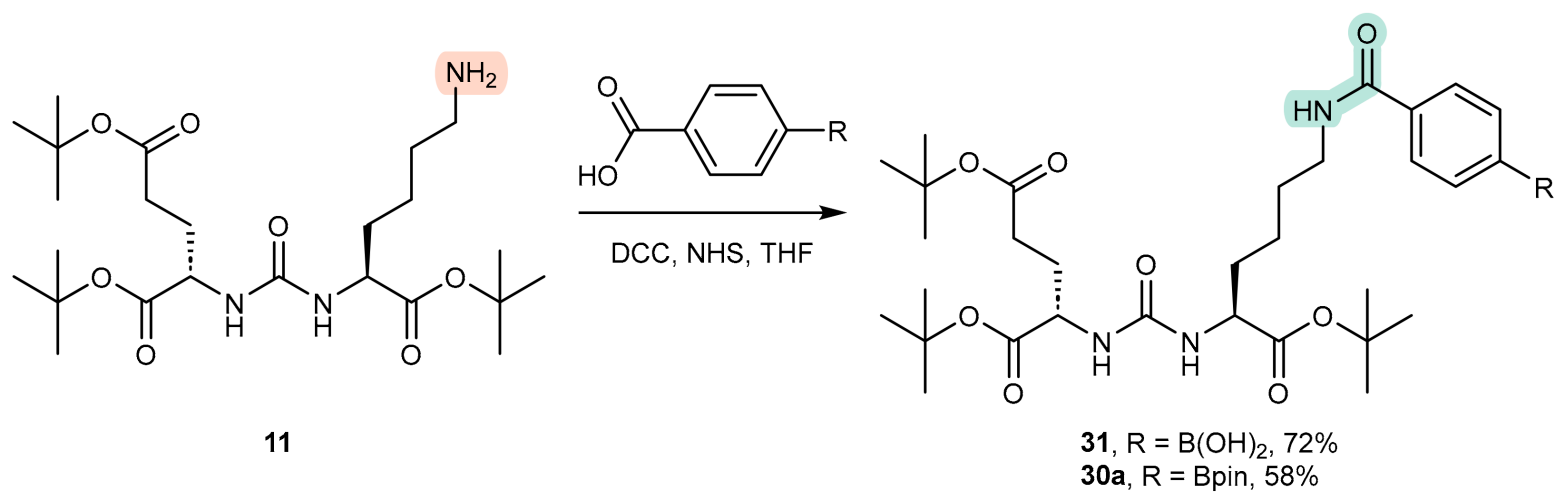
Synthesis of boron-containing DCL derivatives.

Carbodiimides can also be used to produce acylated derivatives of compound **11**. In [96], the authors synthesized boron-containing urea derivatives DCL **30a** and **31** (Scheme 18). The reaction was carried out in the presence of DCC and NHS and the yield of compound **30a** was 58%, which is slightly higher than using the method shown in Scheme 17.

**Scheme 19:**
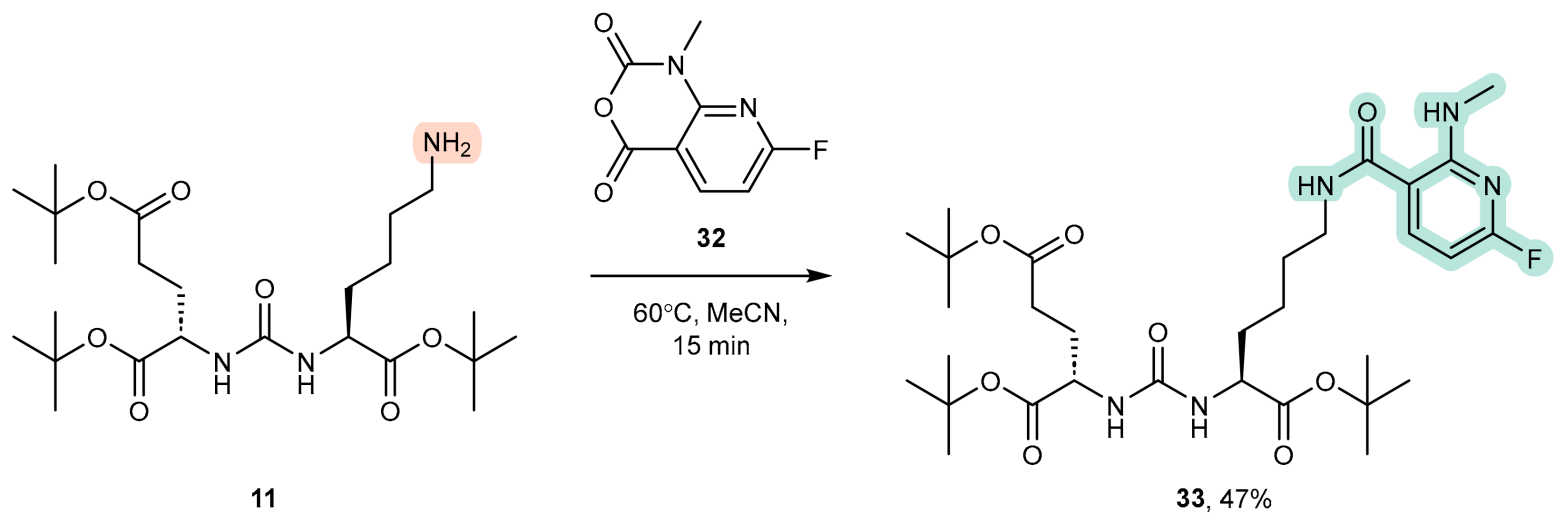
Preparation of compound **33** by acylation of protected urea **11** with anhydride **32**.

Other less common methods for acylation of the amino group of lysine are presented in the literature. In [97], the authors carried out the acylation of the ε-position of lysine by reaction with 7-fluoro-1-methyl-8-azaizotoic anhydride **32** (Scheme 19). An experiment with a natural fluorine isotope made it possible to obtain compound **33** with a yield of 47%, and the reaction time was only 15 min. Subsequent performance of this reaction with derivative **32** containing isotope ^18^F allowed obtainment of labeled derivative **33** with radiochemical conversion > 95%.

**Scheme 20:**
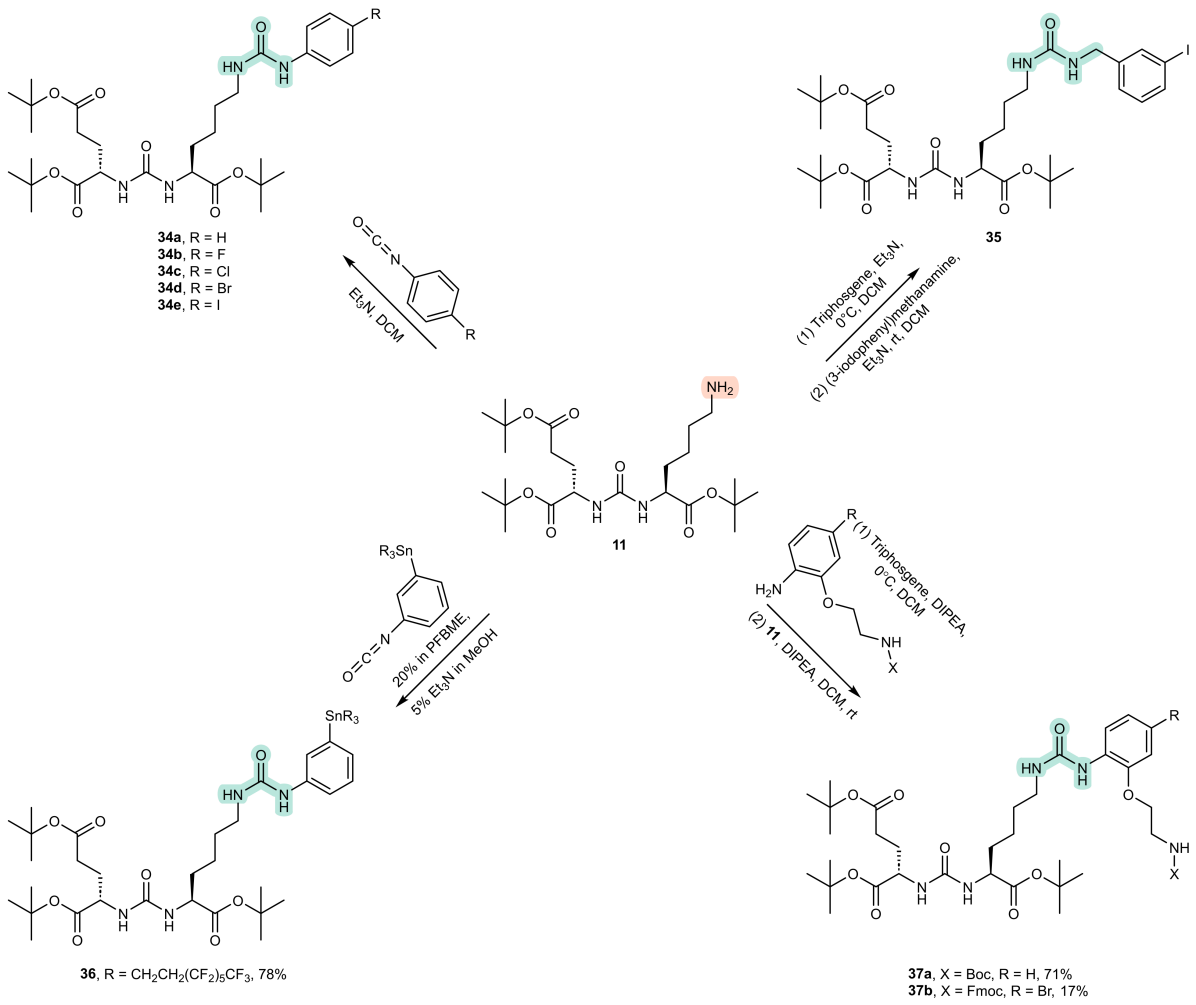
Approaches to modification of DCL by creating a urea bridge.

The next approach for modifying the DCL ε-amino group by introducing an aromatic fragment involves the creation of a urea fragment (Scheme 20). In [46], the synthesis of compounds **34a**–**e** and **35** is described. The authors did not provide the values of the yields for this stage. In the case of a series of compounds, **34**, the corresponding phenylisocyanates were used as reagents. In the case of compound **35**, compound **11** was reacted with triphosgene, after which substituted benzylamine was added to the in situ generated isocyanate.

In [98], the authors introduced an aromatic fragment with a tin-containing group into the structure, suitable for further substitution with ^125^I. The reaction of protected urea **11** with the corresponding isocyanate made it possible to obtain compound **36** with 75% yield. It is worth noting that the reaction was carried out in non-standard solvents: the initial isocyanate was dissolved in perfluorobutyl methyl ether (PDBME), and the reaction itself was carried out in a 5% triethylamine solution in methanol. The use of PFBME was due to the fact that the initial isocyanate is stable under these conditions for more than 20 days.

The triphosgene approach was also used in [85], where substituted aniline is introduced into the reaction with triphosgene instead of urea **11**. In the case of compound **37a**, a yield of 71% was achieved, but for compound **37b**, the yield was only 17%. The authors noted that the low yield is due to the low conversion of the starting compounds, rather than losses during product purification.

**Scheme 21:**
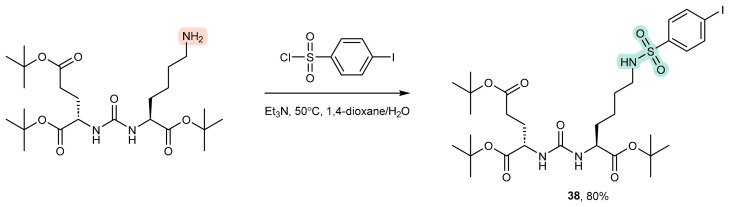
Synthesis of modified DCL urea with a sulfamide fragment.

Currently, modifications with the creation of a sulfamide fragment between the amino group of lysine and the aromatic fragment are the least widespread. In [46], a method for the synthesis of compound **38** was proposed, involving the introduction of protected urea **11** into a reaction with the corresponding sulfonyl chloride (Scheme 21). The product was obtained with a fairly high yield of 80%. However, such modifications were not considered in the literature in the future.

The last approach to the introduction of aromatic fragments in this position assumes the acylation of the ε-amino group of lysine with various amino acids containing an aryl substituent in the side chain. Often this amino acid fragment is included in the structure of the linker, which is synthesized separately. In some cases, the introduction of this fragment can be carried out using solid-phase techniques. Such examples will be discussed in Section 3.2 and Section 4. Here we will consider the simplest examples of methods involving the acylation of protected urea with amino acids.

**Scheme 22:**
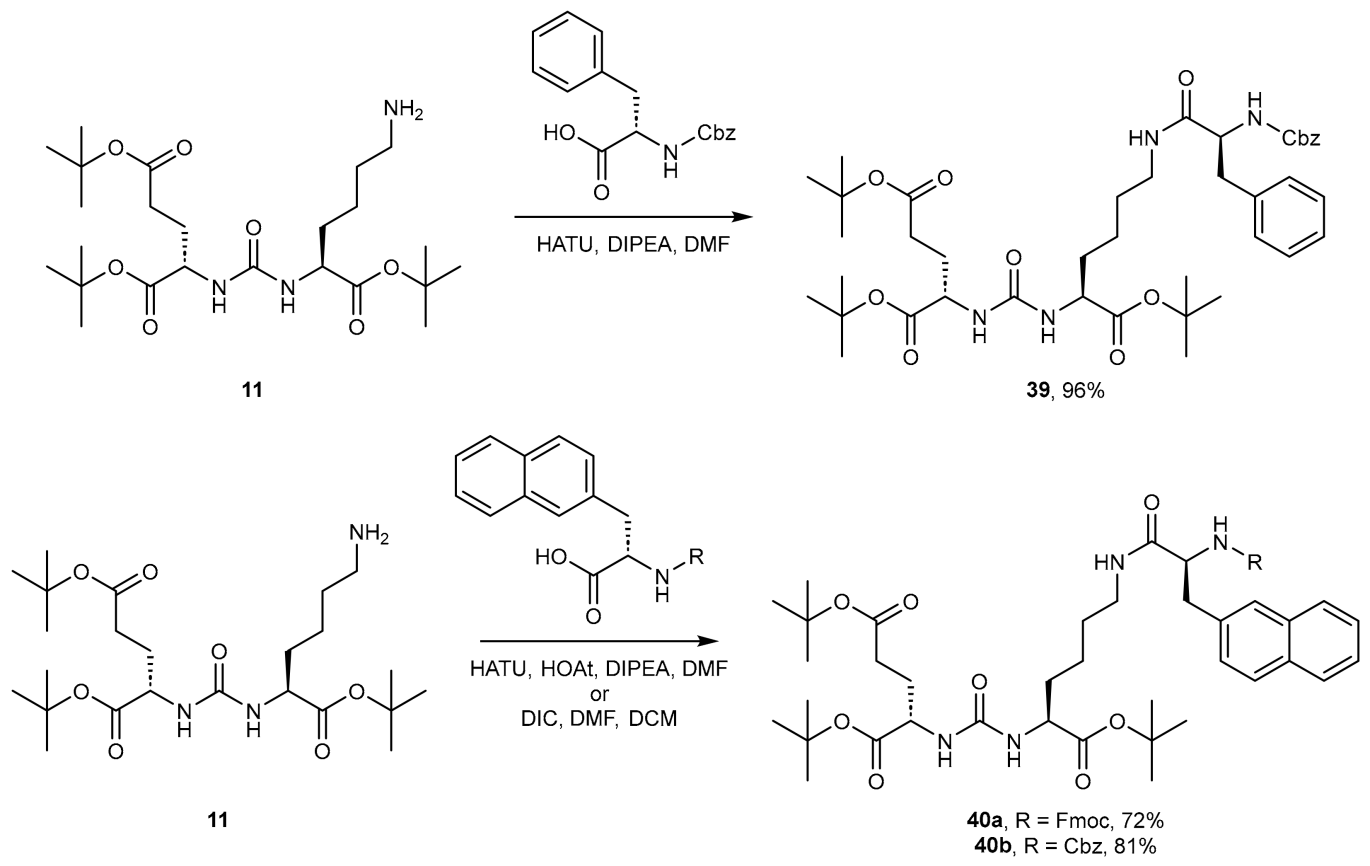
Synthesis of compounds **39** and **40** using various activators.

In [61], the ε-amino group of lysine was acylated by Cbz-protected *L*-phenylalanine in the presence of HATU and DIPEA (Scheme 22). The proposed technique made it possible to obtain compound **39** with a 96% yield.

HATU was also used in the preparation of compound **40a** containing a fragment of *L*-naphthylalanine [63]. The reaction was carried out in the presence of HOAt, which is generally not necessary for this example. The product yield was 72%.

Carbodiimides can be used instead of HATU at this stage. In [99], the authors performed liquid-phase synthesis of PSMA-617 and used DIC to introduce the naphthylalanine fragment. Using such conditions, it was possible to obtain compound **40b** with an 81% yield.

**Scheme 23:**
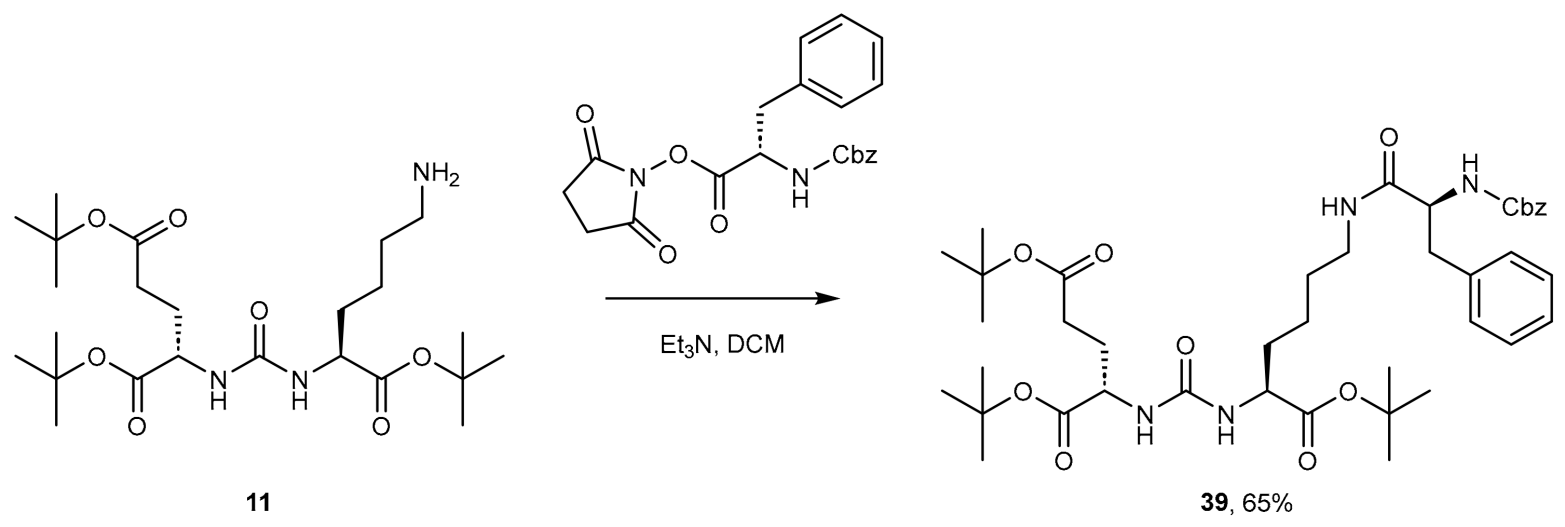
Synthesis of compound **39** using NHS-ester.

It is also possible to use NHS-esters for this reaction. A similar approach was used to obtain compound **39** in [100,101] (Scheme 23). The yield of the target product was 65%, which is slightly lower than with HATU. In both cases, purification was carried out using chromatography.

Thus, acylation and alkylation of the DCL lysine ε-amino group are the most commonly used approaches. No optimal reductive amination protocol exists, as yields vary widely (12–~90%) depending on substituents of the aryl ring. NaBH_4_ outperforms STAB/NaBH_3_CN in select cases, though benzaldehyde reduction to benzyl alcohol lowers efficiency and increases labor.

TFP/NHS-esters of substituted benzoic acids provide slightly higher acylation yields (65–95%) than HATU/TBTU activation methods (42–82%). Their stability enables scale-up, despite added synthesis steps and variable storage stability. For amino acids with aromatic side chains, the reverse trend is observed—NHS-esters provide lower yields than HATU.

### 3.2. Methods of Introducing the Linker into the Structure of the PSMA Ligand

The linker, the next key fragment influencing PSMA ligand inhibitory activity, varies widely in length, structure, rigidity, and synthesis. This section surveys linker synthesis strategies.

**Figure 5:**
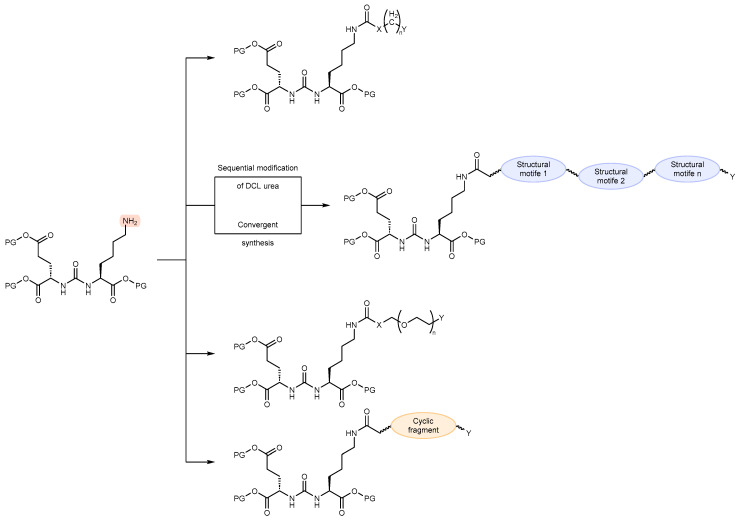
General approaches to linker introduction. X = CH_2_, NH or O; Y = functional group for future conjugation.

Linker choice depends on target conjugate design. Approaches can be classified by fragment complexity: simple linkers; complex linkers (sequential DCL modification or convergent synthesis); PEG-containing linkers; and conformationally rigid cyclic linkers (Figure 5). It should be noted that this classification is not strict—some examples span multiple categories.

One of the simplest approaches to linker design assumes the presence in the structure of an alkyl fragment of the required length with a terminal functional group for subsequent modification. It can be a dicarboxylic acid, an acid with a terminal amino group, or another compound with a terminal group suitable for subsequent modification.

**Scheme 24:**
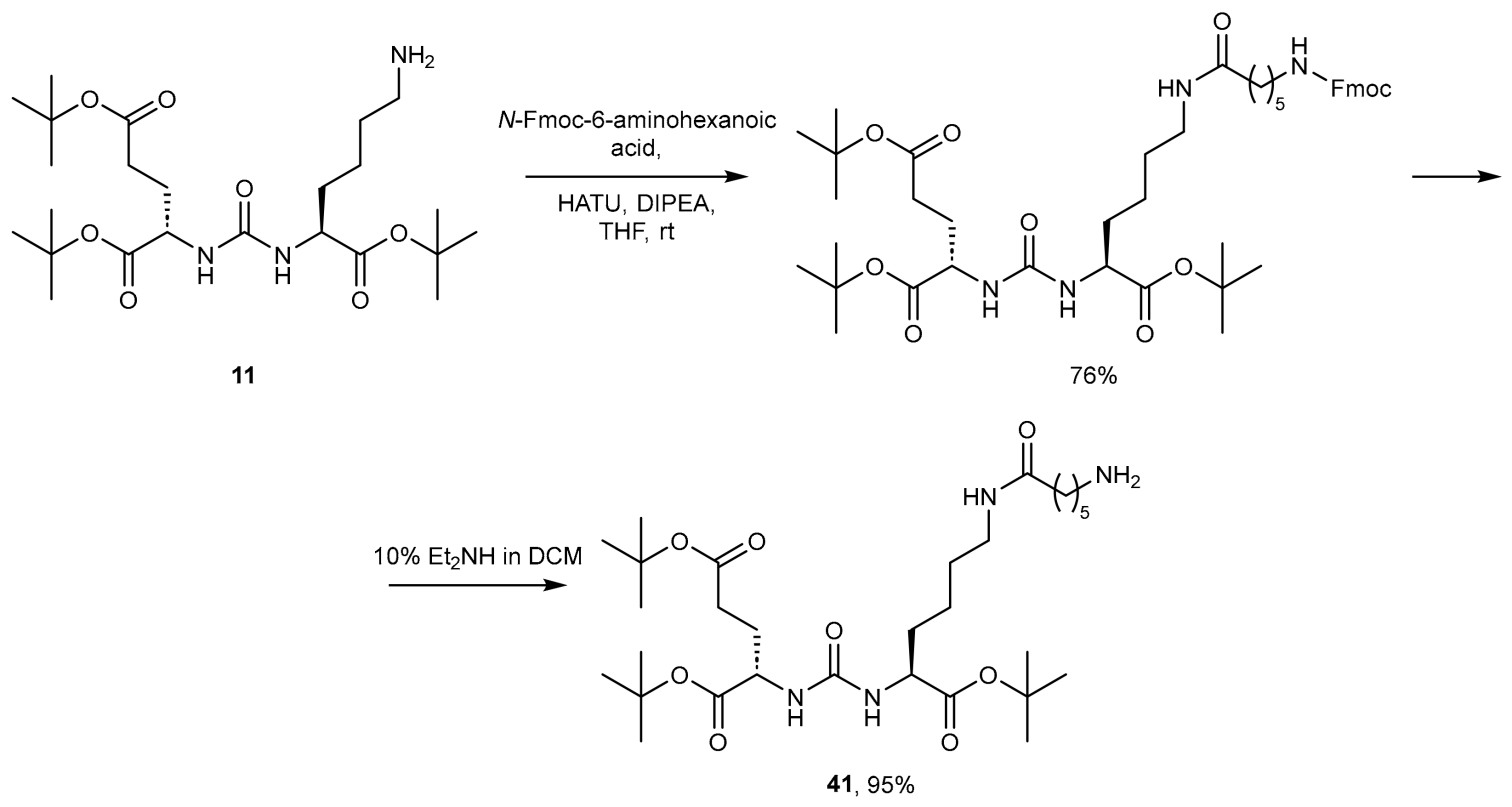
Synthesis of compound **41**.

One of the simplest examples of this approach is the work [48], which presents a method for obtaining compound **41** with a 6-aminohexanoic acid residue as a linker (Scheme 24). The authors in the first stage acylated urea **11** of Fmoc-protected 6-aminohexanoic acid, and after that removed the protective group under the action of diethylamine to obtain compound **41**. The yield in two stages was 72%.

Also, due to acylation, the fragments containing a triple bond can be introduced into the structure for the subsequent azide-alkyne cycloaddition reaction.

**Scheme 25:**
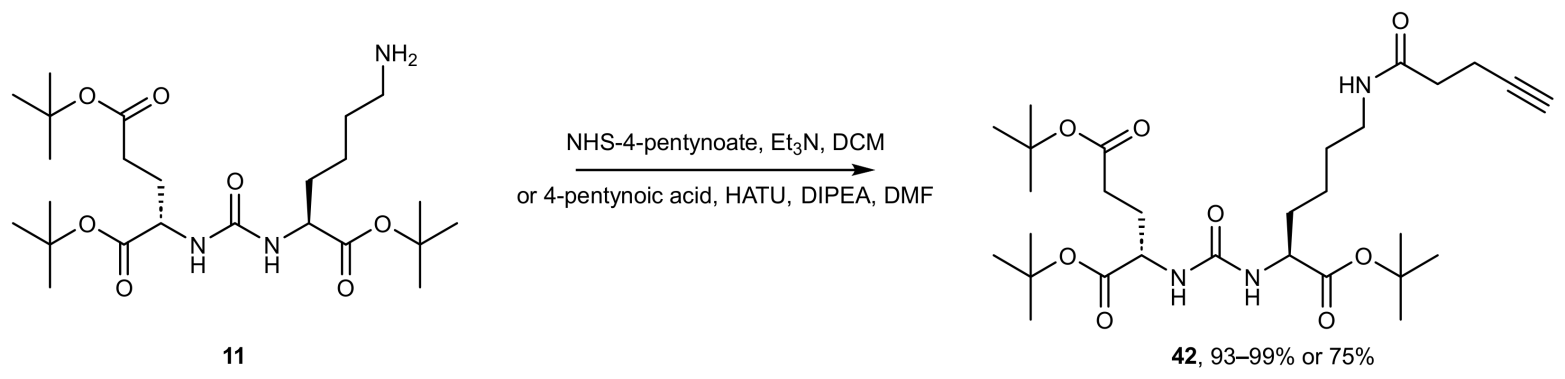
Methods of acylation of compound **11** with 4-pentynoic acid.

In [55,102], the authors presented a method for acylation of compound **11** with the NHS-ester of 4-pentynoic acid to obtain compound **42** (Scheme 25). In both cases, the reaction proceeded with high yields (93 and 99%). In [103], a method for obtaining compound **42** was also proposed. HATU was used as the activator, and DIPEA was used as the base. The proposed method showed a slightly lower yield compared to NHS-ester (75%).

**Scheme 26:**
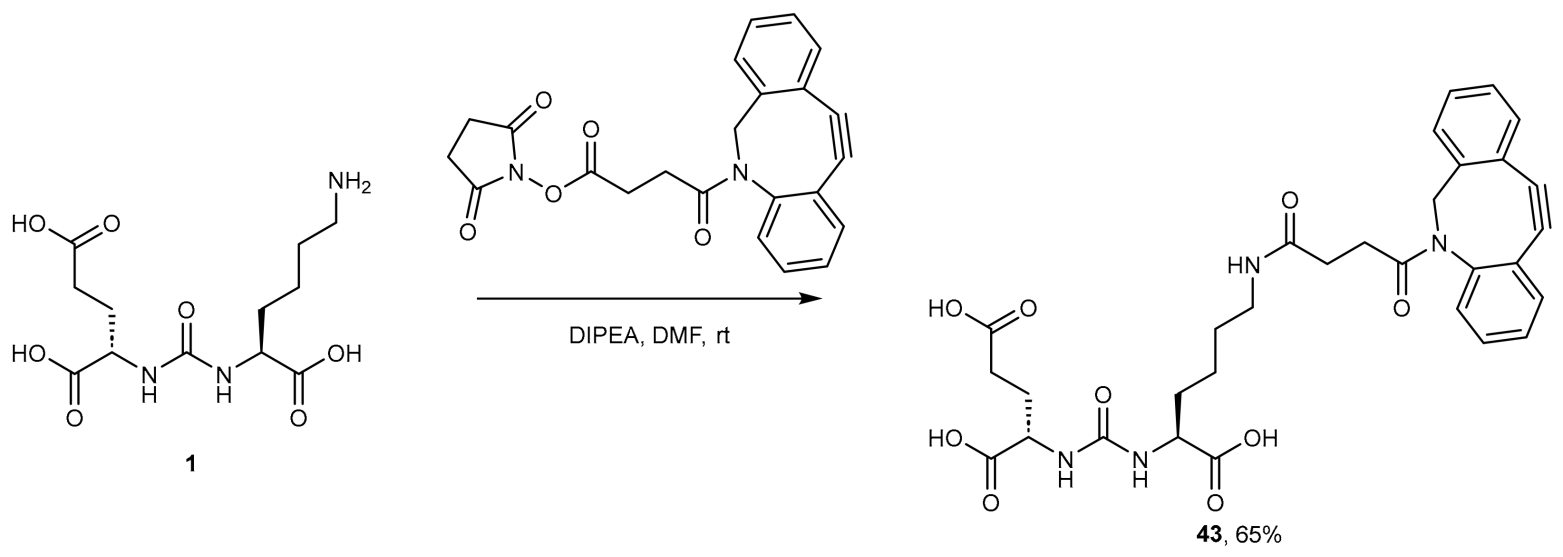
Introduction of the fragment 11,12-didehydro-5,6-dihydrodibenz[*b*,*f*]azocine into the structure of the PSMA ligand.

Not only the terminal triple bond can act as a functional group for the subsequent reaction of the azide-alkyne cycloaddition, in [104], the authors synthesized compound **43** containing a fragment of 11,12-didehydro-5,6-dihydrodibenz[*b*,*f*]azocine for subsequent modification by a click reaction (Scheme 26). The NHS-ester of modified 11,12-didehydro-5,6-dihydrodibenz[*b*,*f*]azocine was used as an acylating agent, and the reaction was carried out in DMF in the presence of DIPEA. The target compound was obtained with a yield of 65%.

**Scheme 27:**
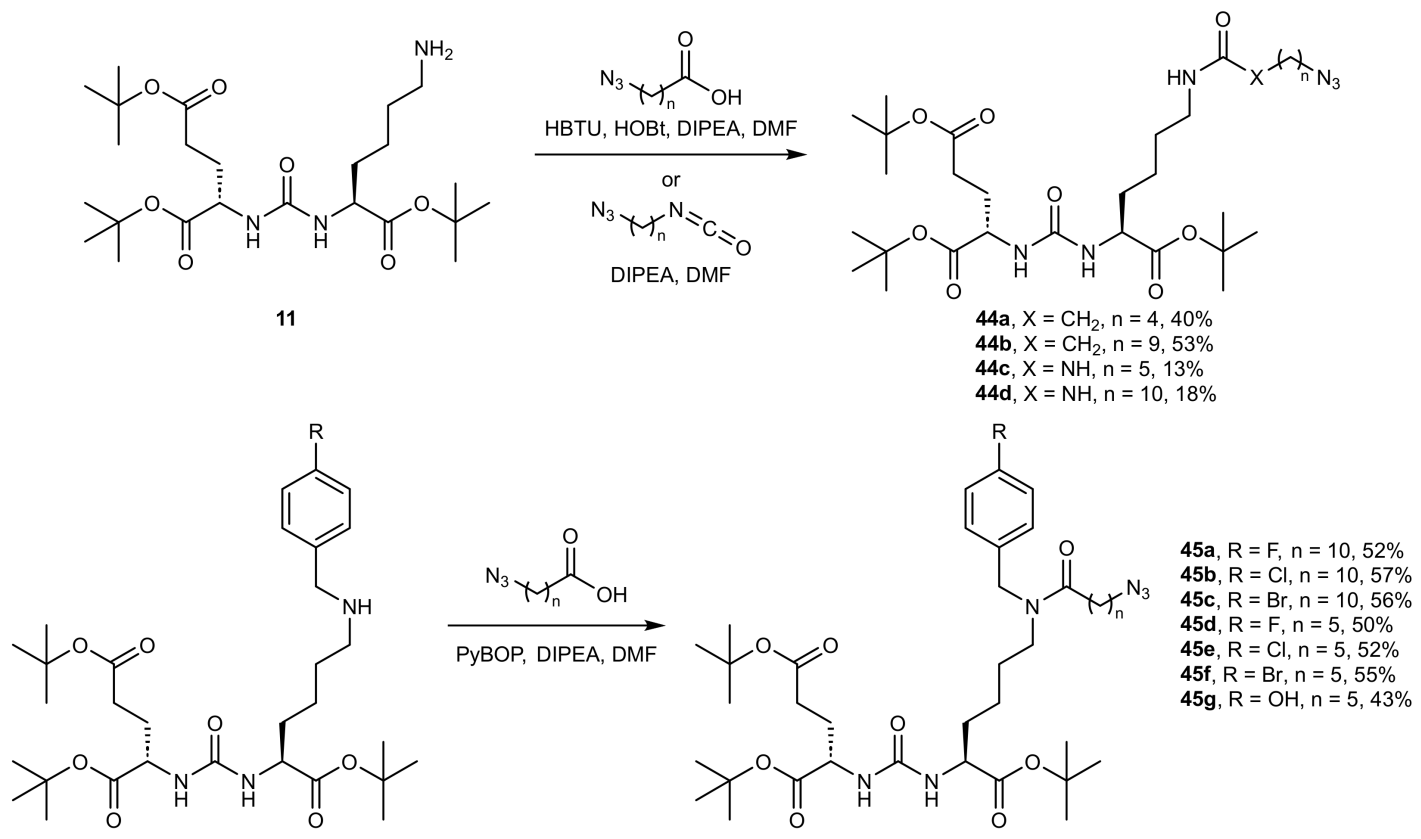
The introduction of the azide group into the structure of PSMA ligands proposed in [37].

Instead of a triple bond, an azide group can be introduced into the ligand structure. In [37], the authors introduced azide groups into the ligand structure by the acylation reaction of compound **11** or its derivatives with the corresponding azide-containing acids (Scheme 27, compounds **44a**,**b**, **45a**–**g**). Also, due to the reaction of protected urea with isocyanates, two **44c**,**d** derivatives containing a urea fragment were obtained. These compounds were obtained with rather low yields (13 and 18%), respectively. It is worth noting that in the case of compounds **44a**,**b**, the acylation reaction was carried out in the presence of HBTU and HOBt, and in the case of a series of compounds, **45**, PyBOP was used as a coupling agent. PyBOP use is justified by the higher reactivity of its active species (vs. HBTU) when coupling sterically hindered secondary amines. Both proposed techniques made it possible to obtain protected PSMA ligands with average yields of 40–57%.

**Scheme 28:**
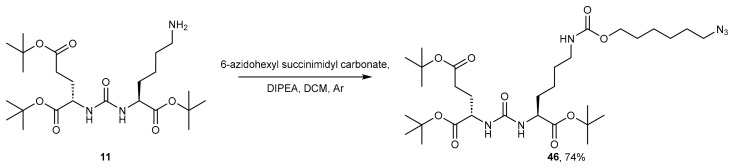
Carbamate synthesis **46**.

The “azide” approach was realized in [105]. The authors proposed a method for obtaining carbamate **46** with a terminal azide group (Scheme 28). Compound **11** was reacted with 6-azidohexyl succinimidyl carbonate in the presence of DIPEA and the target product was isolated with 74% yield.

**Scheme 29:**
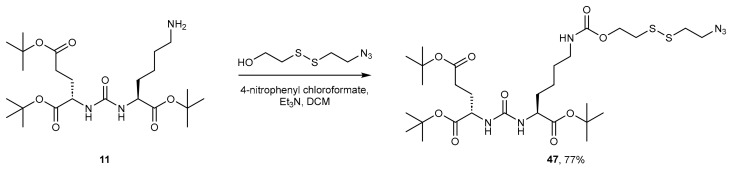
Synthesis of compound **47**.

Azide **47** with a carbamate fragment was also obtained (Scheme 29) [106]. In contrast to the synthesis of compound **46**, in this case the carbamate bond was formed by the interaction of the initial compound **11** and alcohol with 4-nitrophenyl chloroformate. The reaction yield was 77%. Compound **47** does not contain a simple alkyl linker, but a disulfide fragment cleaved by the action of intracellular glutathione.

**Scheme 30:**
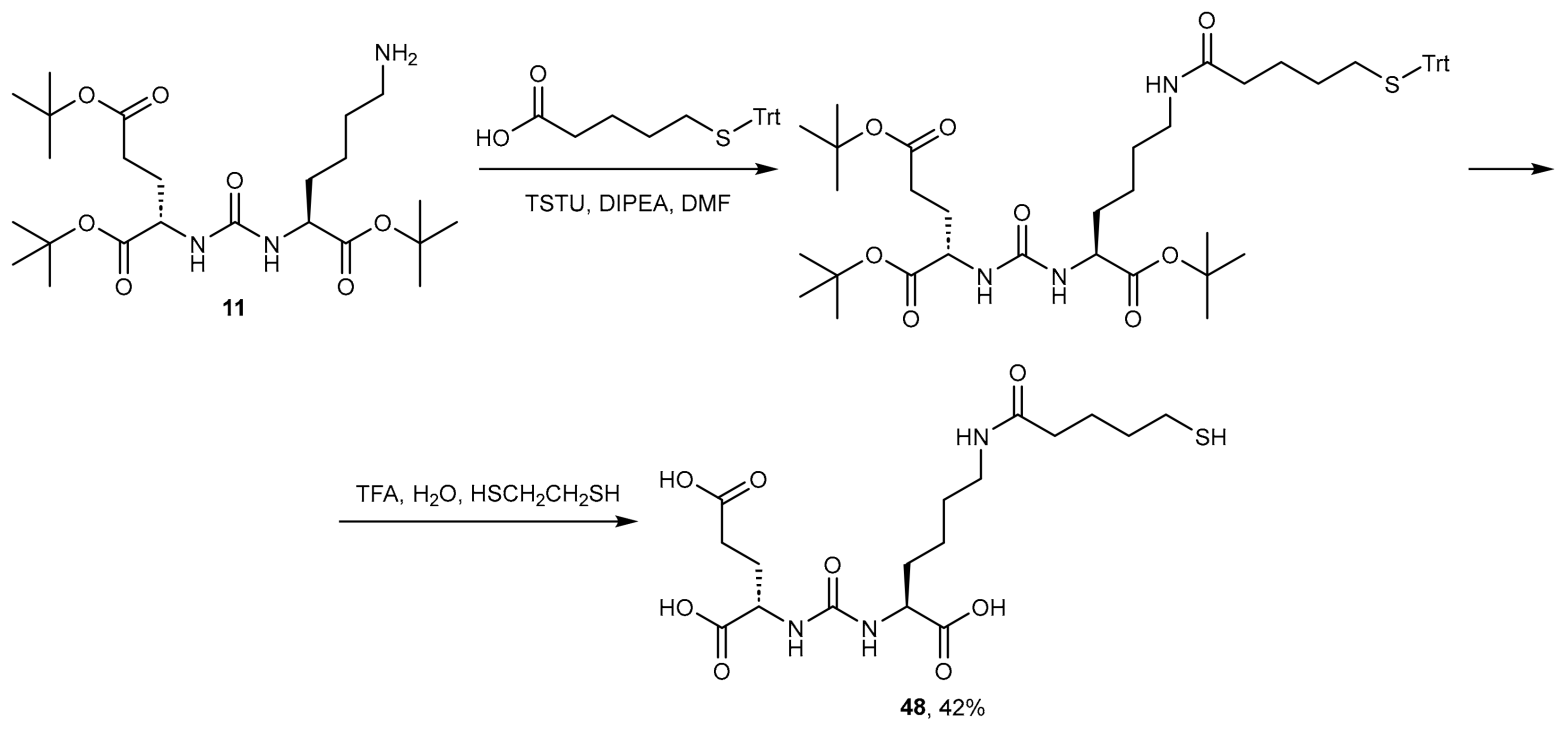
Preparation of a thiol-containing ligand **48**.

The thiol group can act as a terminal fragment for subsequent modification. For example, in [38], the authors presented the synthesis of ligand **48** containing a fragment of 5-mercaptopentanoic acid (Scheme 30). In the first stage, urea **11** was acylated with trityl protected acid in the presence of TSTU and DIPEA. After that, acid-labile protective groups were removed under the action of trifluoroacetic acid in the presence of water and ethanediol. The total yield of target compound **48** was 42%.

**Scheme 31:**
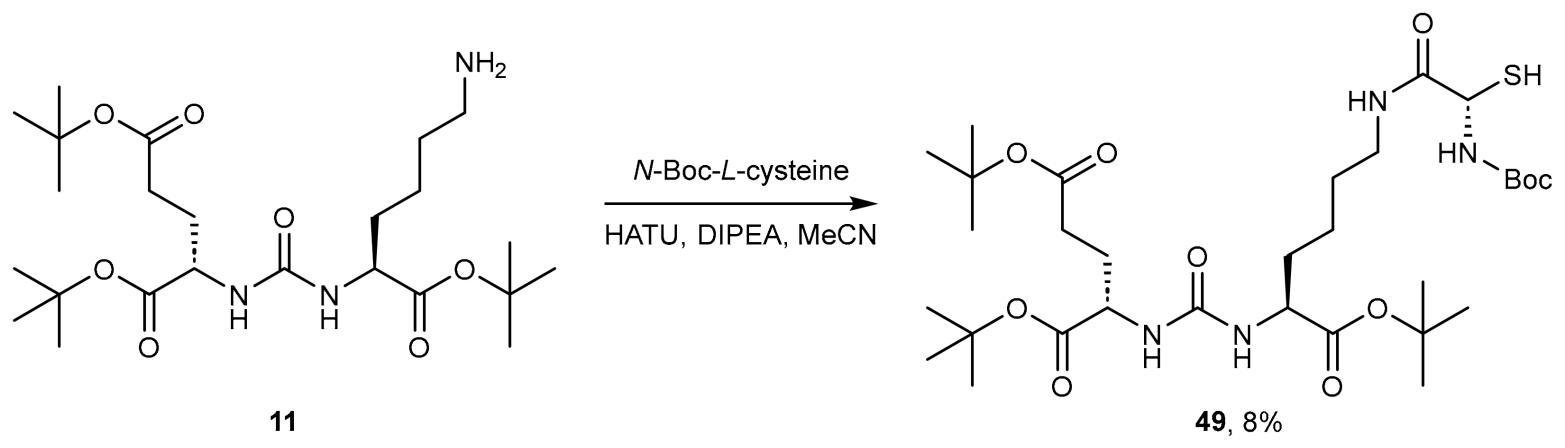
Synthesis of compound **49**.

In some cases, to introduce a thiol group into the ligand structure, the ε-amino group is acylated with a thiol-containing amino acid (for example, cysteine). Such a modification was carried out in [82]. The authors performed an acylation reaction of compound **11** with *N*-Boc-*L*-cysteine in the presence of HATU and DIPEA, but the yield of the target product **49** was only 8% (Scheme 31). The low yield of the proposed method is likely due to disulfide formation as the major side product.

**Scheme 32:**
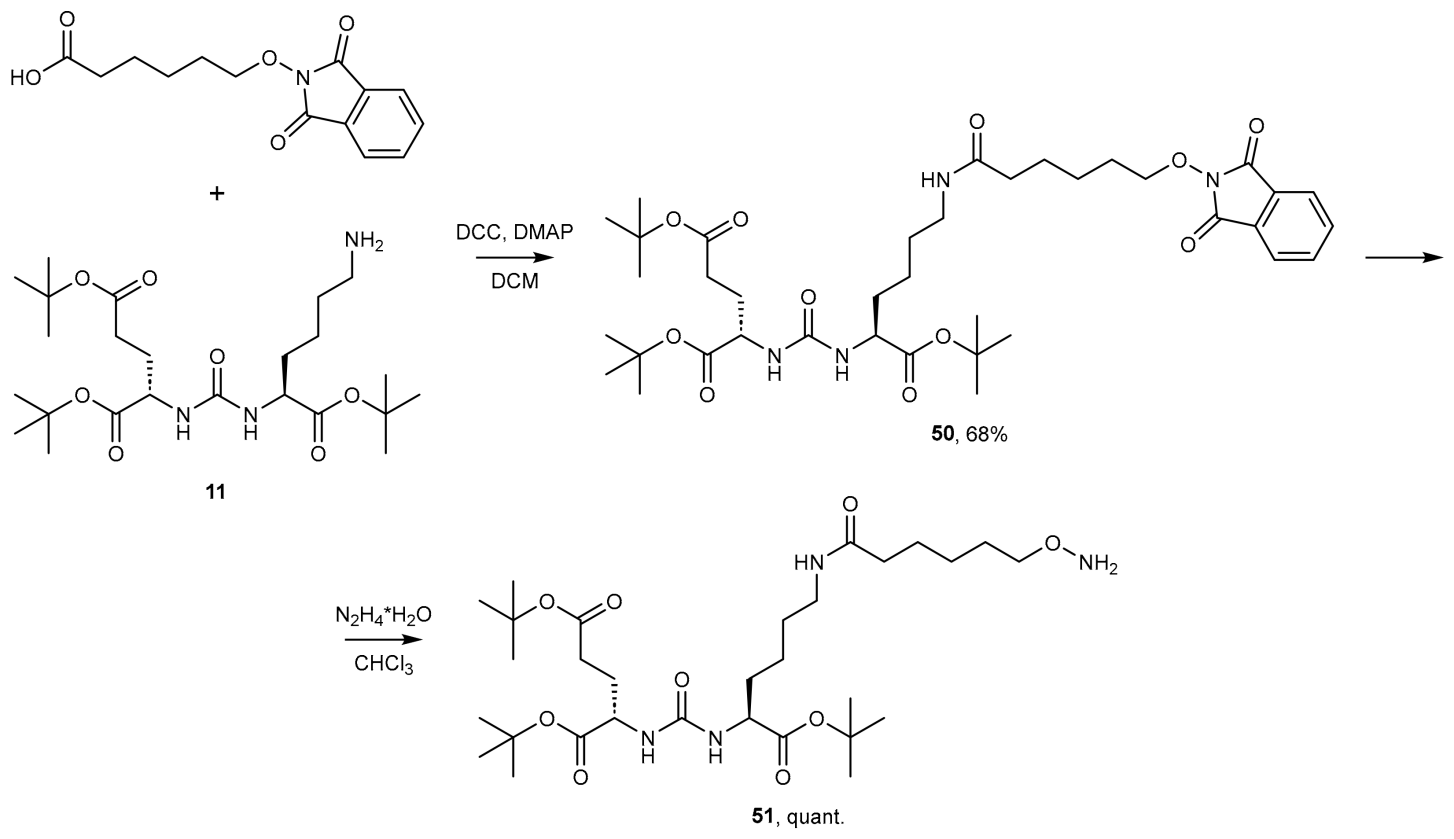
Synthesis of the protected PSMA ligand **51**.

In [107], a fragment containing *O*-alkyl hydroxylamine was introduced into the structure of the PSMA ligand (Scheme 32). Urea **11** was acylated with an acid containing an *N*-alkoxypthalimide fragment in the presence of DCC and DMAP. The derivative **50** obtained with a yield of 68% was then reacted with hydrazine hydrate in chloroform. In this way, compound **51** was obtained in a quantitative yield. Such linker was introduced into the structure for further conjugation of the ligand with the platinum complex due to the formation of oxime.

In summary, acylation of the DCL lysine ε-amino group is most prevalent, though yields depend primarily on the introduced fragment. Reaction conditions should match the linker structure. Urea or carbamate linkages offer amide alternatives, though urea derivatives give modest yields requiring optimization. Carbamate synthesis is better developed (>70% yield), making it viable for linker introduction.

**Scheme 33:**
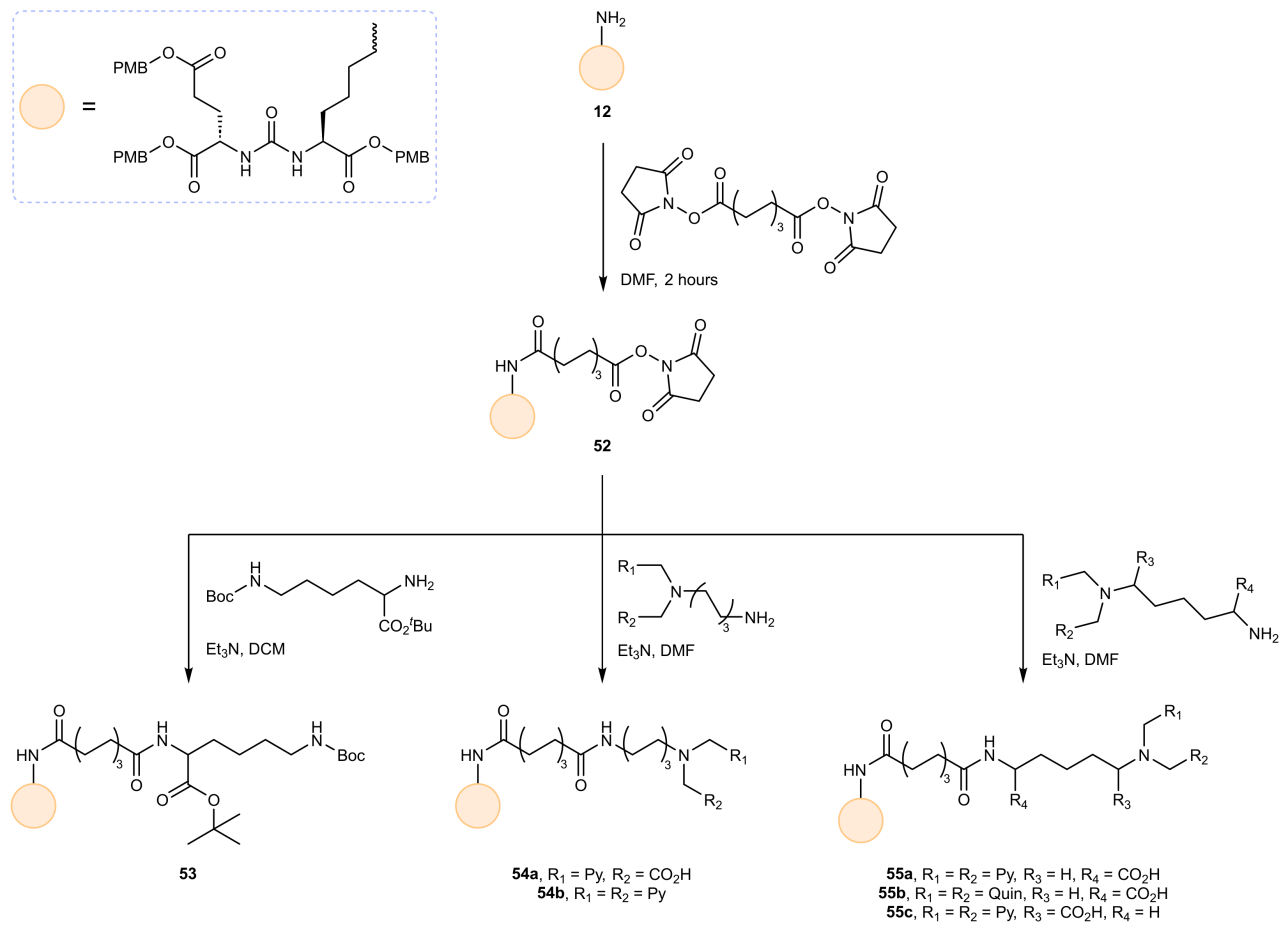
Approaches to the synthesis of PSMA-directed compounds by acylation of the ε-amino group of lysine with DSS.

Earlier discussed linker introduction strategies focused on simple structural motifs. More complex linker designs modify protected DCL urea at the ε-amino group, followed by attachment of pre-synthesized fragments. For example, such an approach was implemented by acylating protected urea **12** with suberic acid disuccinimidyl (Scheme 33). The resulting derivative **52** can then be subjected to a wide range of modifications. Thus, in [108], the authors acylated protected lysine with compound **52** to obtain derivative **53**. A similar approach was also used in [71] to obtain compounds suitable for chelating technetium and rhenium **54a**,**b** and **55a**–**c**.

An approach involving the introduction of a suberic acid ligand into the structure and subsequent modification of its carboxyl group was also implemented in [73,109,110,111] for the preparation of other conjugates containing chelating fragments or fluorescent labels. However, unlike the procedure shown in Scheme 33, compound **1** was introduced into the acylation reaction with DSS, rather than its protected form.

**Scheme 34:**
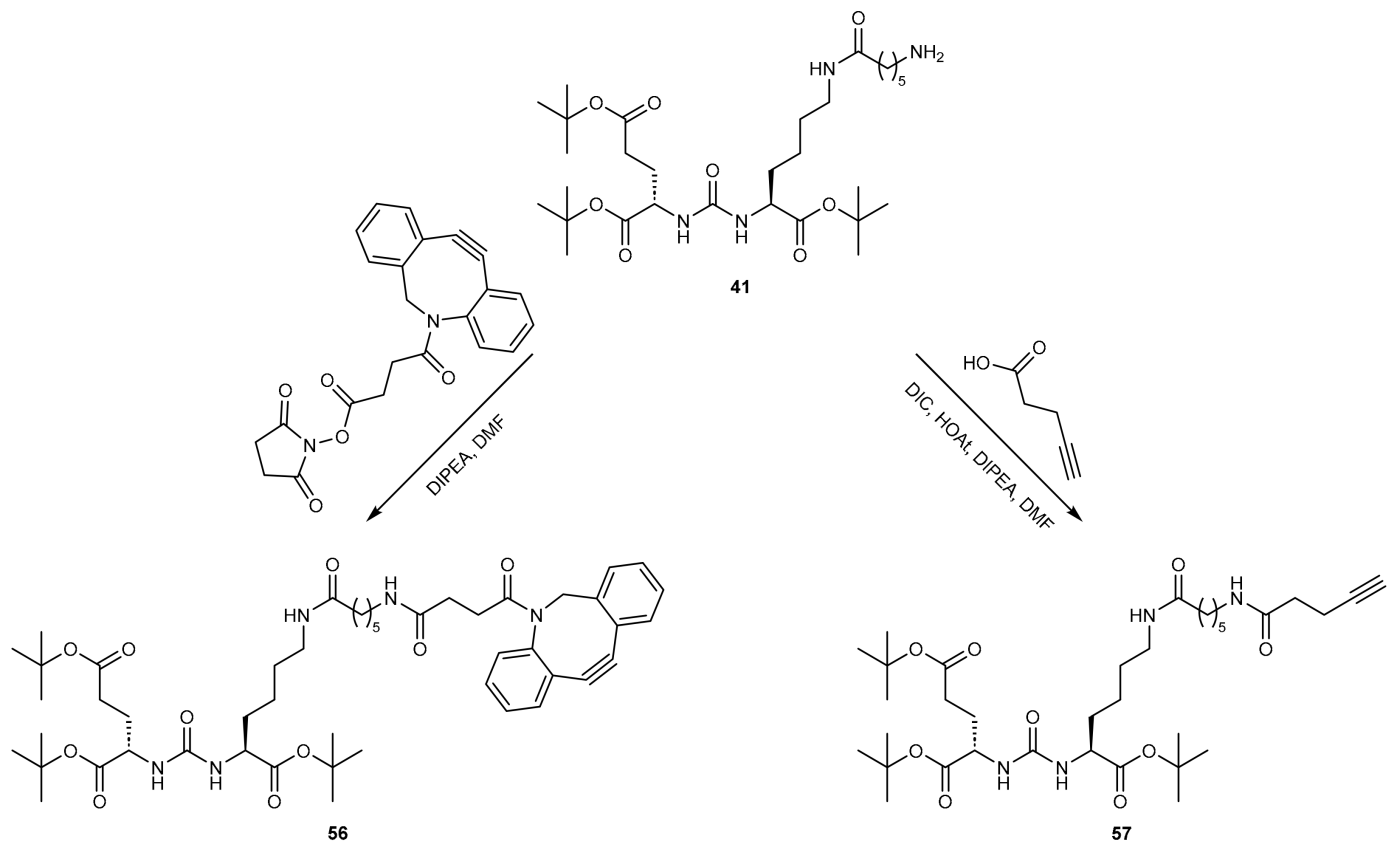
Acylation of amine **41** to obtain compounds **56** and **57**.

Another variant of the composite linker is presented in [112]. The authors introduced an additional fragment into the linker containing a triple bond by acylation of compound **41** (Scheme 34). To introduce the fragment 11,12-didehydro-5,6-dihydrodibenz[*b*,*f*]azocine, the NHS-ester of the corresponding acid was taken into the reaction in the presence of DIPEA, which made it possible to obtain compound **56**. The introduction of a fragment of 4-pentynoic acid was carried out by acylation of amine **41** in the presence of di-*iso*-propylcarbodiimide, HOAt and DIPEA, which led to the preparation of compound **57**.

**Scheme 35:**
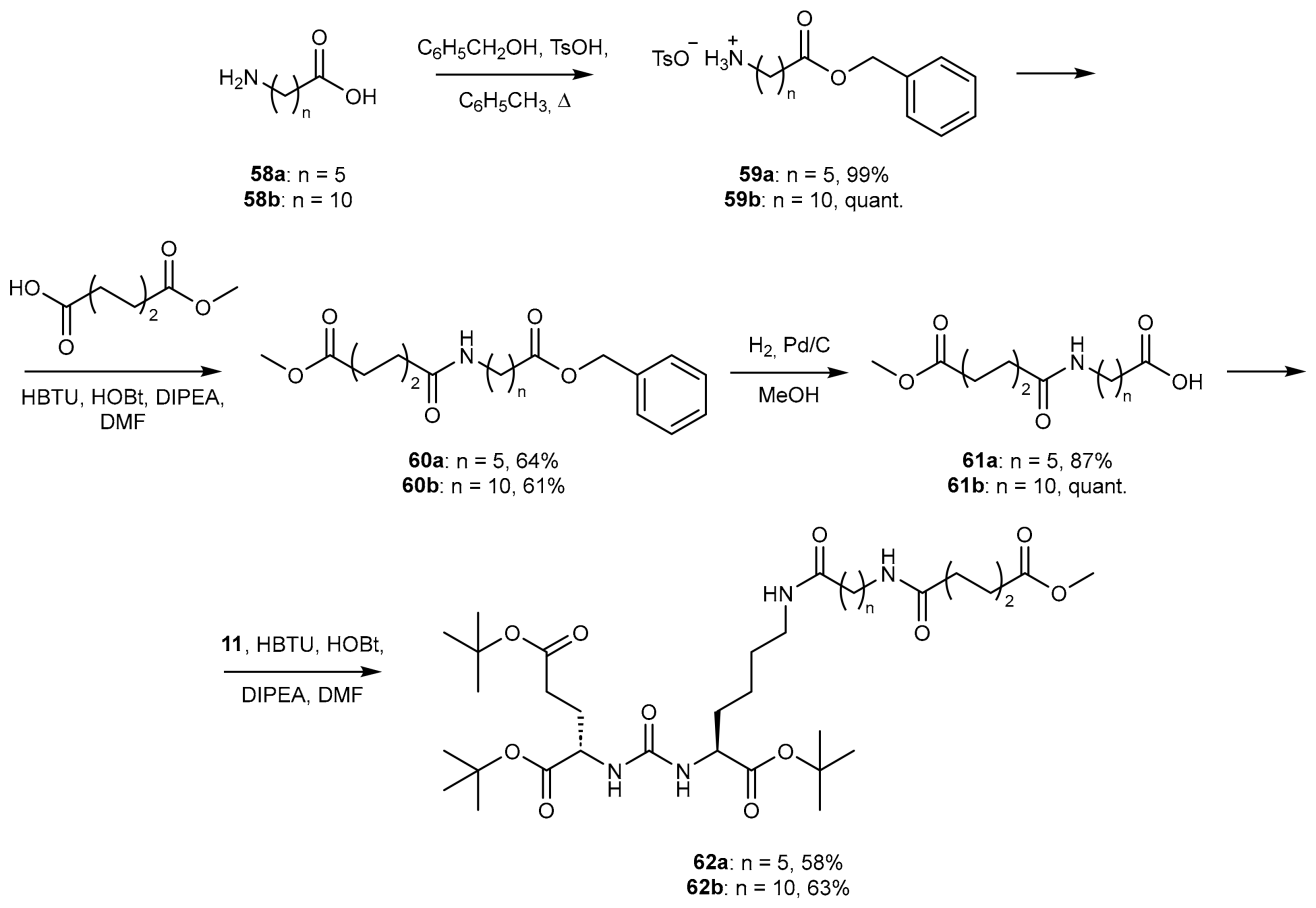
Synthesis of protected ligands **62a**,**b** with a linker with different lengths.

In the case of compounds **56** and **57**, a linker consisting of several fragments was introduced by sequential modification of protected urea **11**. An alternative approach involves the separate synthesis of the linker or its fragment and the subsequent reaction of the synthesized fragment with DCL (or a modified form of urea). A similar synthesis scheme was realized in [39]. The authors carried out the preliminary assembly of the linker based on carboxylic acids with different length of the hydrocarbon chain and terminal amino group **58a**,**b** (Scheme 35). In the first stage, the corresponding benzyl esters **59a**,**b** were obtained, which were acylated with monomethyl ester of adipic acid. The resulting compounds **60a**,**b** were subjected to hydrogenolysis to selectively remove the benzyl protective group, and the carboxylic acids **61a**,**b** synthesized in this way acylated protected urea **11**, resulting in the precursor compounds **62a**,**b** of the PSMA ligands.

**Scheme 36:**
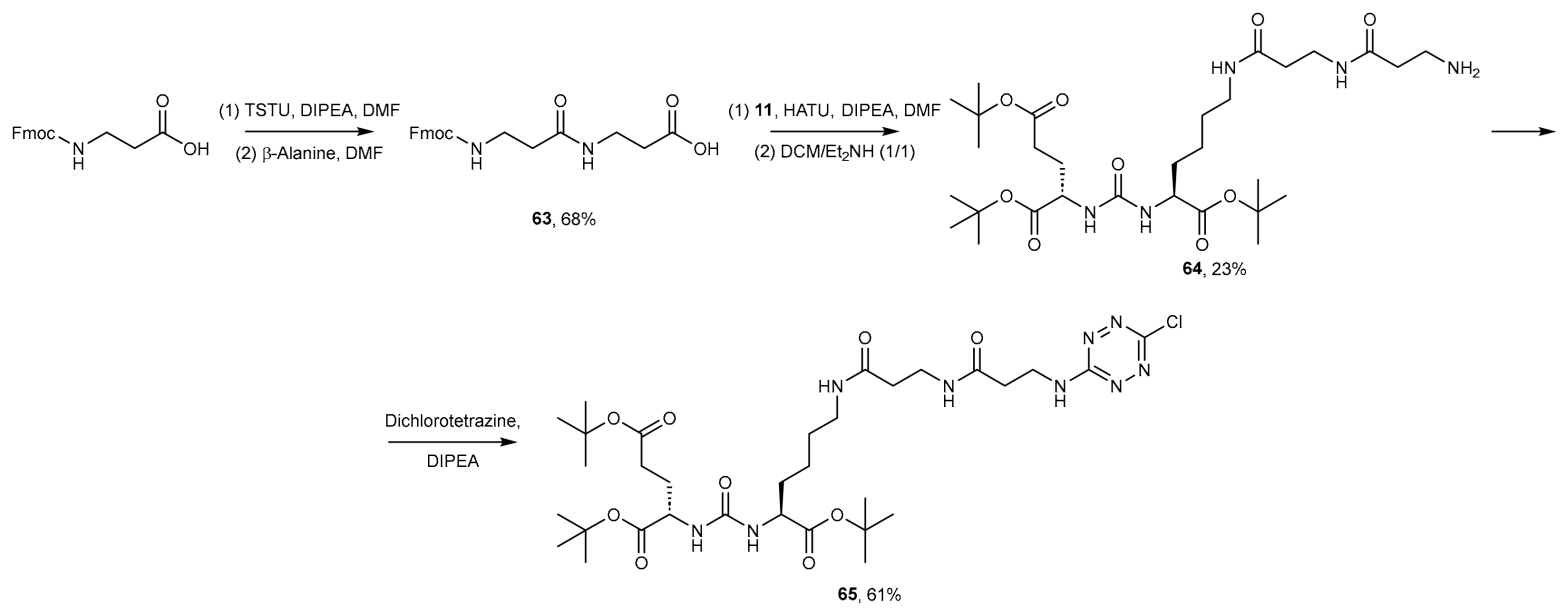
Synthesis of tetrazine-containing compound **65**.

In [113], the authors separately synthesized the linker compound **63** containing two β-alanine residues, after which they introduced it into the acylation reaction with compound **11** (Scheme 36). The resulting product **64** was then reacted with dichlorotetrazine to form a derivative **65** capable of entering into inverse electron-demand Diels–Alder reactions.

**Scheme 37:**
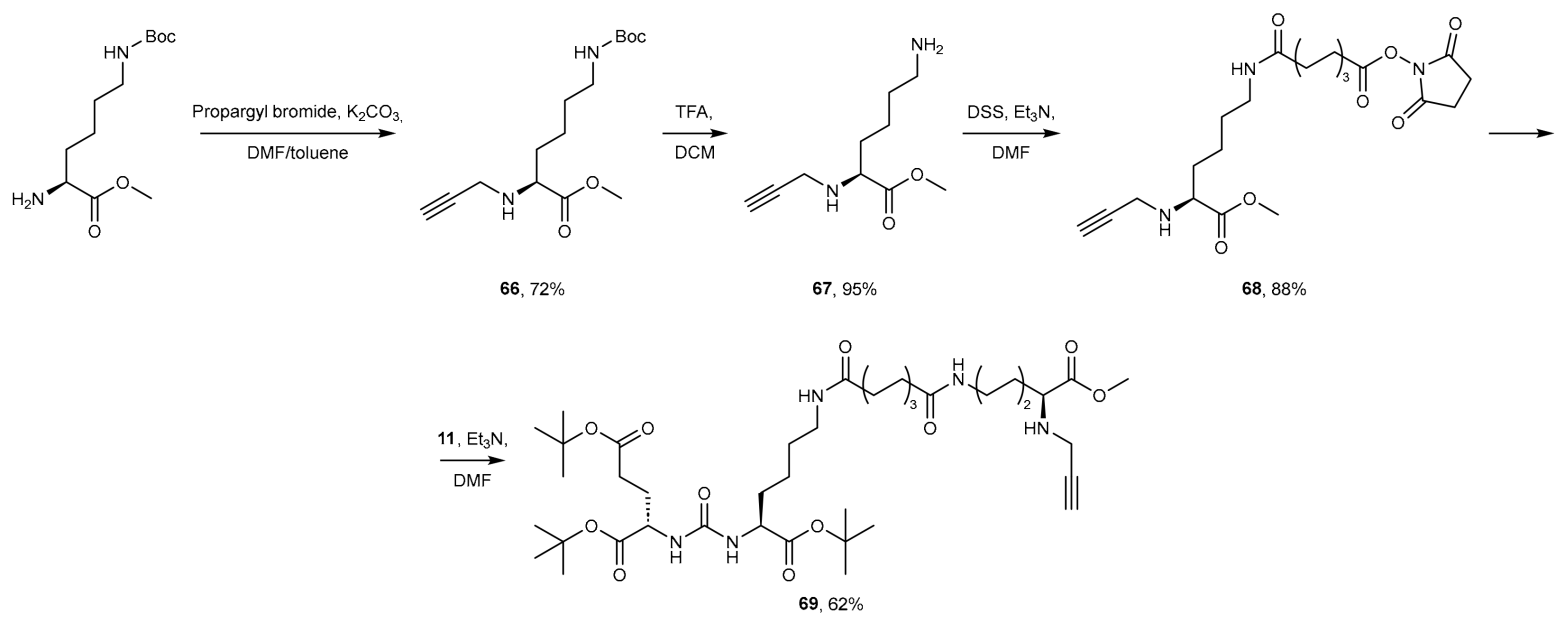
Synthesis of compound **69**.

A linker was also synthesized separately in [52]. To obtain it, *N*-(ε)-Boc-Lys(OMe) was introduced into an alkylation reaction with propargyl bromide to obtain compound **66** (Scheme 37). After that, the Boc protective group was removed to obtain **67**, which in turn was introduced into the acylation reaction with DSS to form NHS-ester **68**. The resulting product was introduced into the acylation reaction with urea **11**. Thus, compound **69** was obtained, which contains suberic acid, lysine, and a terminal alkyne fragment in the linker structure.

It is worth noting that the linker can play a significant role in the inhibitory activity of PSMA ligands. This is due to the presence of hydrophobic pockets in the structure of the tunnel leading to the active site of the protein [84]. The effect of various functional fragments in the linker structure on the biological activity of PSMA ligands and conjugates based on them is considered in detail in the review [21]. In this regard, a large number of GCPII inhibitors with significantly more complex linkers have appeared.

**Scheme 38:**
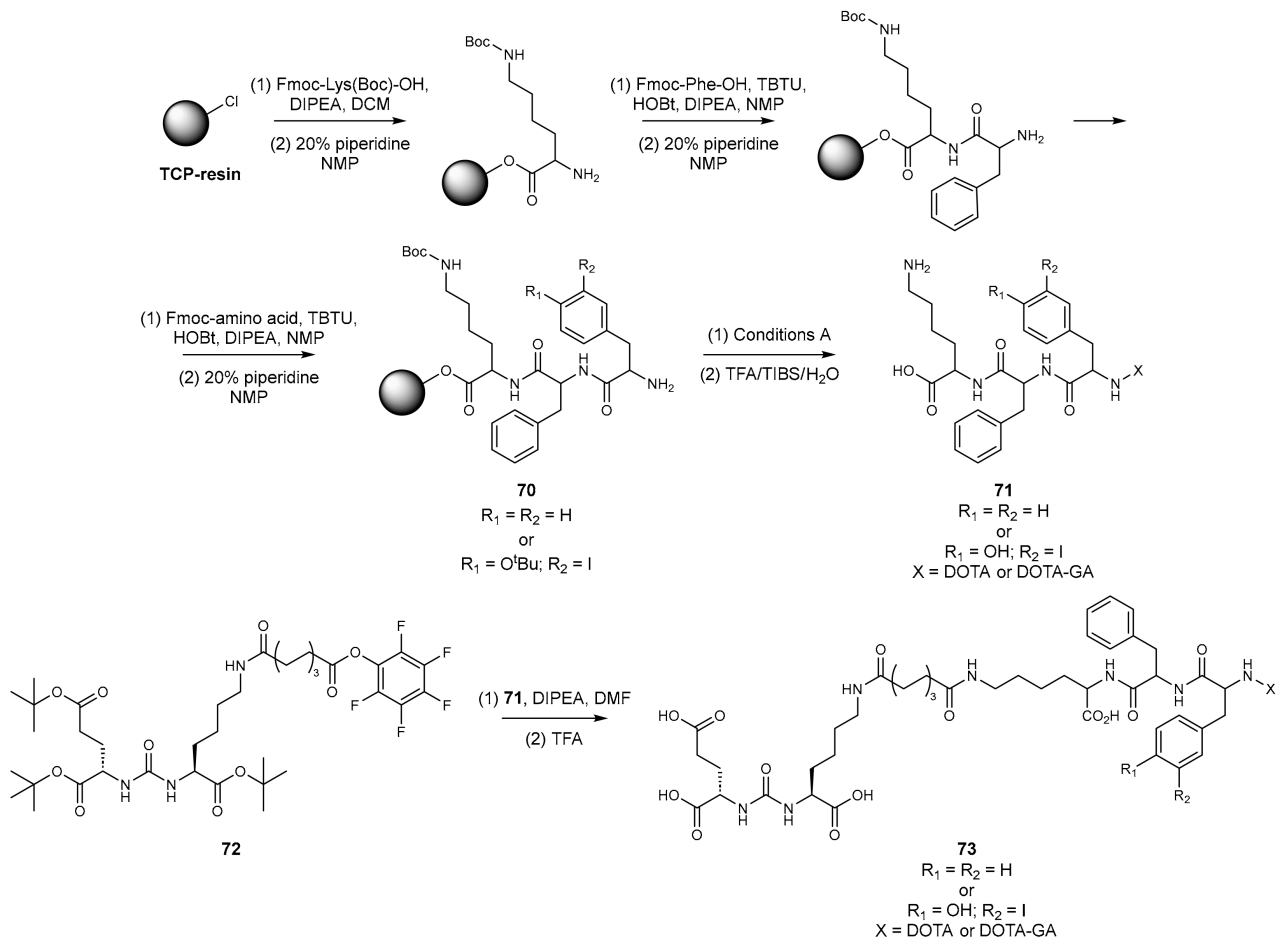
General scheme for the production of PSMA-I&T conjugate and its analogs. Condition A for X = DOTA: DOTA-tri-*tert*-butyl ester; Condition A for X = DOTA-GA: DOTA-GA-anhydride, DIPEA, NMP.

An important representative of such compounds is the conjugate PSMA-I&T and its analogs [59,114]. The scheme for obtaining these compounds involves the synthesis of a tripeptide fragment of a linker on a solid-phase carrier, followed by its modification with a chelating agent (Scheme 38). Trityl chloride-polystyrene resin (TCP resin) was chosen as the solid-phase carrier. In the first stage, lysine, orthogonally protected by Fmoc and Boc protective groups for α- and ε-amino groups, respectively, was immobilized on resin. The subsequent removal of the Fmoc protective group was carried out with a solution of piperidine in *N*-methylpyrrolidone. The peptide sequence was then expanded on the resin to produce tripeptides, **70**, which were acylated with chelating agents, after which the resulting compounds were removed from the solid-phase carrier while acid-labile protective groups were removed using a mixture of trifluoroacetic acid, tri-*iso*-butylsilane and water. The synthesized derivatives, **71**, were acylated with activated pentafluorophenyl ester **72** (the synthesis of this compound is similar to the synthesis of the previously mentioned derivative **52**, Scheme 33), after which the protectinge groups were removed to give the target conjugates, **73**. A similar approach was implemented in the preparation of the PSMA-I&S conjugate containing naphthylalanine and tyrosine residues in the linker [115].

**Scheme 39:**
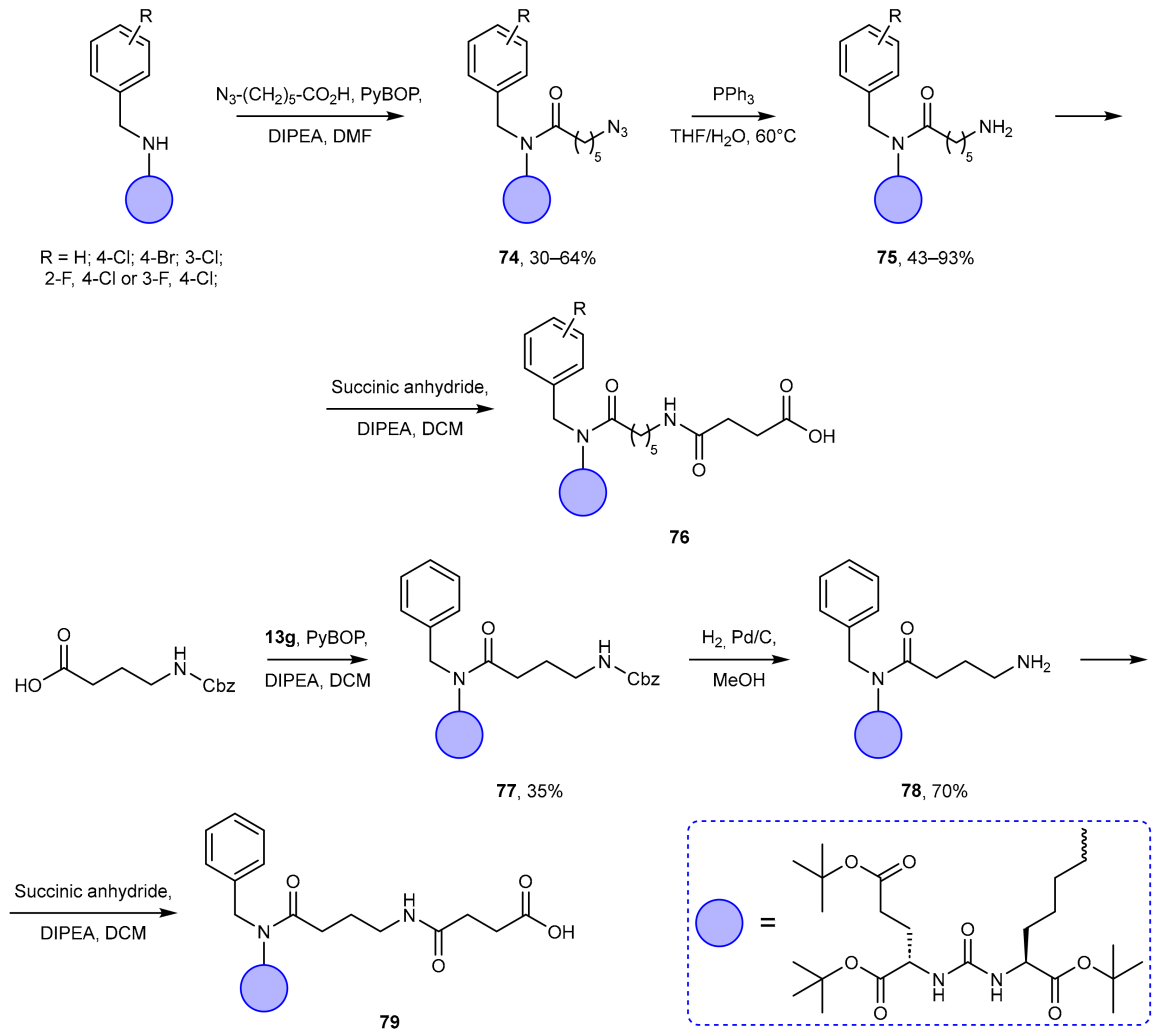
Synthesis of precursor compounds of PSMA ligands **76** and **79**.

An analogous approach was also used in [43]. The authors synthesized a series of modified urea containing substituted benzyl fragments, after which they introduced the resulting secondary amines into an acylation reaction with 6-azidohexanoic acid in the presence of PyBOP to obtain compound **74** (Scheme 39). At the next stage, the terminal azide group was reduced in a Staudinger reaction to obtain amines, **75**, which were then acylated with succinic anhydride to obtain a series of carboxylic acids, **76**. Also, one example of a ligand with a γ-aminobutyric acid residue in a linker was synthesized. In the first stage, the secondary amine **13g** was acylated with Cbz-protected γ-aminobutyric acid. The derivative **77** obtained in this way was subjected to hydrogenolysis to remove the protective group, and amine **78** was acylated with succinic anhydride to form compound **79**.

**Scheme 40:**
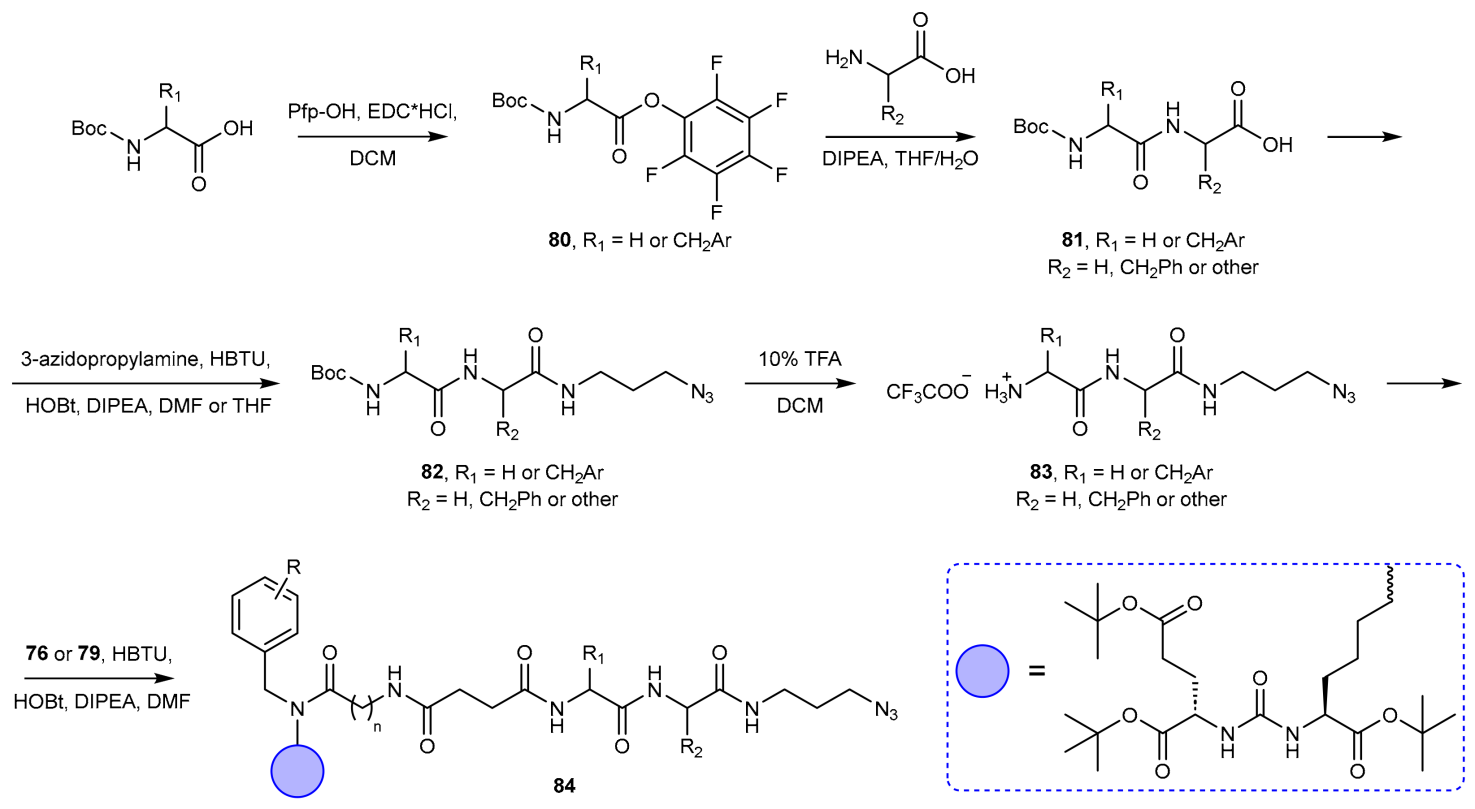
A general scheme for the production of dipeptide fragments and protected ligands using peptide synthesis in solution.

Modified dipeptide fragments were obtained separately using a liquid-phase peptide synthesis strategy (Scheme 40). The first stage included the production of activated pentafluorophenyl esters, **80**, based on the corresponding Boc-protected amino acids. The derivatives obtained in this way were acylated with various amino acids. The synthesized dipeptides, **81**, were then acylated with 3-azidopropylamine to be introduced into the structure of the azide group for subsequent conjugation with diagnostic and therapeutic agents. In the last stage, protective groups were removed from derivatives **82** to release the corresponding trifluoroacetates **83**, which were then reacted with derivatives **76** and **79** (Scheme 39) to obtain protected forms of PSMA ligands, **84**. This strategy was also used in [87,116,117] to obtain similar ligands.

**Scheme 41:**
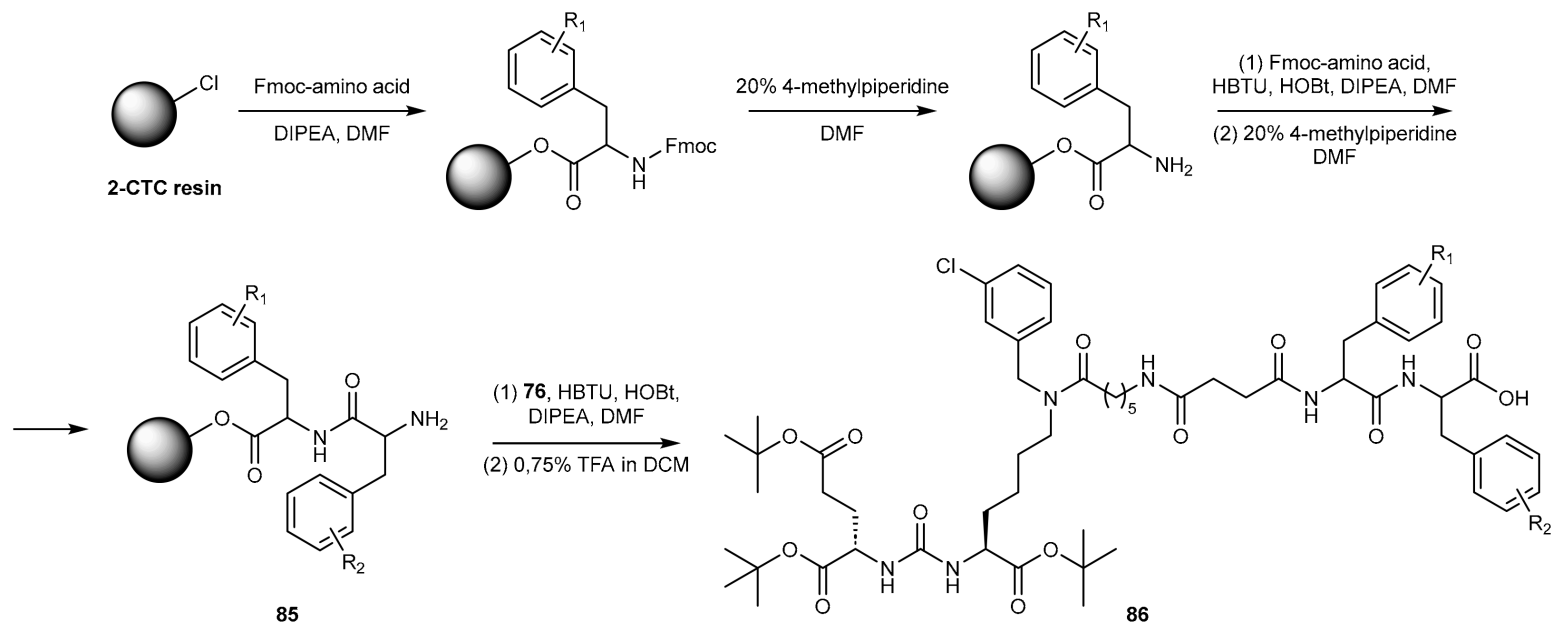
A general scheme for obtaining protected forms of PSMA ligands with a peptide fragment in a linker on a 2-CTC resin.

In [116], the synthesis of similar ligands using solid-phase peptide synthesis was proposed. The method (Scheme 41) assumed, in the first stage, the immobilization of an amino acid on a solid-phase carrier. 2-Chlorotrityl chloride resin (2-CTC resin) was chosen because it is convenient to synthesize using the Fmoc strategy and it is also possible to remove the obtained peptide fragments from the resin without affecting acid-labile protective groups (for example, Boc). After immobilization of the Fmoc-protected amino acid on the resin, the protective group was removed using a 4-methylpiperidine solution, followed by an acylation reaction with the second amino acid in the presence of HBTU, HOBt and DIPEA, and then the Fmoc group was removed. The resulting dipeptide **85** was then acylated with the vector fragment **76**, after which it was removed from the resin. In this way, precursors of the ligand **86** were obtained and then subsequently introduced into the acylation with 3-azidopropylamine. The described method can also be used to synthesize PSMA ligands suitable for the creation of bimodal conjugates with lysine residue in the linker [118,119,120,121].

**Scheme 42:**
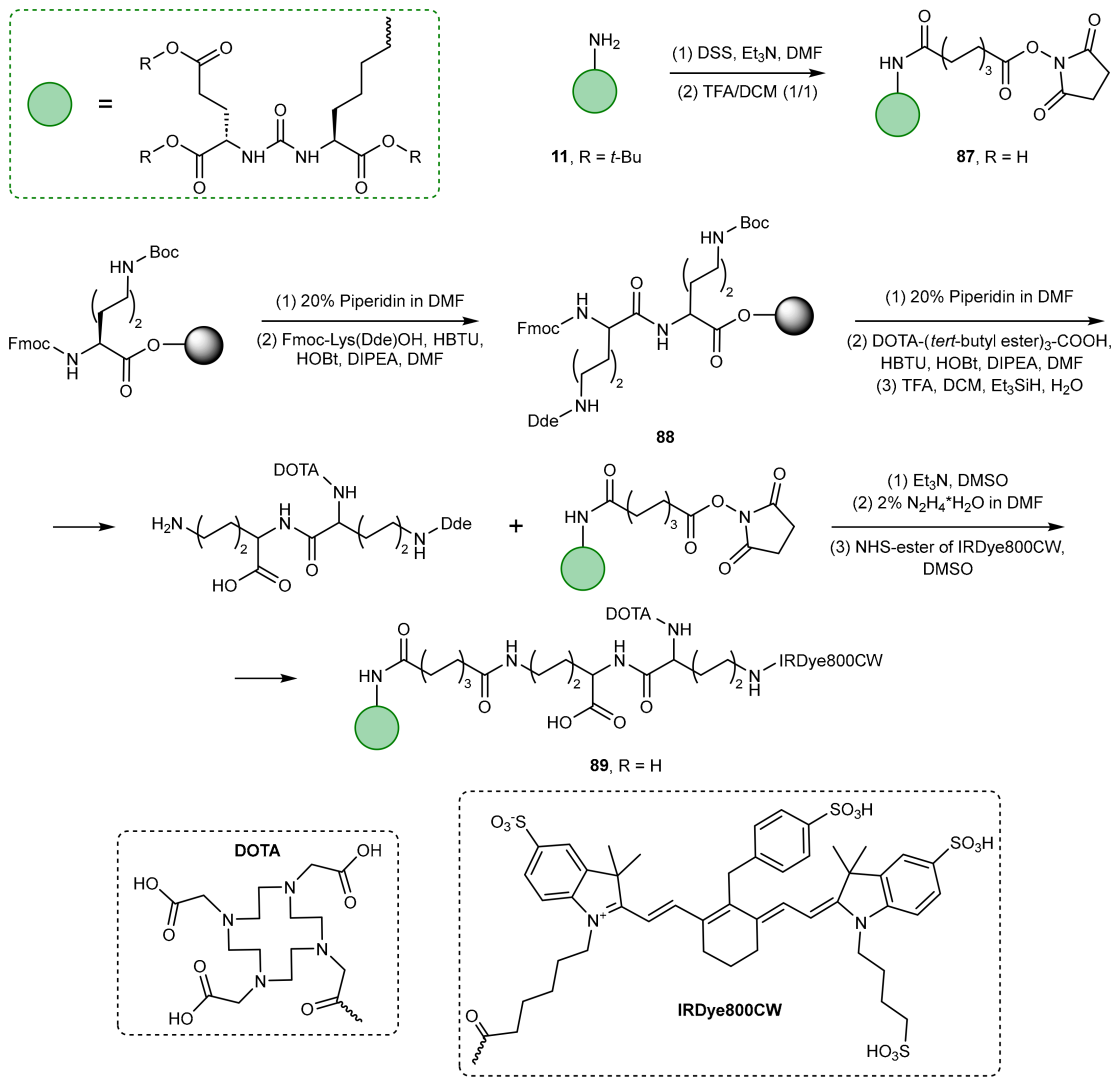
Synthesis of bimodal conjugate **89**.

The approach using a combination of syntheses in solution and on a solid-phase carrier makes it possible to synthesize, among other things, PSMA-directed compounds capable of carrying a double load. Thus, in [122], a scheme for obtaining a bimodal conjugate was proposed (Scheme 42).

Synthetical blocks containing a vector fragment and a chelating agent were obtained separately, followed by conjugation and modification in the last stage with a fluorescent label. To synthesize the vector-containing fragment, urea **11** was reacted with disuccinimidyl suberate (DSS), after which *tert*-butyl protective groups were removed to form compound **87**. The creation of a block containing a chelating agent included modification of lysine Boc-protected by the terminal amino group using a solid-phase peptide synthesis strategy. In the first stage, the authors removed the Fmoc protection from the α-amino group, then reacted with α-Fmoc-protected, ε-Dde-protected lysine in the presence of HBTU, HOBt and DIPEA, after which the Fmoc protection group was removed and reaction with the tris-*tert*-butyl ester chelating agent DOTA was performed. The resulting compound **88** was removed from the resin with simultaneous removal of acid-labile protective groups. These two fragments were then introduced into the acylation reaction in the presence of a base. Subsequently, the Dde group was removed and the resulting substance was reacted with NHS-ester IRDye800CW to produce bimodal conjugate **89**.

In summary, complex linker synthesis employs peptide methods (solid- and liquid-phase). Convergent approaches—pre-synthesizing the linker (or its fragment) before conjugation—are preferred over sequential DCL modification, improving overall yields.

In addition to influencing inhibitory activity, the linker has a significant effect on other properties of the final ligands and conjugates based on them, for example, on the water solubility and lipophilicity of the resulting compounds. In this regard, a PEG fragment is often introduced into the linker structure. The most common approach to obtaining such compounds involves acylation of the ε-amino group of lysine with PEG-containing carboxylic acid.

**Scheme 43:**
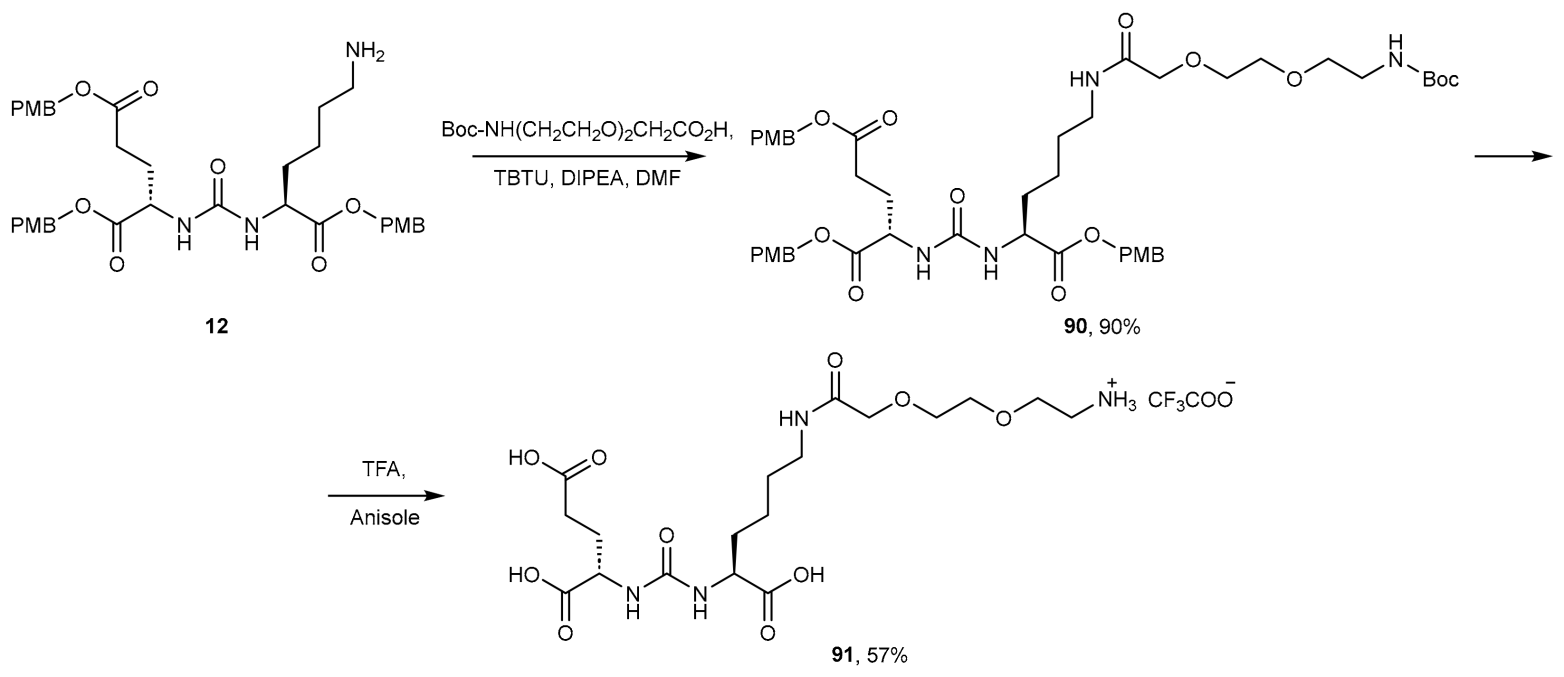
Scheme of synthesis of ligand **91** with a diethylene glycol fragment in a linker.

In [73], compound **12** was acylated with carboxylic acid with a terminal Boc-protected amino group and two ethylene glycol residues in the structure (Scheme 43). TBTU was used as an activator, and DIPEA was used as a base. The derivative **90** obtained in this way was subjected to the removal of acid-labile protective groups to obtain ligand **91**.

A similar scheme for obtaining PSMA-directed compounds containing a PEG fragment was realized in [85]. Compounds containing biotin and PEG fragments with a different number of units in the linker were synthesized according Scheme 43. In the described work, two different synthetic schemes were implemented. For the compound with eight ethylene glycol units in the linker, the modification of compound **11** was carried out in three stages. In the first stage, the ε-amino group of lysine was acylated with an acid containing a PEG fragment and a terminal amino group protected by a Cbz group in the presence of TBTU and DIPEA. The Cbz group was then removed by hydrogenolysis and acylated with biotin NHS-ester to form compound **92a**. In the case of a longer linker, the authors introduced into the acylation reaction a PEG-containing acid modified at the N-terminus by a biotin residue under similar conditions to obtain compound **92b**. In both cases, acid-labile protective groups were removed in trifluoroacetic acid in the last stage to obtain compounds **93a**,**b**.

**Scheme 44:**
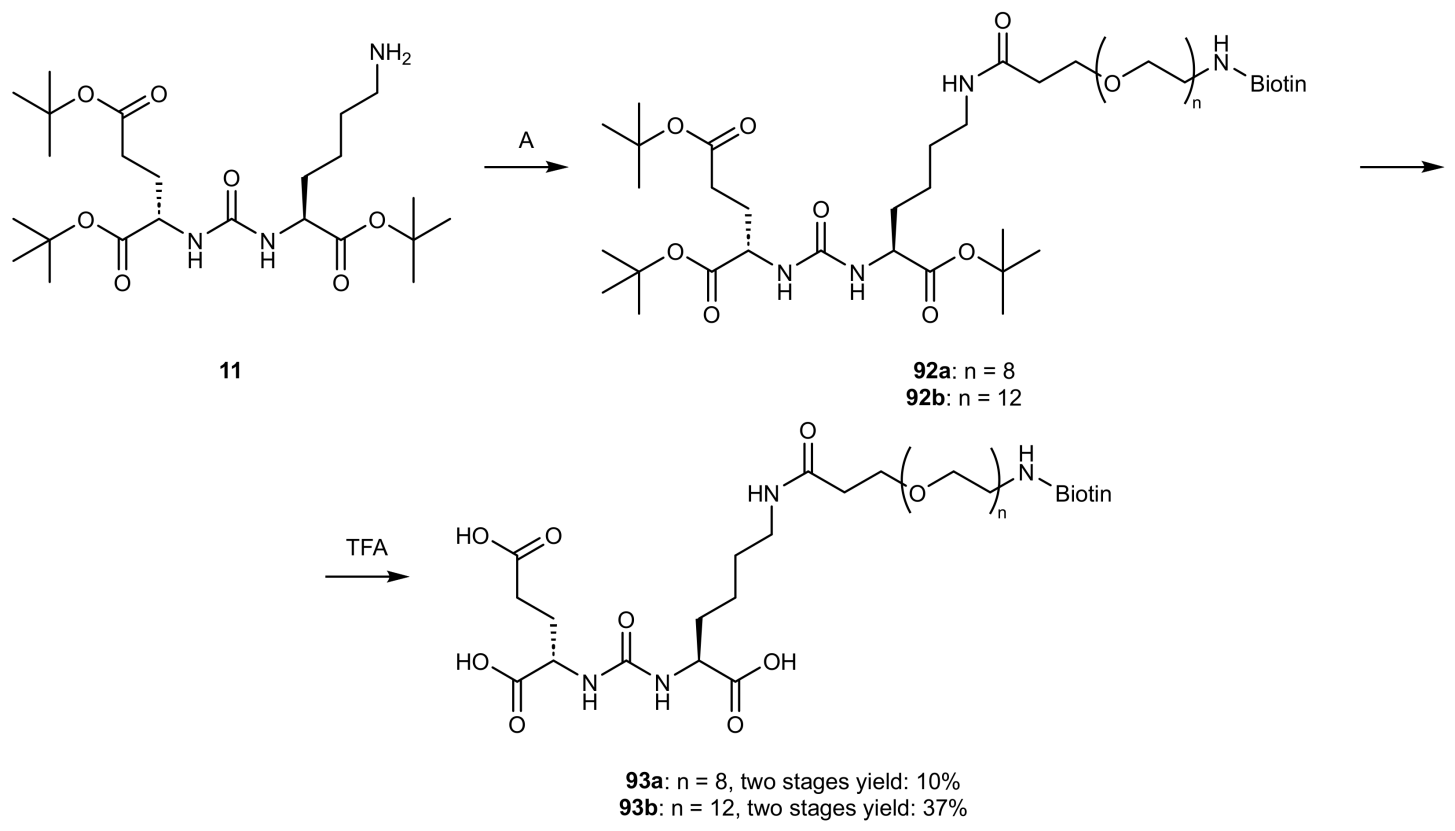
Synthesis of PSMA-targeted compounds containing a biotin fragment. Condition A for n = 8: (1) Z-NH-PEG_8_-COOH, TBTU, DIPEA, DMF; (2) H_2_, Pd(OH)_2_, MeOH; (3) NHS-Biotin, DIPEA, DMF. Condition A for n = 12: HOOC-PEG_12_-Biotin, TBTU, DIPEA, DMF.

The methods presented in Scheme 43 and Scheme 44 involve the use of the acylation reaction of the ε-amino group of lysine of DCL urea to produce PEG-containing PSMA ligands. This synthetic scheme has become the most widespread, while a fairly extensive set of methods has been proposed in the literature.

**Scheme 45:**
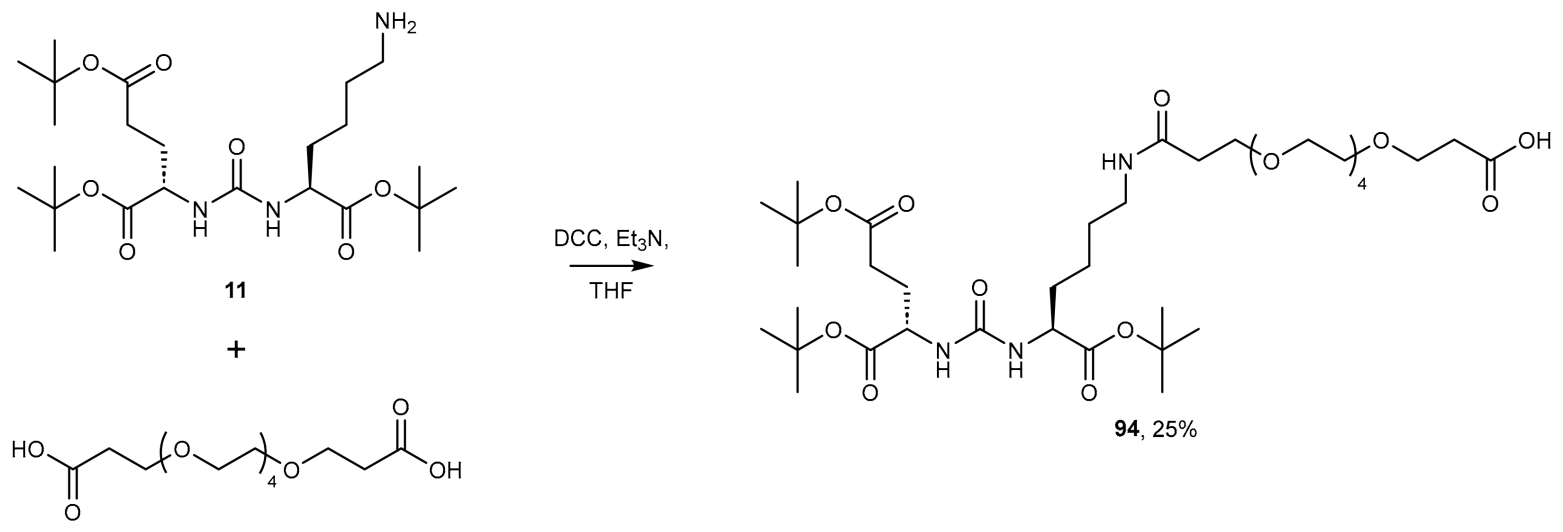
Introduction of a PEG linker using DCC.

Thus, in [123], the authors introduce a residue of PEG-containing dicarboxylic acid into the structure of the PSMA ligand due to the acylation reaction (Scheme 45). Carbodiimide DCC was used as the activator and the yield of the target compound **94** was 25%. The rather moderate yield of this reaction can be justified by the occurrence of a side process of acylation by compound **94** of the second equivalent of protected urea **11**.

**Scheme 46:**
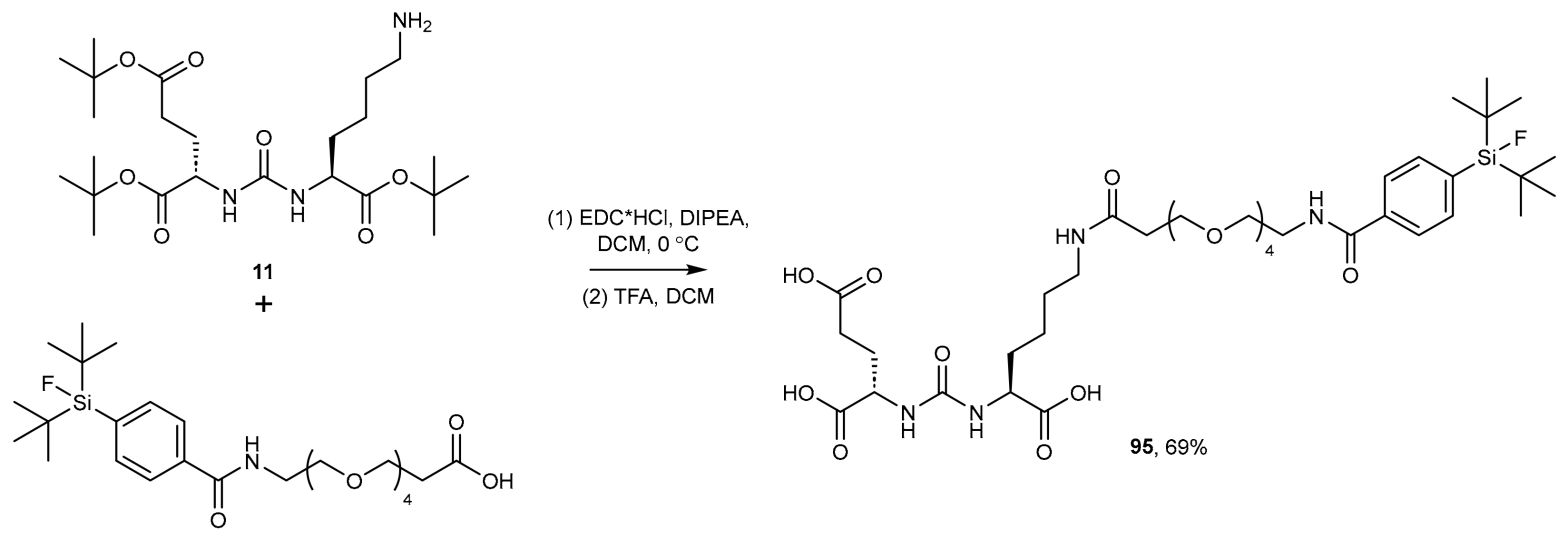
Synthesis of compound **95** using EDC*HCl.

Carbodiimides can also be used to introduce a pre-modified PEG linker. In the article [68], the authors acylated the amino group of urea **11** with a PEG-containing ω-amino acid, the terminal amino group of which was modified with an SiFA—organosilicon-[^18^F]fluoride acceptor (Scheme 46). EDC hydrochloride was used as the coupling agent, and the reaction was carried out in the presence of DIPEA. Subsequently, acid-labile protective groups were removed and the total yield of compound **95** was 69%.

**Scheme 47:**
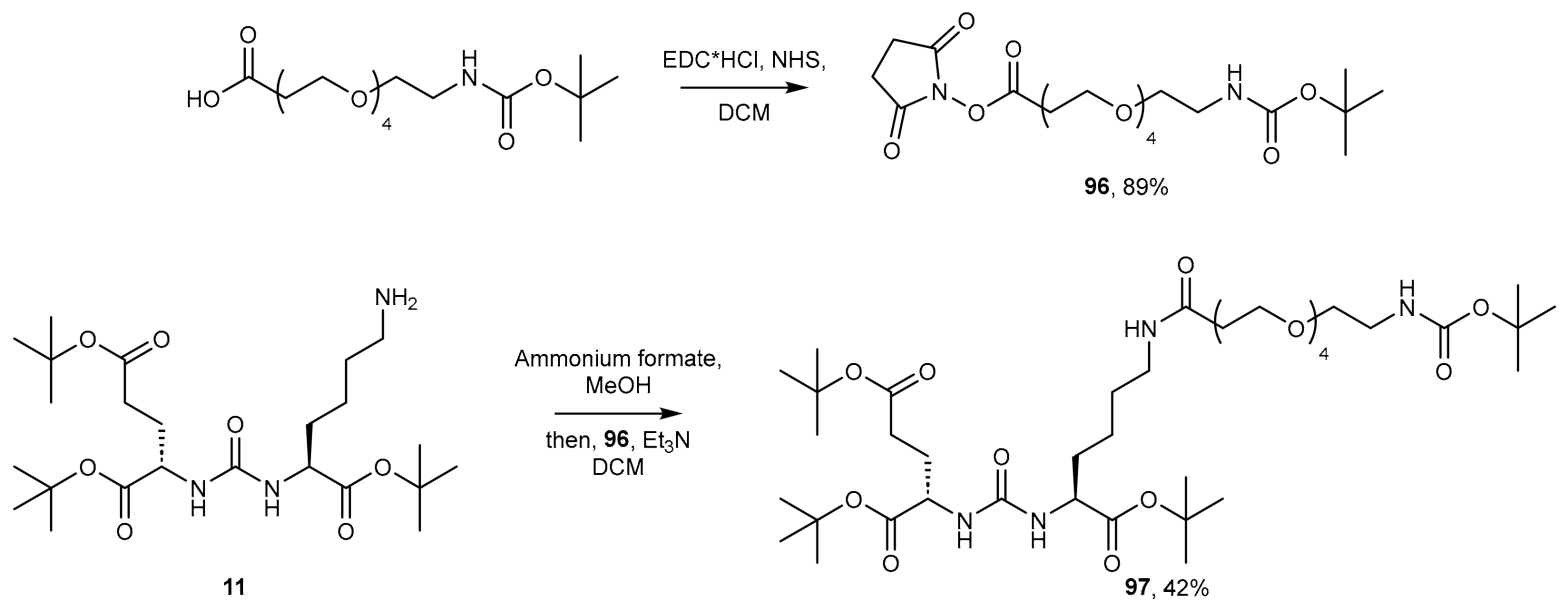
Synthesis of a protected PEG-containing ligand **97**.

It should be noted that the use of EDC to generate NHS esters from PEG-containing carboxylic acids has become increasingly common. In [50], the authors prepared NHS-ester **96** in the presence of EDC*HCl in the first stage and introduced it into the acylation reaction with compound **11** (Scheme 47). The resulting derivative **97** contained a Boc-protected terminal amino group, which could then be easily modified.

**Scheme 48:**
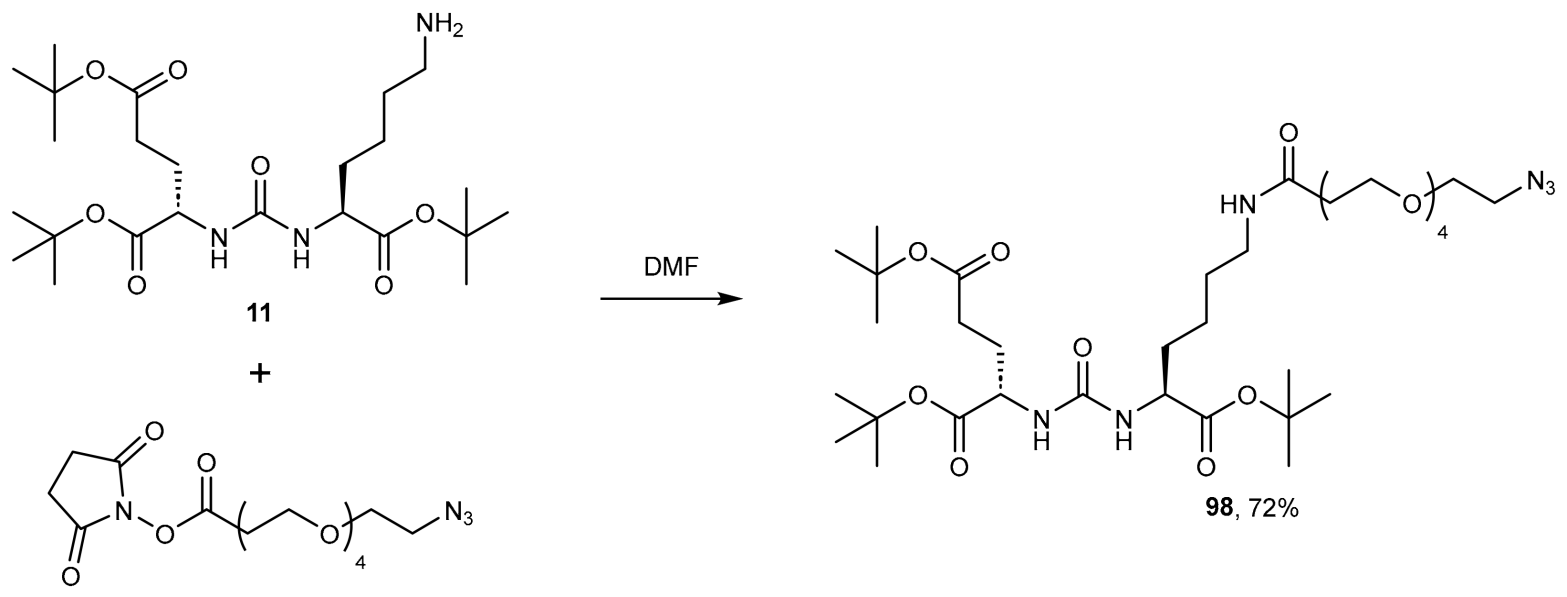
Synthesis of the azide-containing PSMA ligand **98**.

The NHS-ester of azido-containing PEG carboxylic acid was used to obtain compound **98** (Scheme 48), the synthesis of which is presented in [124]. The presence of an azide group in this position allowed further conjugation of the ligand with an alkyne containing an albumin-binding fragment and a chelator.

**Scheme 49:**
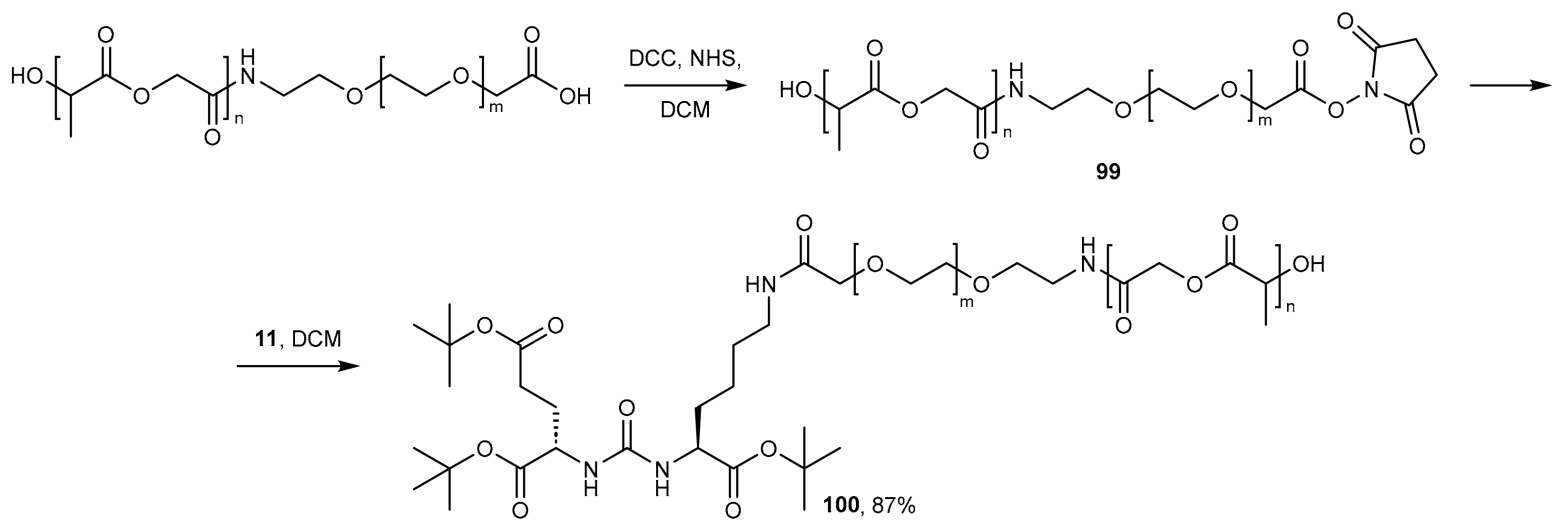
Synthesis of the PSMA ligand modified with a PLGA-PEG block co-polymer.

NHS-esters are also used to introduce PEG-containing co-polymers into the structure of PSMA ligands. For example, in [125], the authors synthesized NHS-ester **99** with a PLGA-PEG block co-polymer, which was reacted with protected urea **11** to produce compound **100** (Scheme 49). In this case, DCC was used to obtain NHS-ester, not EDC.

**Scheme 50:**
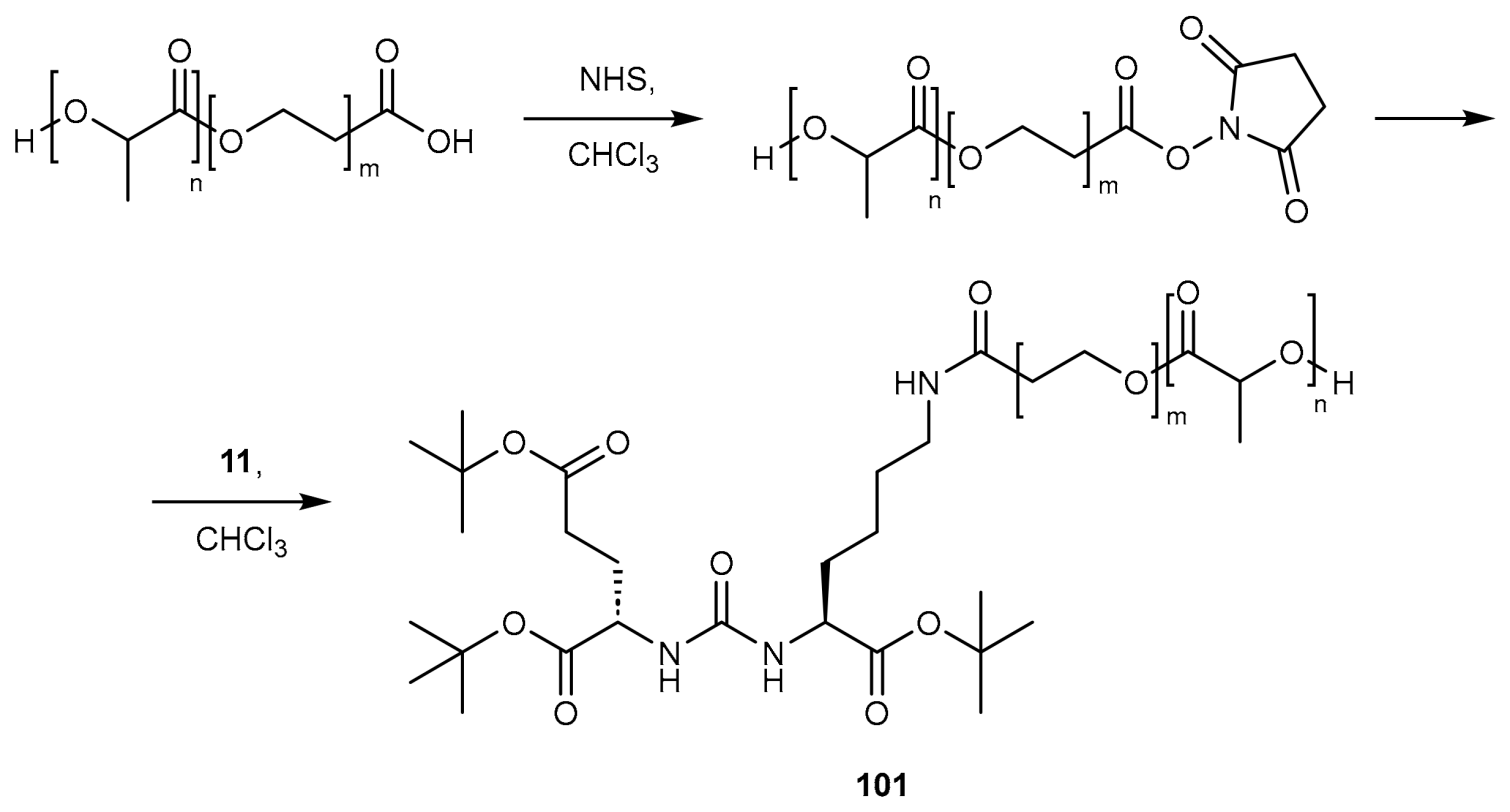
Synthesis of compound **101**.

Another similar example is presented in [126]. The authors give an extremely terse description of the synthesis of compound **101** (Scheme 50). According to the methodology described in the article, carboxylic acid, *N*-hydroxysuccinimide and compound **11** were mixed in chloroform, which led to the formation of the target product containing a co-polymer of polyethylene glycol and poly-*d*,*l*-lactide. The presented technique raises significant questions regarding its reproducibility, since the formation of NHS-ester without the participation of an activator (for example, EDC) seems unlikely.

**Scheme 51:**
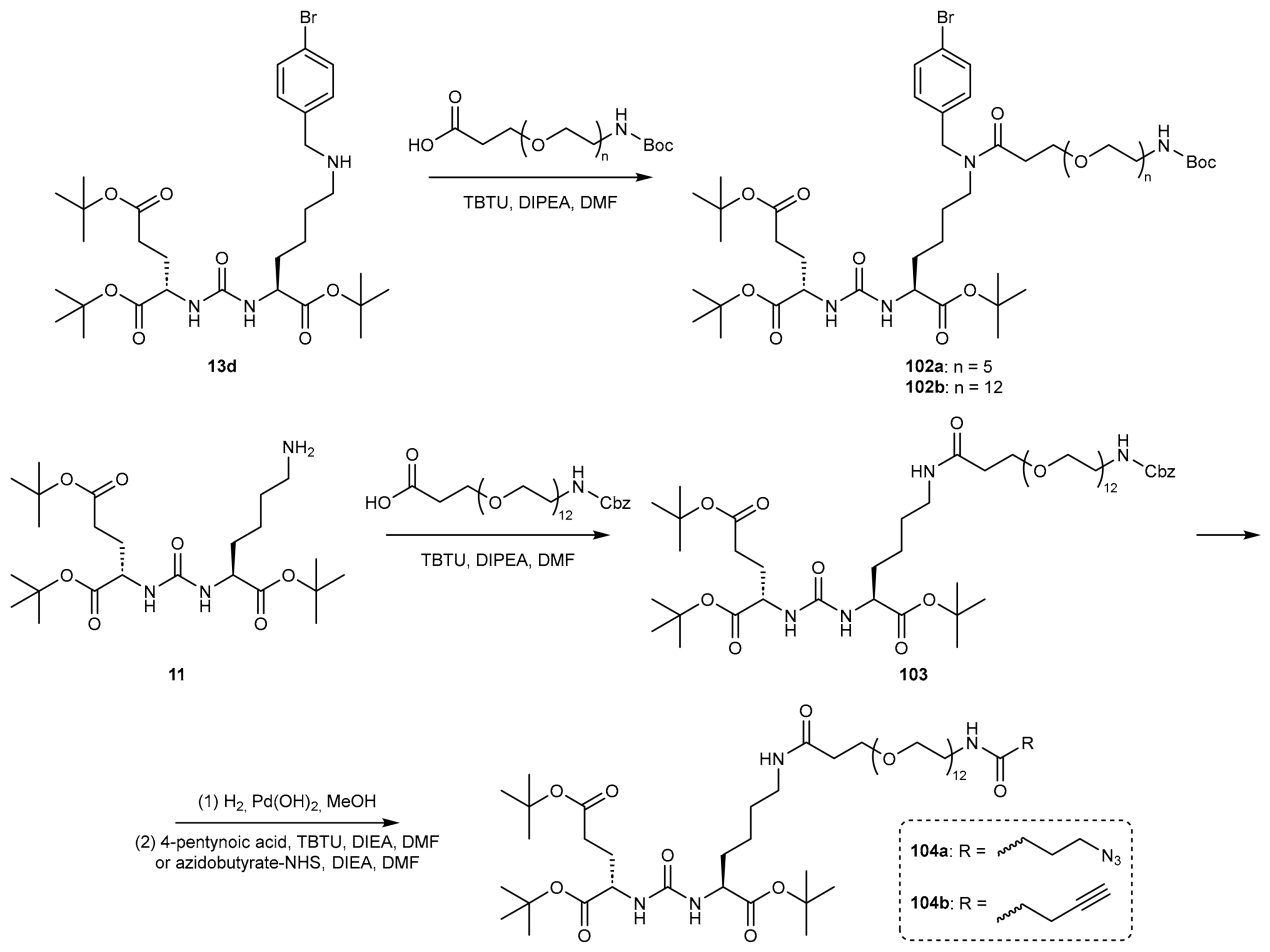
Approaches to the introduction of a linker into the structure of the PSMA ligand by acylation in the presence of TBTU.

A common alternative to the use of carbodiimides and NHS-esters is the use of uronium and phosphonium salts such as HATU, TBTU and PyBOP. Thus, in [86,127], an approach to the acylation of protected urea **11** and derivative **13d** with PEG-containing carboxylic acids was proposed (Scheme 51). The introduced linker fragments also contained a Boc- or Cbz-protected amino group, and the acylation reaction was performed in the presence of TBTU. Thus, derivatives **102a**,**b** and **103** were obtained. In the article [127], methods for further modification of compound **103** were proposed by selectively removing the Cbz protective group and subsequent acylation to introduce a terminal azide group (compound **104a**) or a triple bond (compound **104b**) into the ligand structure.

**Scheme 52:**
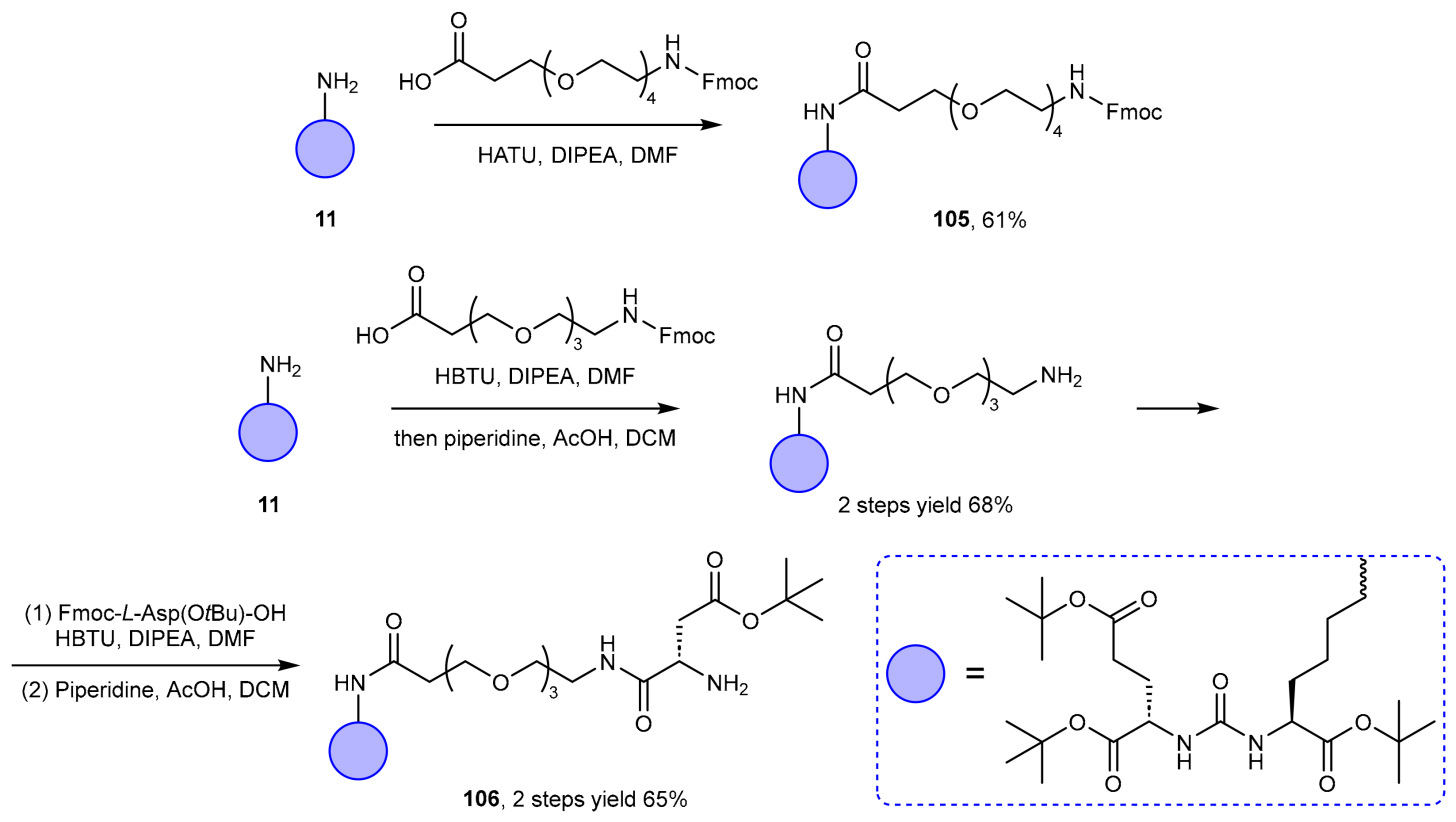
Introduction of an Fmoc-protected PEG-containing linker.

In [40], a method for the synthesis of compound **105** (Scheme 52) was proposed. A carboxylic acid with an Fmoc-protected terminal amino group was selected as a PEG linker. The reaction was carried out in the presence of HATU and the product yield was 61%. The use of the Fmoc group allows for further selective removal and subsequent modification of this provision. A similar strategy was applied in the already mentioned work [68]. *N*-Fmoc-protected acid was also introduced there as a linker, after which selective removal of protection and subsequent acylation with aspartic acid were carried out to obtain compound **106**. HBTU was used as a coupling agent in both stages of acylation.

**Scheme 53:**
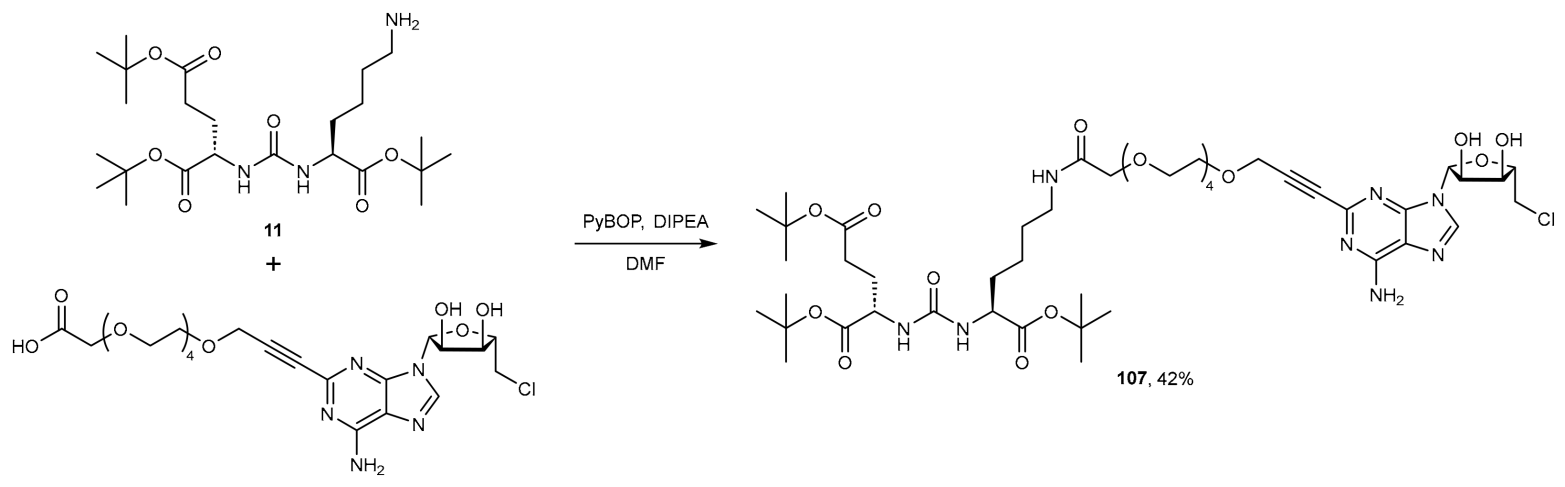
Synthesis of a PSMA-targeted compound containing a fragment of C-2 modified 5′-chloro-5′-deoxyadenosine.

In [51], the authors acylated protected urea DCL in the presence of PyBOP with a pre-modified linker containing C-2 modified 5′-chloro-5′-deoxyadenosine (Scheme 53). Compound **107** was isolated with a yield of 42%, which is lower than at the HBTU or HATU using, which, however, may be caused by a slightly more difficult purification.

**Scheme 54:**
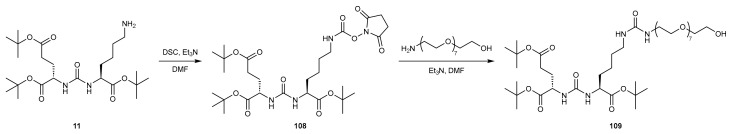
Introduction of a PEG linker by creating a urea fragment.

In addition to the acylation of the ε-amino group of lysine, there are other options for the introduction of a PEG-containing linker into a ligand structure. However, they have become less widespread. Thus, in the article [64], the authors introduced a linker by creating a urea fragment (Scheme 54). Protected urea DCL was introduced into the carbamate **108** formation reaction under the action of DSC, after which urea **109** was obtained.

**Scheme 55:**
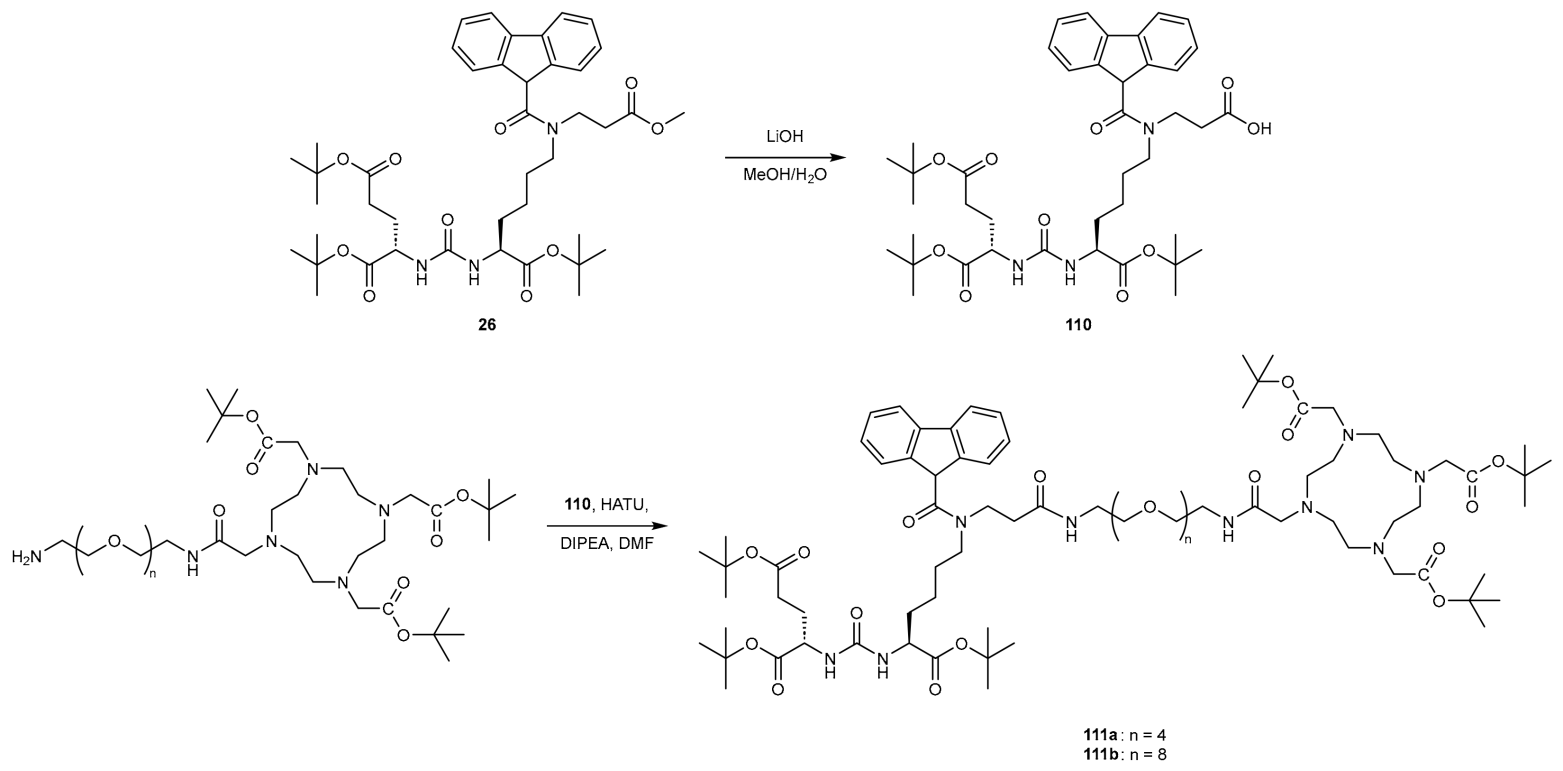
Scheme of synthesis of PEG-containing protected conjugates **111a**,**b**.

An alternative option for the introduction of a PEG-containing linker was proposed in the previously mentioned work on the preparation of a PSMA-directed compound with a 9-carboxyfluorene residue [94]. Compound **26** obtained according to Scheme 15 was subjected to alkaline hydrolysis to obtain carboxylic acid **110**, which was introduced into an acylation reaction with the protected chelating agent DOTA modified with PEG-containing linkers of different lengths (Scheme 55). HATU was chosen as the activating agent, and DIPEA was chosen as the base. Thus, two protected conjugates **111a**,**b** were obtained.

As discussed earlier, as linker classes, PEG-containing fragments are primarily introduced via DCL lysine ε-amino group acylation using carbodiimides, uronium/phosphonium salts, or activated esters. No optimal protocol emerges—conditions must match the substrate. Missing yield data for several substrates further complicates selection of an optimal strategy.

**Scheme 56:**
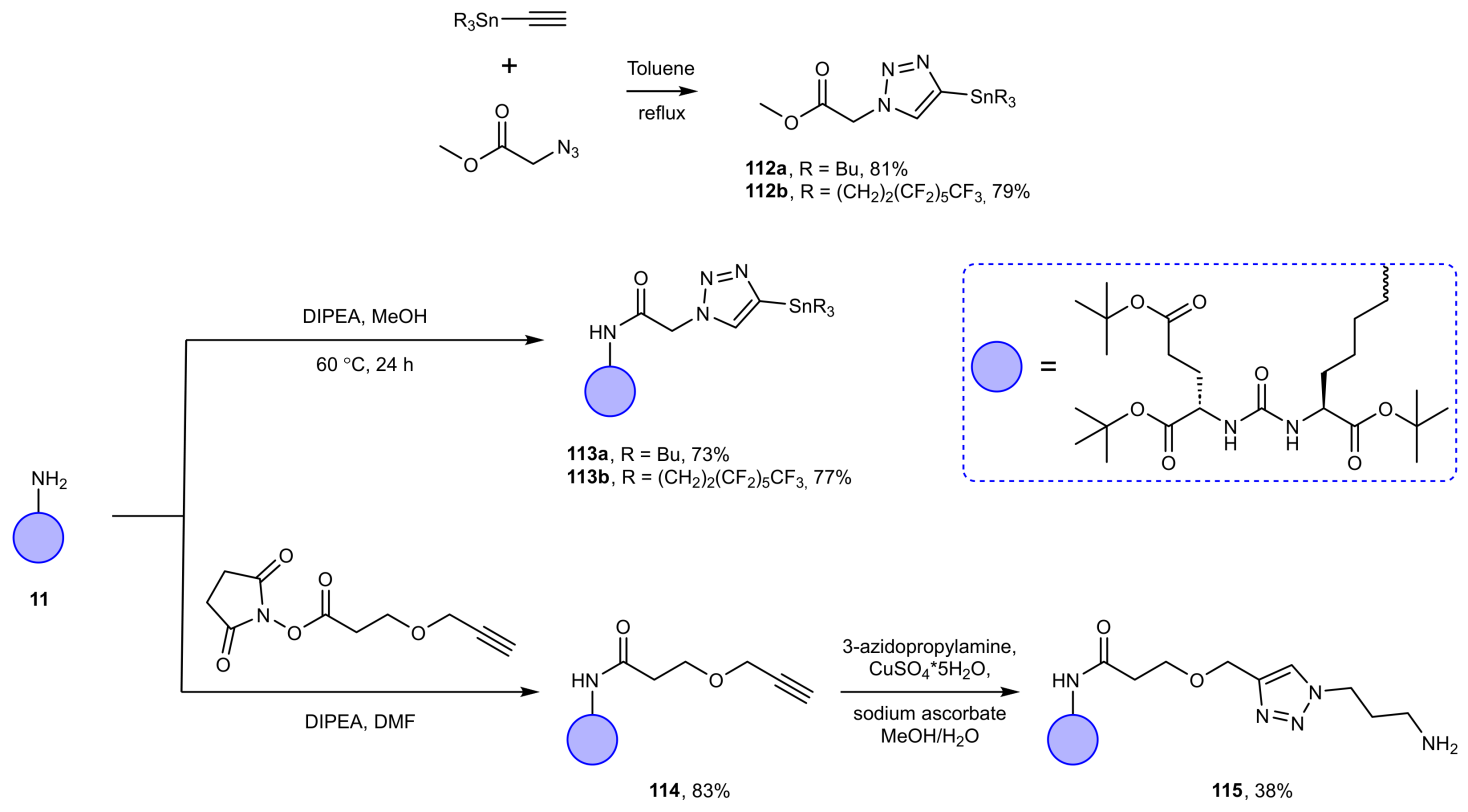
Synthesis of protected PSMA ligands containing a triazole fragment.

One more design option for PSMA ligands assumes the presence of cyclic fragments in the linker structure. In some cases, this fragment is a triazole cycle formed as a result of the CuAAC reaction upon conjugation of a vector molecule with a therapeutic or diagnostic agent. In this review, we will not consider all such examples, since this reaction is often used in the synthesis of PSMA-directed conjugates. However, a number of examples will still be considered. Thus, in [68,128], the authors modified compound **11** by introducing a linker with a triazole cycle (Scheme 56). In the first case, the authors separately synthesized substituted esters of triazolacetic acids **112a**,**b**, which were subsequently introduced into the acylation reaction to obtain compounds **113a**,**b**. The structure of these compounds contains stanyl groups, which can later be easily substituted by ^123^I. In the second example, protected urea DCL was acylated with an alkyne-containing acid. The resulting compound **114** was introduced into the reaction of the [3+2]-azide-alkyne cycloaddition with 3-azidopropylamine. It is worth noting a rather moderate yield of 38%, which is unusual for this reaction. The structure of the protected ligand **115** synthesized in this way contains a terminal amino group, which can be modified both before and after the removal of acid-labile protective groups.

**Scheme 57:**
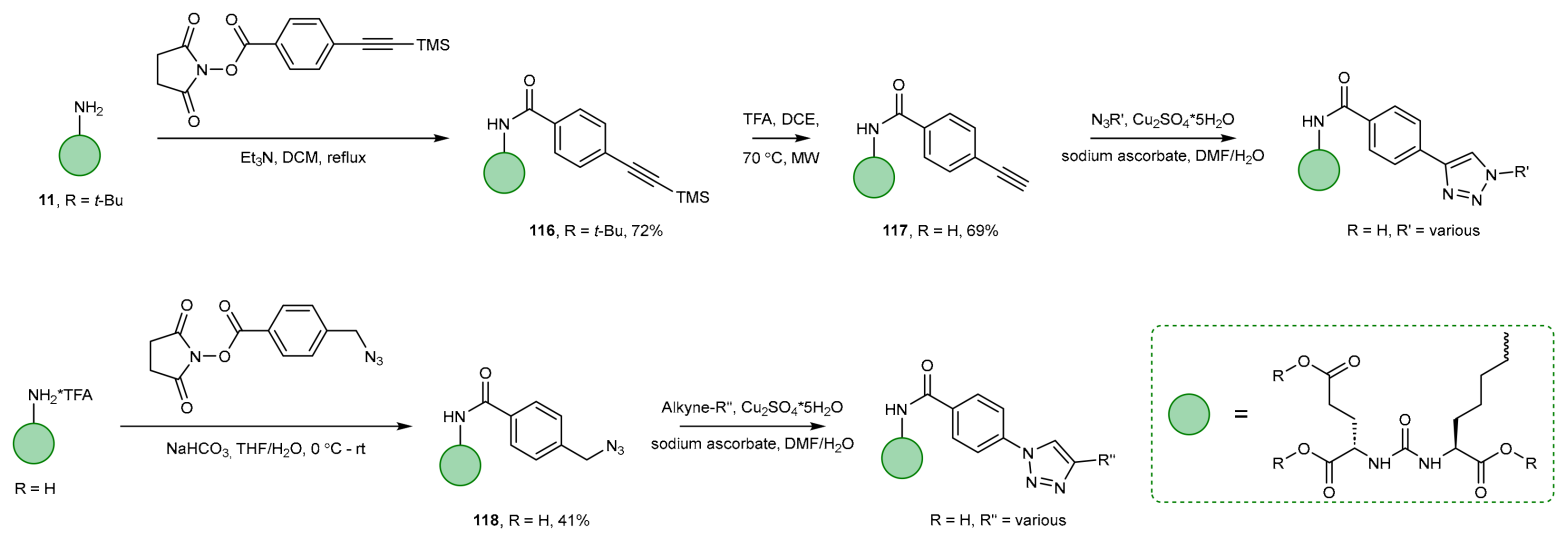
Synthesis of azido- and alkyne-containing ligands **117** and **118**.

The formation of the triazole cycle was also carried out in [42]. In contrast to the previous examples, the authors synthesized a series of compounds containing a residue of substituted benzoic acid in the linker structure (Scheme 57). At the same time, they obtained both a variant of modified DCL urea with an azide group and a triple bond. Different approaches were used. In the case of an alkyne-containing ligand, urea **11** was acylated with NHS-ester of TMS-protected *para*-ethinyl benzoic acid in the first stage. The resulting derivative **116** was then subjected to the removal of protective groups to produce ligand **117**. The resulting ligand was then reacted with various azides. In the case of the azide-containing ligand, urea trifluoroacetate DCL was used as the starting compound. This salt was introduced into the acylation reaction with NHS-ester 4-(azidomethyl)benzoic acid in the presence of a base. The ligand **118** obtained in this way was then introduced into a cycloaddition reaction with alkynes of different structures.

**Scheme 58:**
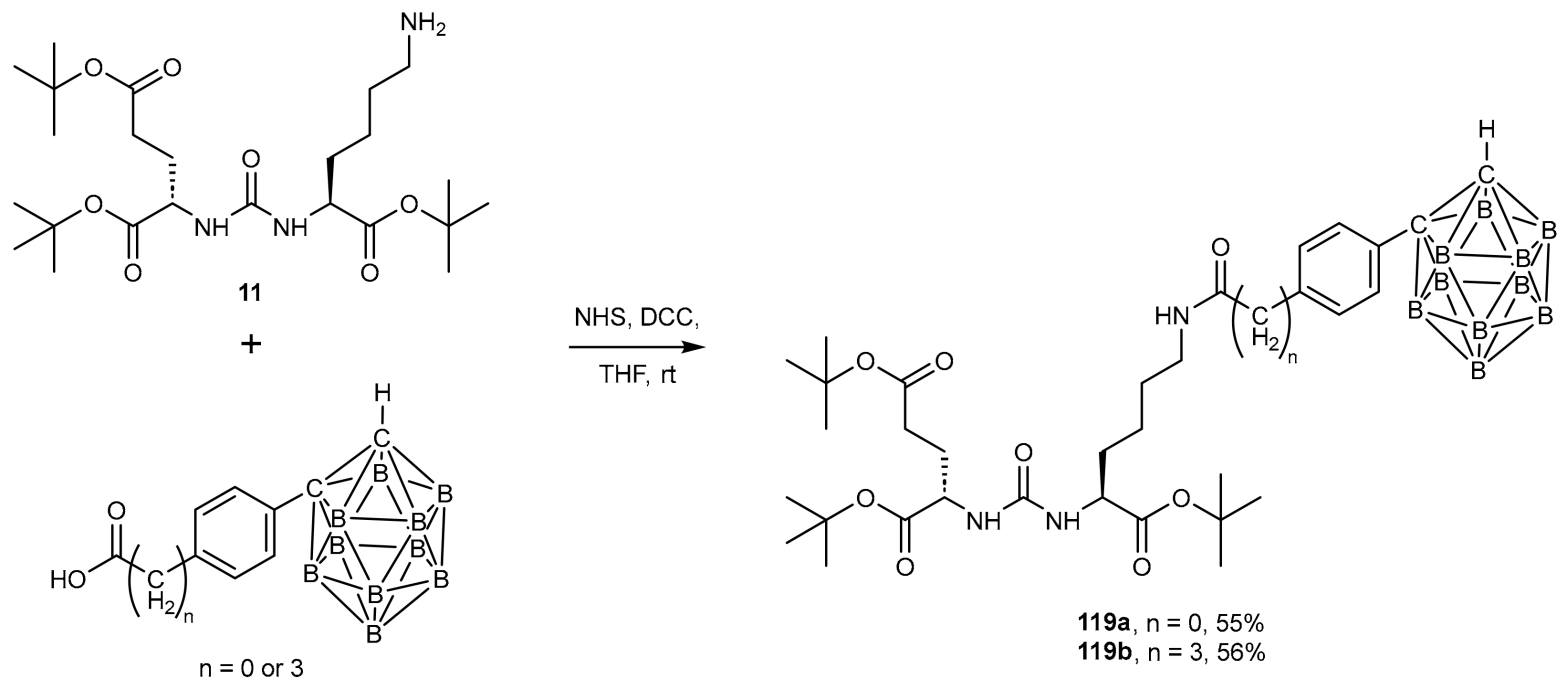
Synthesis of compounds **119a**,**b** containing 1,2-dicarba-*closo*-dodecarboranyl.

Substituted benzoic and other carboxylic acids containing a benzene ring are quite often used as a linker unit. For example, due to the reaction of compound **11** with boron-substituted benzoic acids, derivatives **30b** and **31**, mentioned earlier, were obtained [96]. However, in the same work, compounds **119a**,**b** (Scheme 58) were obtained containing in their structure a 1,2-dicarba-*closo*-dodecarboranyl articulated with benzene-containing carboxylic acids. The reaction was carried out in the presence of DCC and NHS and the yield was 55% for compound **119a** and 58% for compound **119b**.

**Scheme 59:**
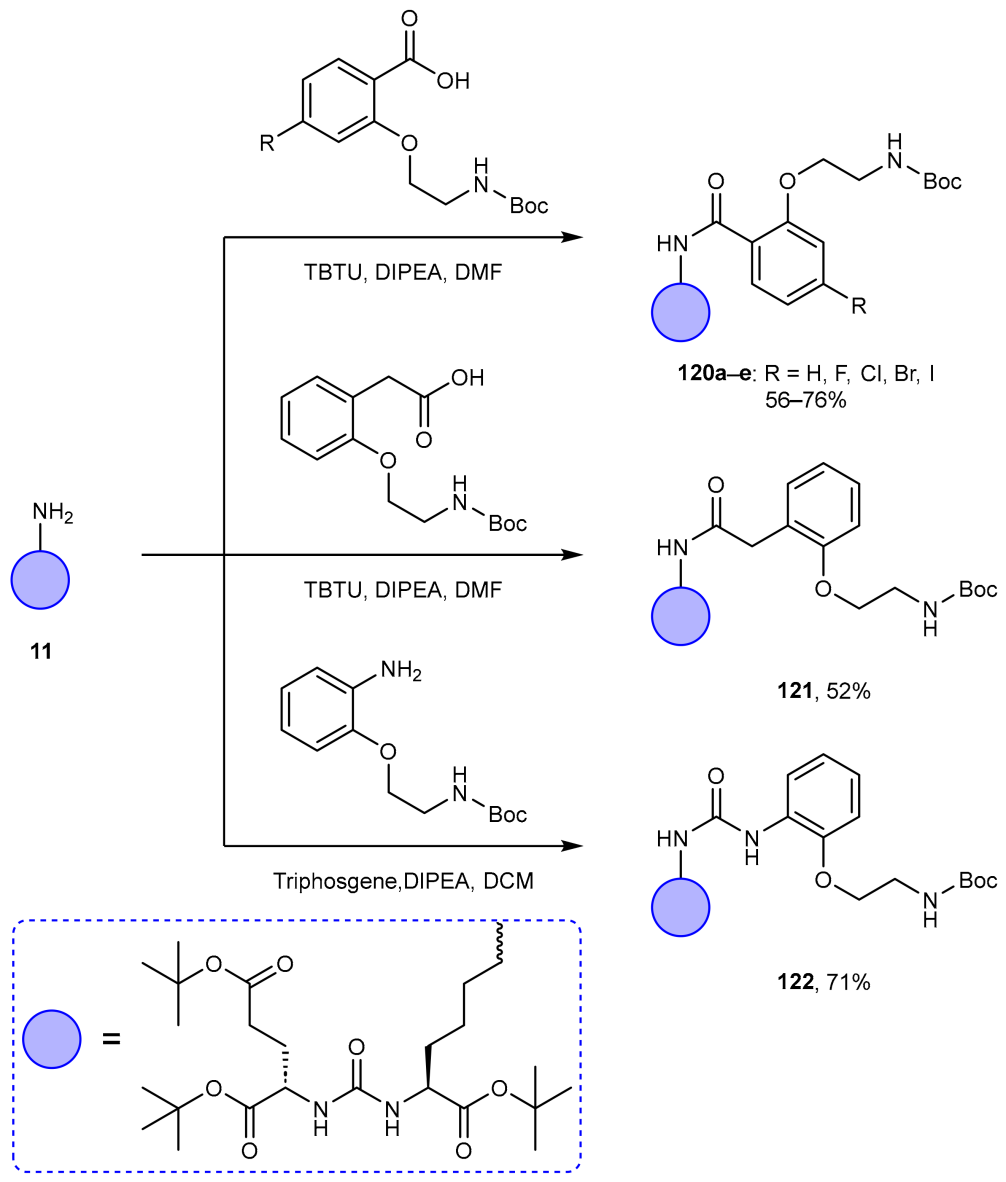
Synthesis of PSMA ligands containing a cyclic fragment, proposed in [85].

Several options for introducing a linker containing a substituted benzene ring into the structure of ligand were presented in Jan Tykvart’s work [85] (Scheme 59). The following variations of the cyclic linker were proposed: based on substituted benzoic acids (**120a**–**e**), based on substituted phenylacetic acid (**121**) and by creating a urea fragment (**122**).

The point of subsequent modification in the case of all compounds is an ethanolamine fragment. Initially, it contained a Boc protective group at the nitrogen atom, which, after being combined with a urea fragment, was removed by 15-min TFA treatment without characterizing these compounds. To obtain ligands by acylation, TBTU was used as an activator and the yield was 56–76% for compounds **120a**–**e** and 52% for compound **121**. The synthesis of compound **122** consisted in treating compound **11** with triphosgene to generate isocyanate in situ, followed by the introduction of substituted aniline into the reaction. The final yield of compound **122** was 71%.

There are also other works where a fragment of a benzene ring is introduced into the linker, for example [65]. In this work, the acylation reaction of the ε-amino group of protected urea DCL was used to introduce a phenoxyacetic acid-based linker coupled with 1,4-naphthalene. The reaction was carried out in the presence of HBTU and DIPEA, after which the protective groups were removed and the subsequent production of 1,4-dimethyl-1,4-endoperoxide directed at PSMA was carried out.

**Scheme 60:**
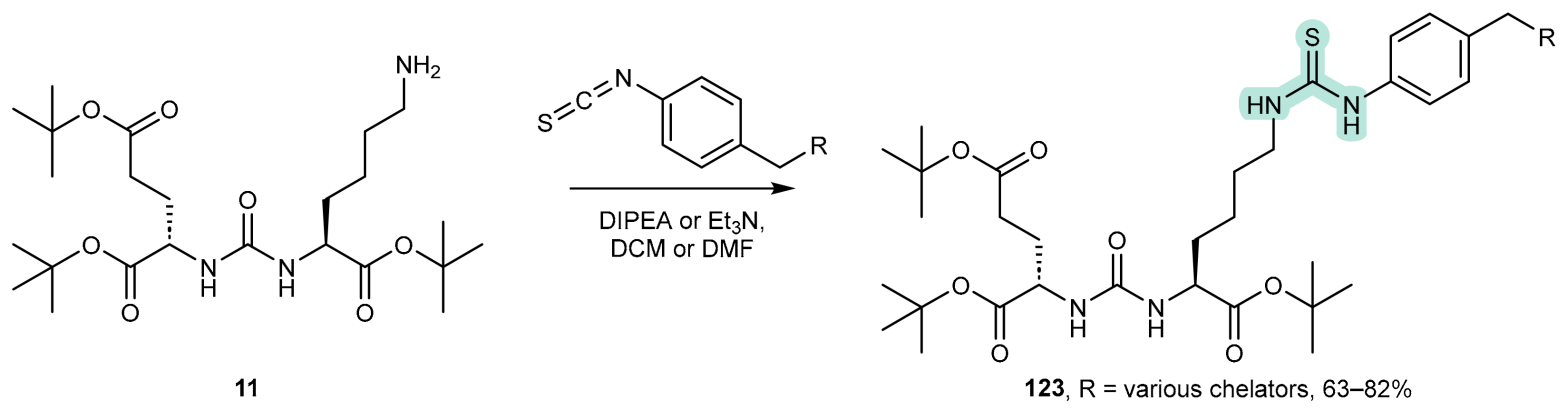
The introduction of an aromatic fragment by creating a thiourea bridge.

There are some examples of works where the aromatic fragment is introduced into the linker by creating a thiourea fragment (Scheme 60) [53,56,60]. In these works, urea **11** was reacted with substituted phenylisothiocyanates to produce thioureas of general structure **123**. The reaction can be carried out in both DMF and DCM. In two of the three cases, the reaction was carried out in the presence of a base (DIPEA or Et_3_N). The reaction of thiourea formation proceeded with rather high yields (63–82%). Various chelating agents, suitable for complexation with Ga, Al and Ti, were presented as substituents in the benzene ring.

**Scheme 61:**
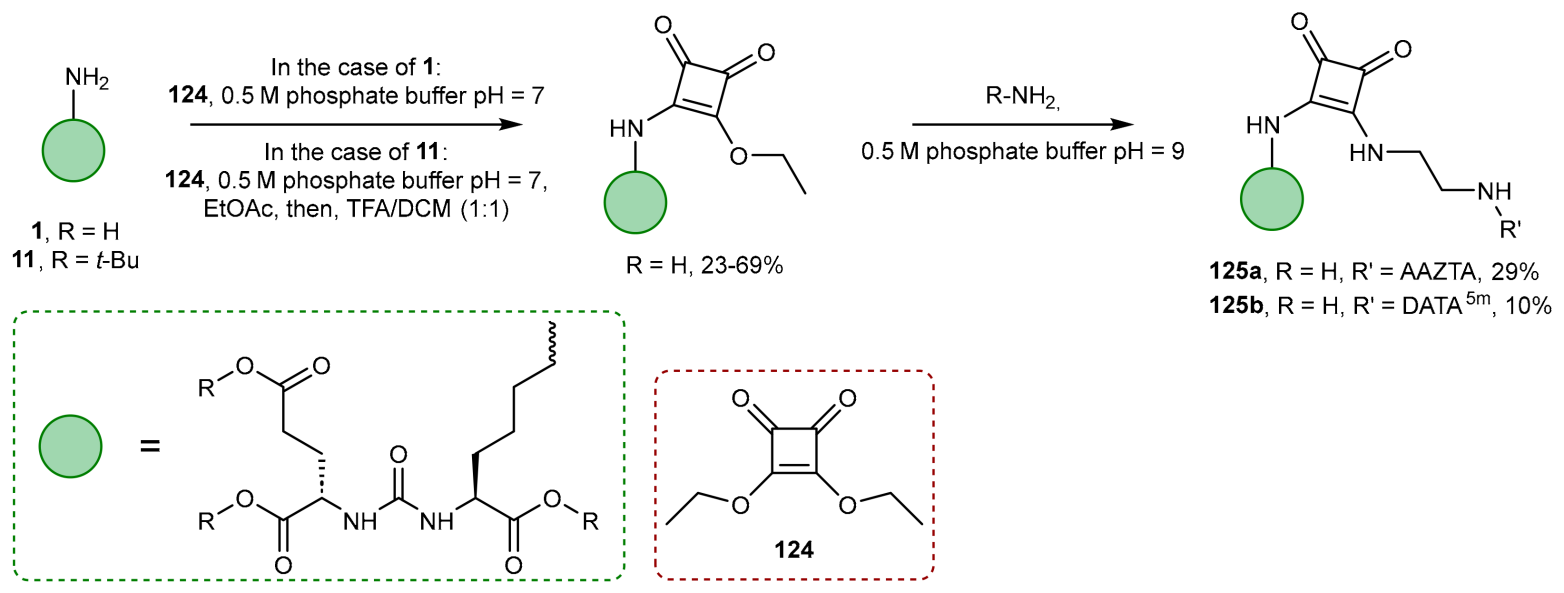
Synthesis of PSMA-targeted compounds with a squaric acid residue.

Another cyclic structural motif that has found application as a linker fragment is squaric acid. The use of its diethyl ester (compound **124**, Scheme 61) allows conjugation of two amines under preparatively simple conditions: reaction with the first amine is carried out at pH = 7, and coupling with the second amine is carried out at pH = 9 [129,130]. Due to the simplicity of achieving the selectivity of the reaction, this approach has also found application in the synthesis of PSMA-targeted compounds. For example, in [54,131], the compounds shown in Scheme 61 containing chelators AAZTA (**125a**) and DATA^5m^ (**125b**) were synthesized. It is worth mentioning that the authors have proposed various synthetic schemes for the production of target compounds. In [131], unprotected DCL urea **1** was introduced into the reaction with squaric acid diethyl ester and the yield of the target product was a moderate 23%. The subsequent reaction to obtain compound **125a** also proceeded with a relatively low yield (29%). In the synthesis of compound **125b**, the authors used an approach reacting compound **11** with **124**. The yield at this stage was 77%, after which acid-labile protective groups were removed (the yield of the protective group removal stage was 83%, and the total yield for two stages was 64%). The stage of coupling with the chelator, however, also proceeded with a low yield of the target compound (10%).

Squaric acid is also used in the linker of PSMA-targeted compounds described in [132]. Its fragment bound the urea-based ligand DCL to desferrioxamine B. The modified vector fragment was introduced by reaction in 0.1 M borate buffer (pH = 9); however, the yields were also, as in the previously discussed examples, quite modest (16% and 17% for different structural motifs).

**Scheme 62:**
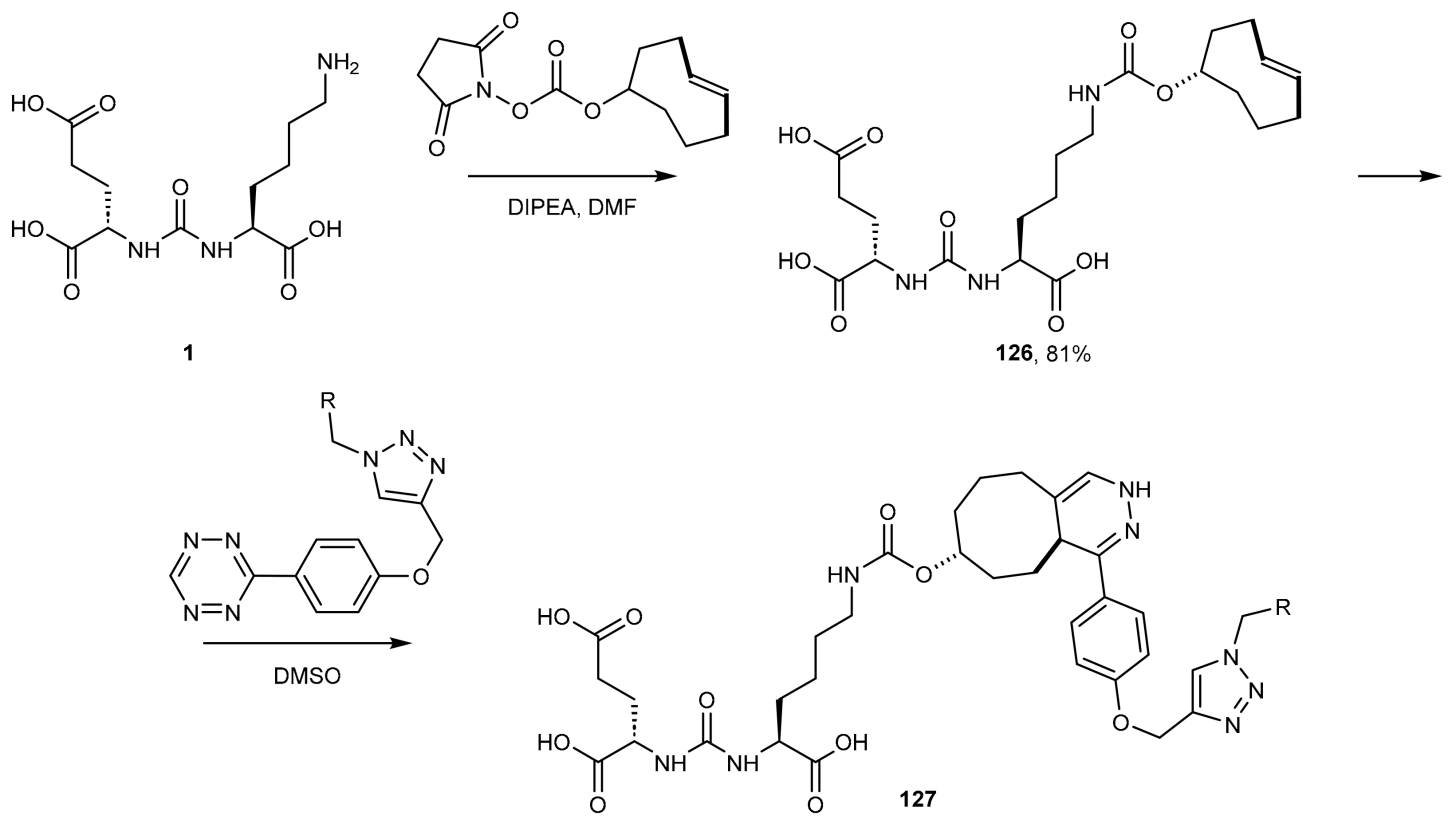
Synthesis of compound **127** by click reaction.

Linkers containing more complex cycle systems are also worth mentioning. Thus, in [133], the authors introduced urea **1** into the reaction of carbamate **126** formation (Scheme 62). The resulting compound contained a fragment of (*E*)-cyclooctene, which was subsequently reacted with a tetrazine-containing compound, the structure of which includes the chelating agent DOTA and a fluorine-containing fragment, for subsequent labeling with radionuclide ^18^F. The structure of the introduced fragment also contained other cyclic fragments—a benzene ring and a triazole cycle. The compound **127** obtained in this way already contained a radionuclide and was not characterized in detail; however, the authors showed that only one isomer of the product was formed as a result of the click reaction. It should be mentioned that the reaction itself proceeded quite quickly—5–10 min. The authors also demonstrated the possibility of carrying out this reaction in vitro, when PC cells (LNCaP) were pre-incubated with compound **126**, after which they were treated with tetrazine to obtain compound **127** linked to PSMA.

**Scheme 63:**
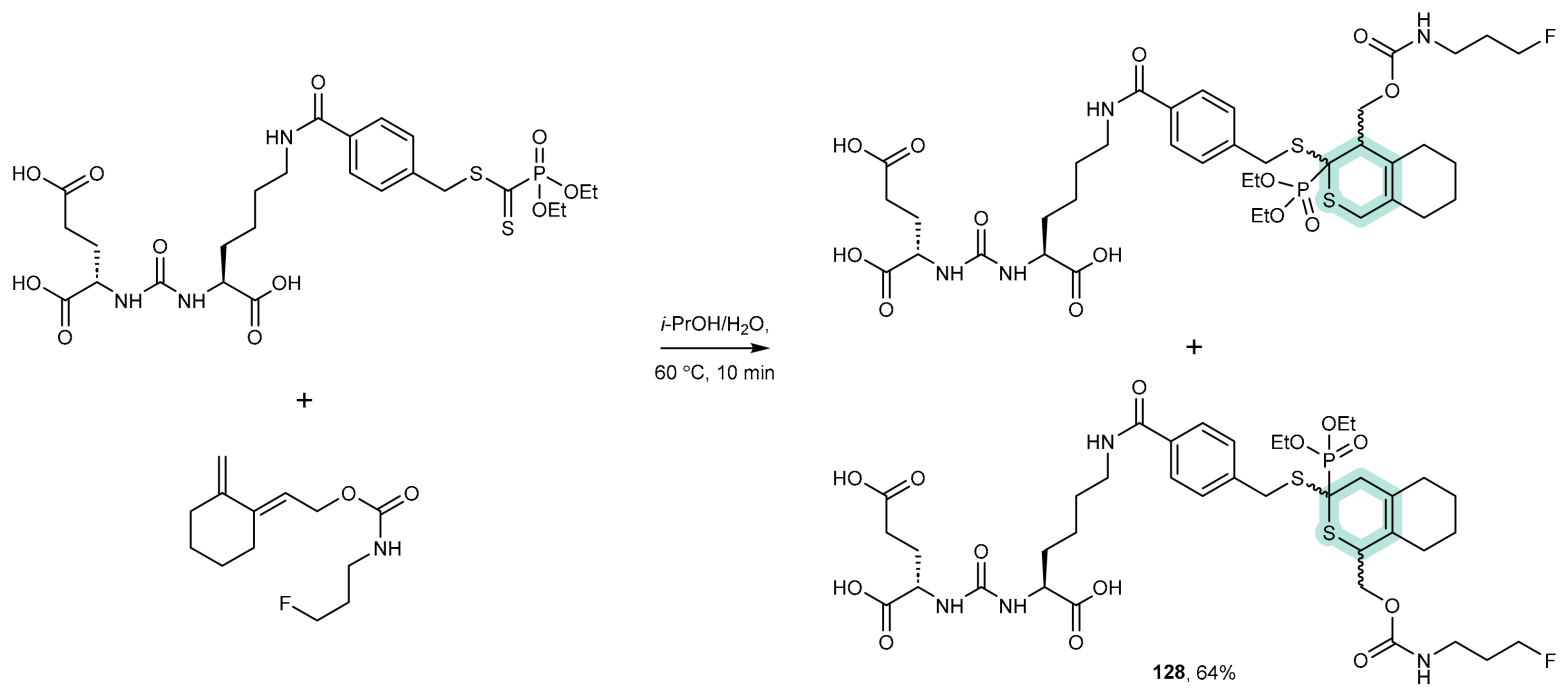
Synthesis of PSMA-targeted compound **128** using the hetero-Diels–Alder reaction.

Another example of obtaining a DCL-based compound with a condensed cyclic system in a linker was proposed in [44]. The authors carried out a hetero-Diels–Alder reaction between a phosphondithioester containing a DCL fragment and a diene containing a 3-fluoraminopropane fragment to obtain derivative **128** (Scheme 63). This compound was subsequently isotopically exchanged to form a ^18^F-labeled compound. It is worth emphasizing that as a result of the cycloaddition reaction, two regioisomers and two stereoisomers were formed, which was confirmed by the authors using ^31^P and ^19^F NMR spectroscopy. Despite the fact that the reaction proceeded with a fairly high yield (64%), it was not possible to isolate the regioisomers individually.

**Scheme 64:**
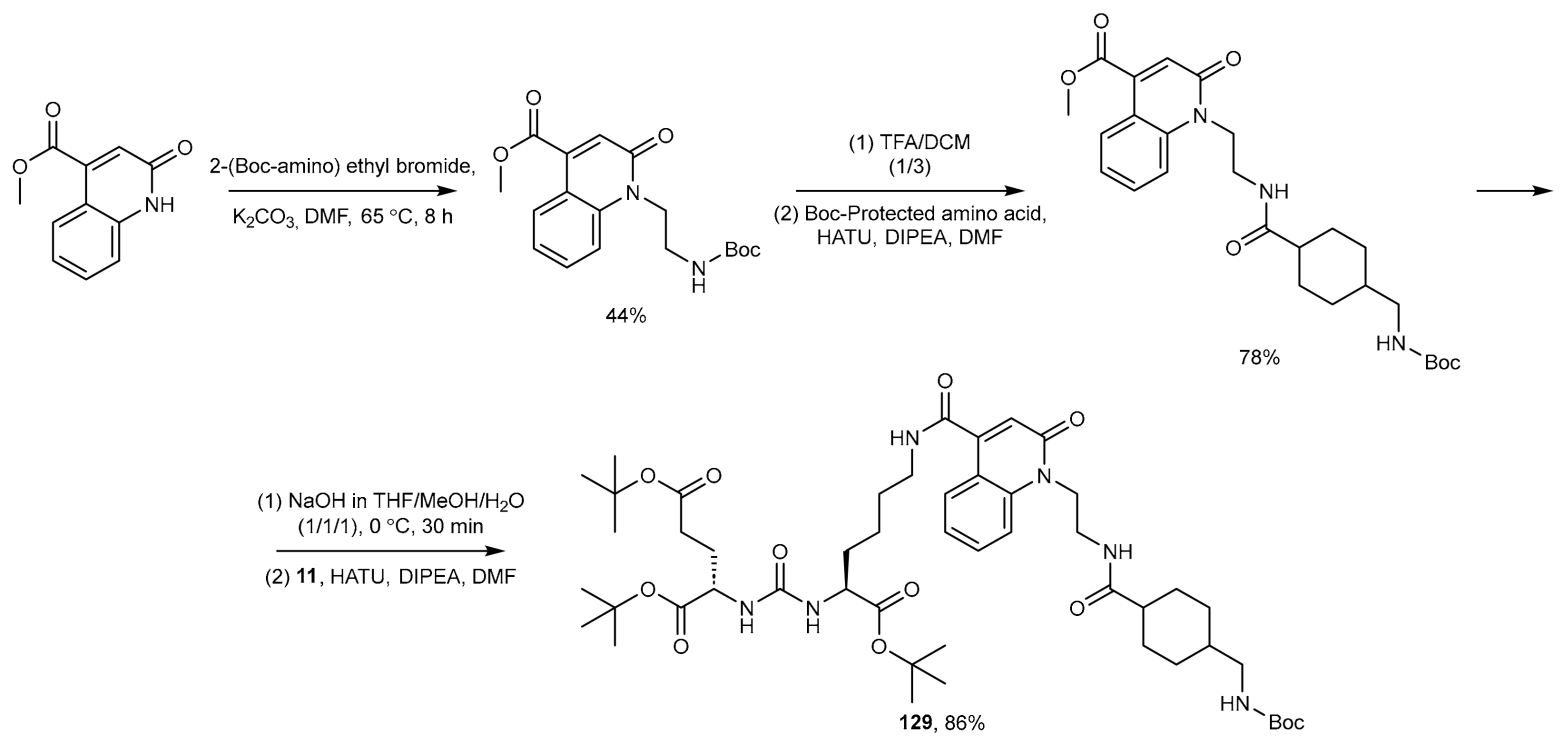
Synthesis of protected ligand **129**.

In the article [134], the authors proposed a method for the synthesis of conjugates of PSMA ligands with the chelating agent DOTA. The linker structure contained residues of tranexamic acid and 2-oxo-1,2-dihydroquinoline-4-carboxylic acid (Scheme 64). The paper describes the synthesis of three conjugates, but their synthesis is similar in many aspects, so one example will be considered. The synthesis was carried out from the methyl ester of 2-oxo-1,2-dihydroquinoline-4-carboxylic acid, which was alkylated with 2-(Boc-amino)-ethyl bromide in the presence of potassium carbonate. After that, the Boc-protection was removed and the product was introduced into an acylation reaction with Boc-protected tranexamic acid in the presence of HATU and DIPEA. Then, alkaline hydrolysis of the ester was carried out and DCL urea was acylated with the resulting carboxylic acid to obtain the protected ligand **129**. All stages except alkylation proceed with fairly high yields.

**Scheme 65:**
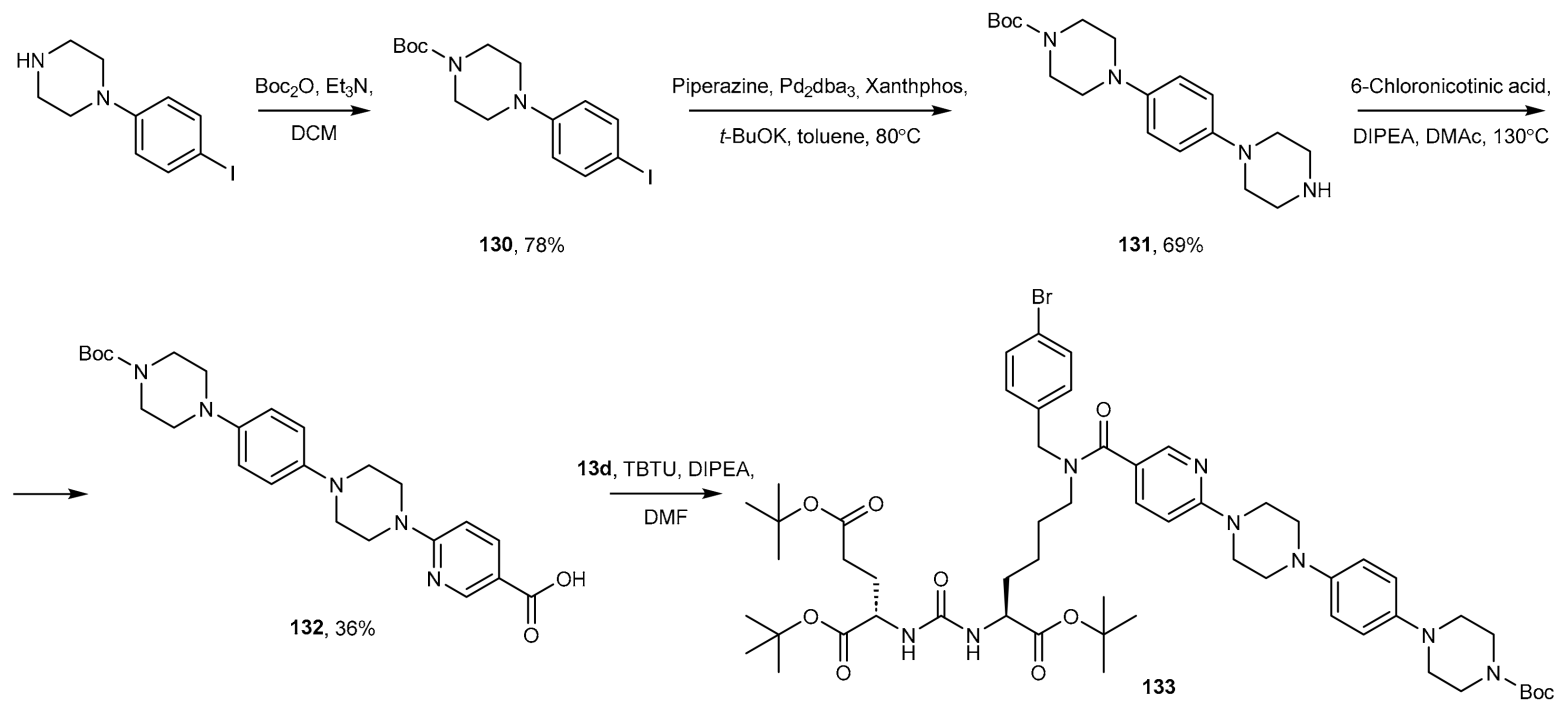
Synthesis of PSMA-targeted compound **133** containing cyclic fragments in a linker.

One more design option for PSMA ligands with several cyclic fragments in the linker is presented in [135] (Scheme 65). In the first stage, 1-(4-iodophenyl)piperazine was reacted with di-*tert*-butyl dicarbonate to give compound **130**. After that, Buchwald–Hartwig amination of compound **130** with piperazine was performed. Then, compound **131** was alkylated with 6-chloronicotinic acid. After this, the resulting linker fragment **132** was introduced into an acylation reaction with protected urea **13b** to form compound **133**.

**Scheme 66:**
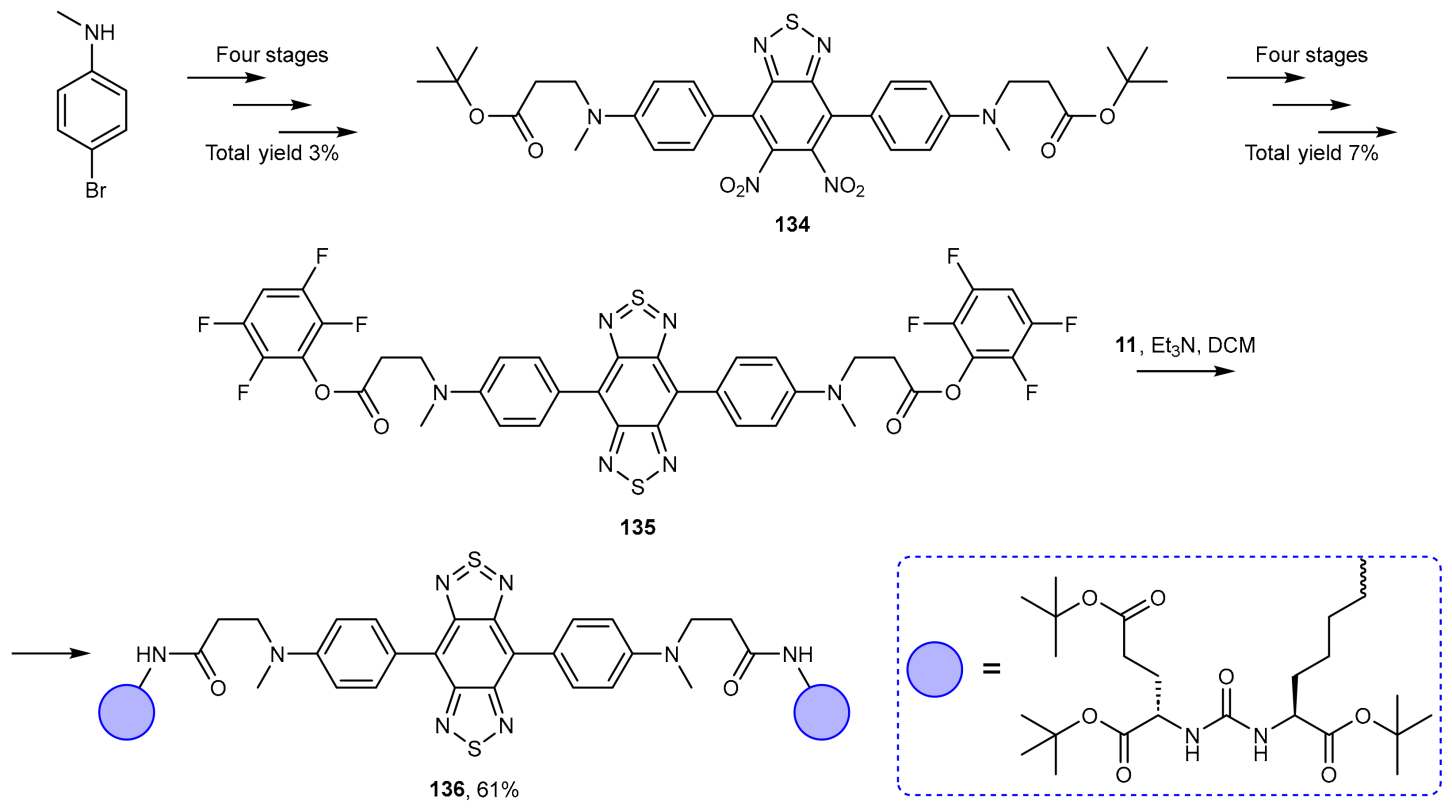
Synthesis of NIR-II conjugate **136**.

Another area where cyclic systems are used in the creation of PSMA-targeted compounds is the synthesis of ligands and conjugates containing two residues of the DCL vector molecule. For example, this design option was mentioned earlier when discussing the approaches presented in [89]. In some cases, cyclic fragments play not only the role of a linker, but also the role of a diagnostic load. Thus, in the publication [136] devoted to the synthesis of a conjugate for optical imaging in near-infrared window II (NIR-II), the authors synthesized a fluorophore that was a system of condensed cycles, which was connected to vector molecules through fragments of aniline (Scheme 66). The authors started from 4-bromo-*N*-methylaniline, which was subjected to the following transformations: alkylation with acrylic acid, introduction of *tert*-butyl protective groups, substitution of bromine with a tributylstanyl group, and Stille cross-coupling with 4,7-dibromo-5,6-dinitrobenzo[*c*][1,2,5]thiadiazole. The nitro groups of the resulting derivative **134** were reduced, after which cyclization was carried out, *tert*-butyl protective groups were removed and TFP-ester **135** was obtained. After that, it was reacted with compound **11** to obtain a protected form of conjugate **136**. It is worth noting that the synthetic scheme proposed by the authors can be optimized, since some stages proceeded with fairly moderate yields (8–36%). The total yield of the described scheme was only 0.13%.

**Scheme 67:**
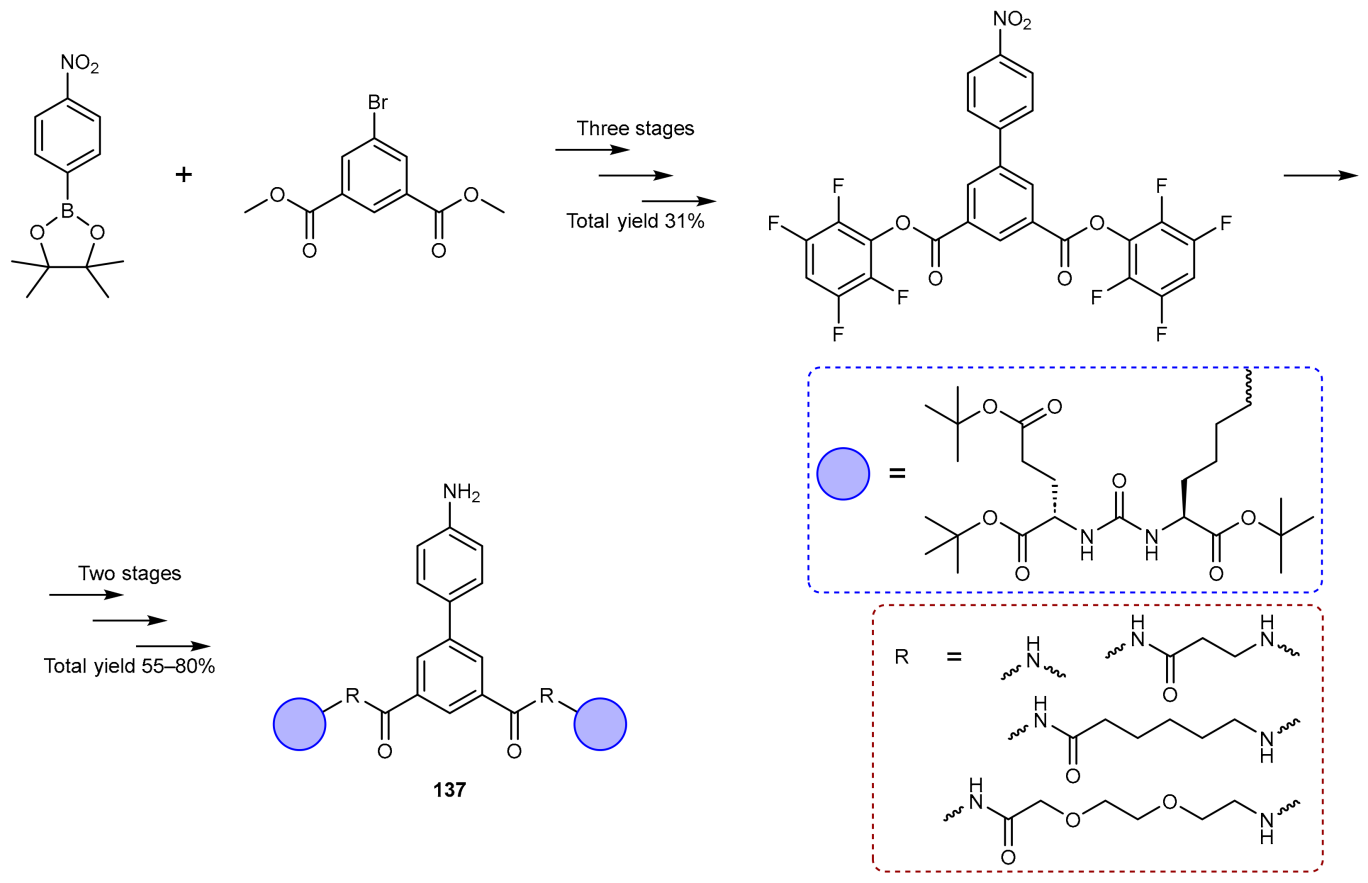
Synthesis of the protected PSMA ligand **137**.

An interesting example of the use of cyclic fragments in a linker for joining two DCL residues can be found in [137]. The authors obtained protected ligands with aniline fragment **137** with linkers of a different length between aromatic and vector fragments (Scheme 67). The authors proceeded from dimethyl ester of 5-bromoisophthalic acid and nitro-substituted aryl pinacol-boric ether. In the first stage, the Suzuki cross-coupling reaction followed by alkaline hydrolysis of the ester was carried out and the resulting diacid was converted to TFP-ester. The acylation reaction of compound **11** or its acylated derivatives was then carried out, after which the nitro group was reduced to produce the protected ligand **137**. The total yield of this synthetic scheme ranges from 17% to 25%.

Acylation of the ε-amino group of DCL lysine remains the primary method for introducing cyclic linkers. However, linker synthesis requires multistep transformations including click reactions (CuAAC), cross-couplings, condensation, and heterocycle formation (such as hetero-Diels–Alder reaction). Route selection depends on both target structure and starting material availability.

**Scheme 68:**
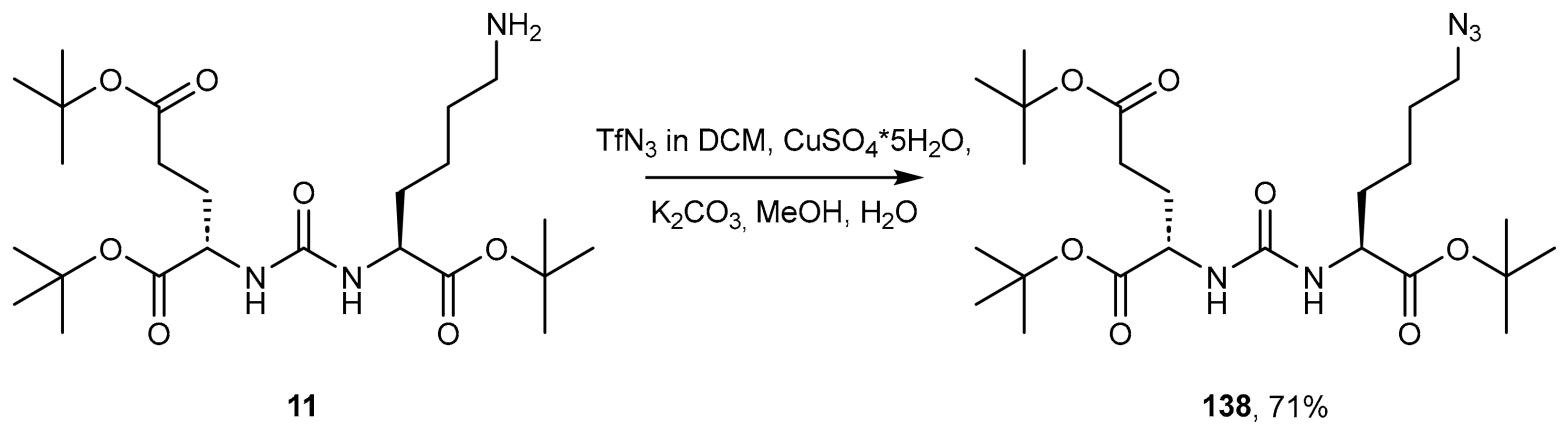
Synthesis of the azide-containing analog of DCL urea **138**.

The most common strategies for obtaining DCL-based ligands have been discussed above, but some studies have proposed methods that go beyond the above systematization. In this part of the paper, we will look at some detailed examples. The transformation of the ε-amino group of lysine into an azide group can be attributed to such variations in the modification of DCL urea. In [32], the authors reacted compound **11** with TfN_3_ to produce compound **138** (Scheme 68). The reaction was carried out in a mixture of water and alcohol in the presence of CuSO_4_*5H_2_O and potassium carbonate. TfN_3_ was added into the reaction mixture in the form of a solution in dichloromethane. The product yield was 71%. Subsequently, this compound was introduced into an azide-alkyne cycloaddition reaction with various substrates.

## 4. Solid-Phase Peptide Synthesis as a Method for Obtaining PSMA Ligands

In this section, we consider techniques for obtaining PSMA-targeted compounds using synthesis on a solid-phase carrier. This group of methods involves the obtainment of a resin-immobilized urea fragment and sequential synthesis of the final compound using solid-phase peptide synthesis. And it is most often used for the synthesis of conjugates of PSMA-ligands with various chelating agents. In this case, the protected form of the conjugate is synthesized on a solid-phase carrier, after which the substance is removed from the carrier and the protective groups are subsequently removed.

**Scheme 69:**
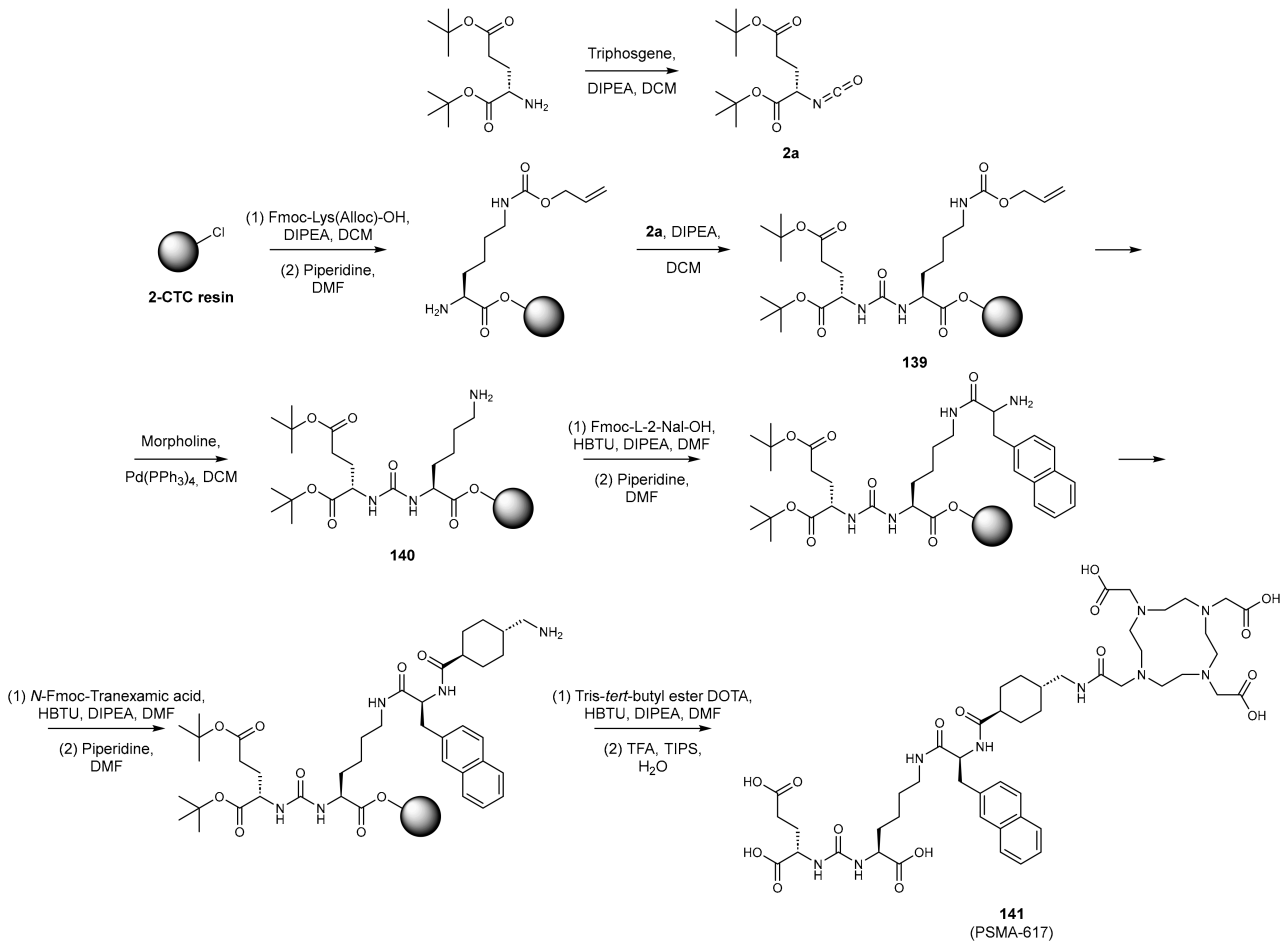
Solid-phase synthesis of the conjugate PSMA-617.

An example of using a solid-phase approach is the synthesis of the conjugate PSMA-617 (Scheme 69).

The first stage of this scheme involved the production of glutamic acid isocyanate **2a**. After that, the lysine residue protected by the allyloxycarbonyl (Alloc) group at the ε-position and the Fmoc group at the α-position were immobilized on the 2-CTC resin. Then the Fmoc group was removed and a urea fragment was created by reaction with isocyanate **2a** to form the immobilized protected form of urea DCL **139**. The next step was the selective removal of the Alloc group under the action of morpholine in the presence of tetrakis (triphenylphosphine)palladium to obtain the derivative **140** suitable for further modification by the amino group. In the case of PSMA-617, subsequent modification included the introduction of 2-naphthylalanine and tranexamic acid residues using the classical Fmoc strategy, acylation of the amino group of tranexamic acid with tris-*tert*-butyl ester of the DOTA chelator and removal of the resulting product from the resin, while simultaneously removing acid-labile protective groups by the action of a mixture of trifluoroacetic acid, tri-*iso*-propylsilane (TIPS) and water. This made it possible to obtain the target conjugate **141**. Despite the fact that alternative approaches to obtaining the PSMA-617 conjugate have been proposed in the literature [99], it is usually the solid-phase strategy that is used. Similar synthesis schemes have been implemented in a number of publications [138,139,140,141].

**Scheme 70:**
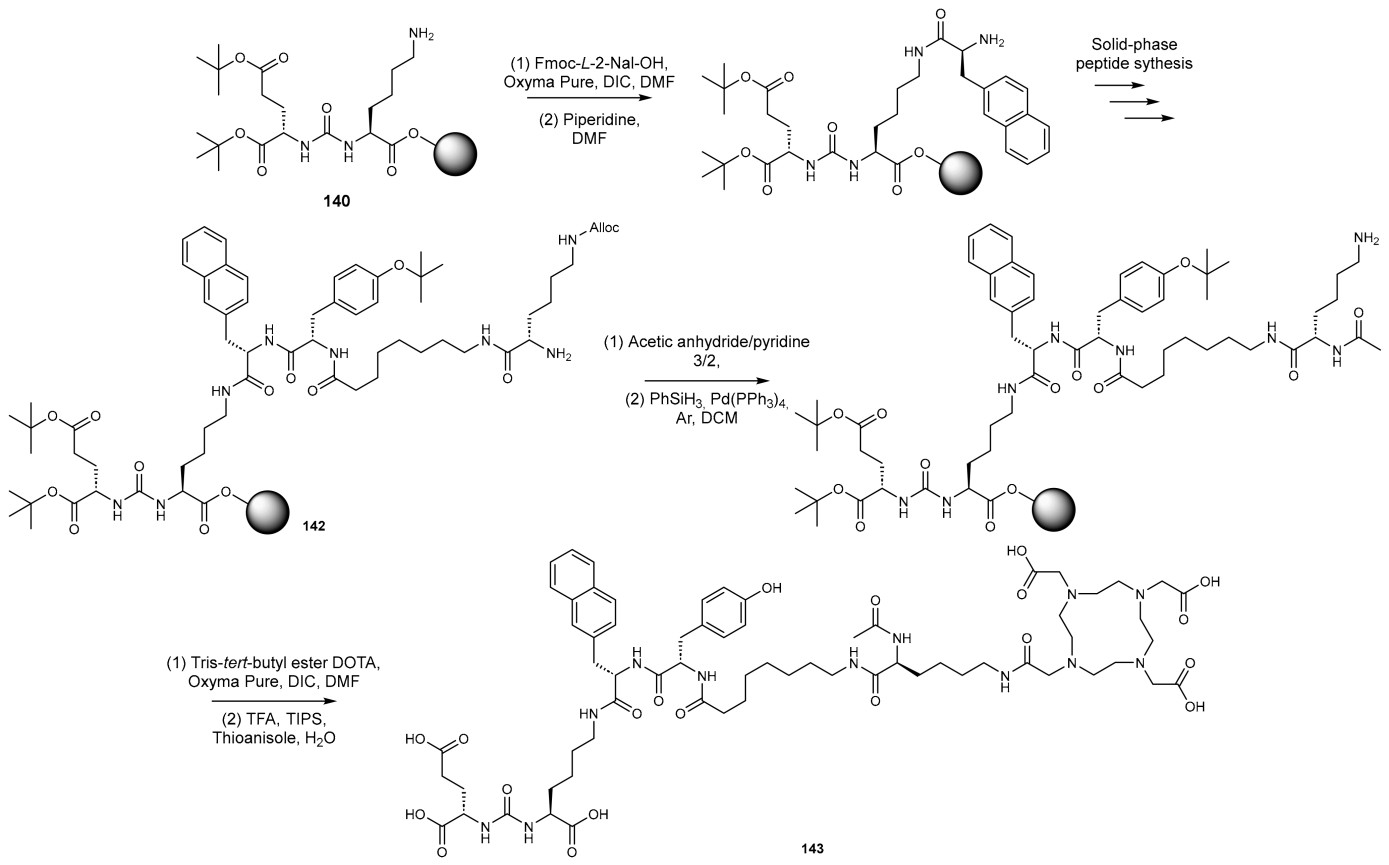
Solid-phase synthesis of conjugate BQ7876.

The approach described above has found extremely extensive application in the synthesis of various conjugates of PSMA ligands with chelating agents. One example of using such a strategy is the synthesis of a conjugate with chelator DOTA BQ7876 and its analogs, presented in [141,142]. Also, as in the above procedure, the authors carried out synthesis on a solid-phase carrier of compound **140**. After that, a linker fragment representing the following amino acid sequence was assembled: *L*-naphthylalanine, *L*-tyrosine, 8-aminooctanoic acid and α-Fmoc-ε-Alloc-*L*-lysine (Scheme 70). It is worth mentioning that, unlike the previously discussed example with PSMA-617, HBTU was not used for acylation, but a combination of DIC and Oxyma Pure (ethyl cyano(hydroxyimino)acetate). The use of such a combination was aimed at minimizing racemization and increasing the yield of the target product [143]. Thus, the immobilized derivative **142** was obtained, which was introduced into the acylation reaction with acetic anhydride, subjected to the removal of the protective Alloc group, acylated with a protected DOTA chelator, and removed from the solid-phase carrier simultaneously with the removal of the protective groups. Thus, the target conjugate BQ7876 (compound **143**) was obtained.

**Scheme 71:**
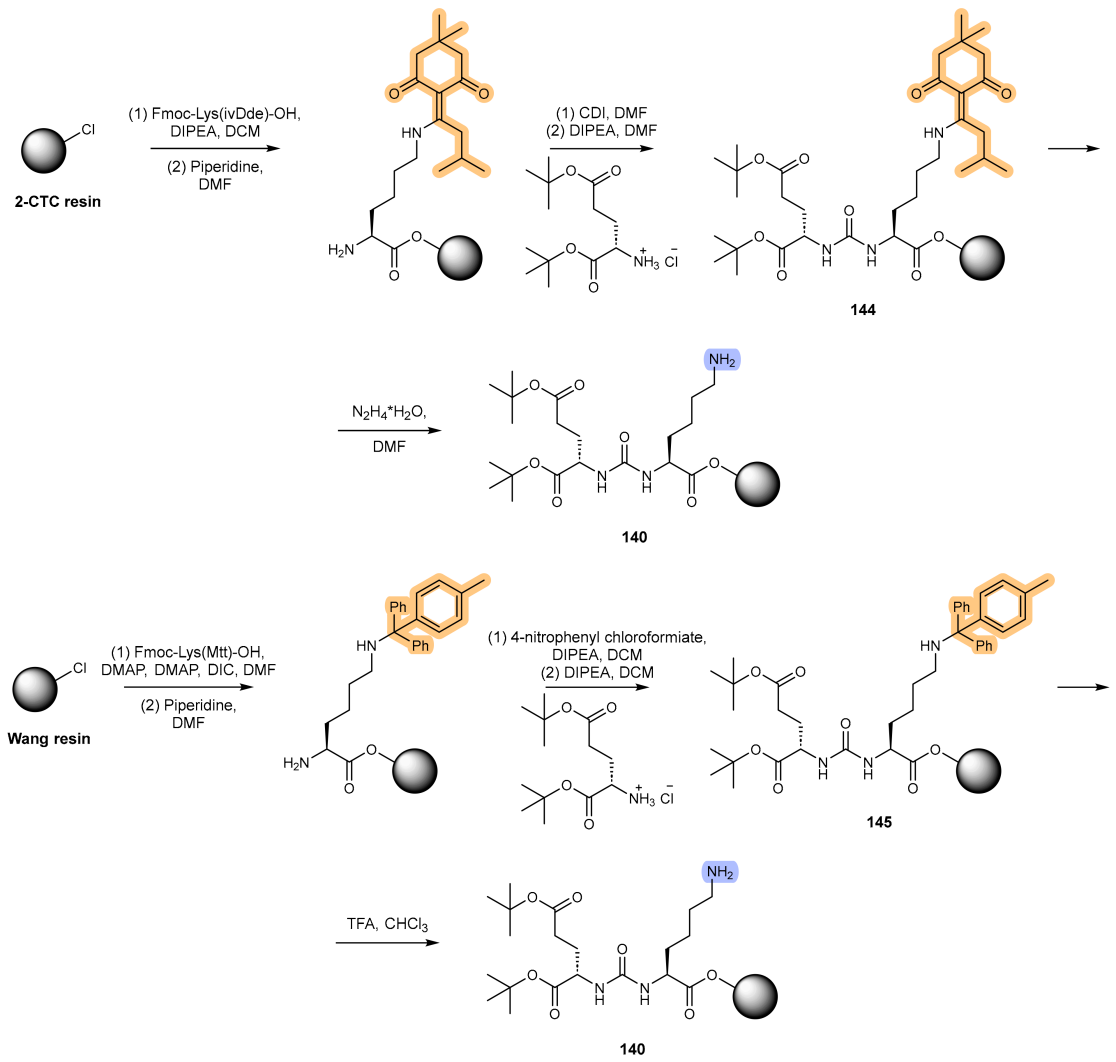
Alternative approaches to creating a protected DCL fragment on resin. The protective groups are highlighted in orange, and the lysine amino group is highlighted in blue.

The strategy of obtaining immobilized DCL urea using an Alloc protective group is not the only option; several alternative options for solid-phase synthesis of this fragment on resin are presented in the literature (Scheme 71). Thus, there is an example of the use of a 1-(4,4-dimethyl-2,6-dioxohex-1-ylidene)-3-methylbutyl (ivDde) protective group of the ε-amino group of lysine [144]. The authors of this publication obtained the urea fragment by a reaction involving CDI, which is also a less common approach in solid-phase synthesis. The resulting derivative **144** was subsequently reacted with a hydrazine hydrate to remove the protective group to produce the immobilized fragment **140**.

In another alternative, a 4-methyltrityl (Mtt) protective group was also used to produce the urea fragment on the resin (Scheme 71) [145]. The choice of an alternative protective group in this example was justified by the solid-phase carrier used. Unlike the previously considered cases, the authors used Wang resin. In the first stage, the protected lysine was immobilized on the resin and the Fmoc protective group was removed, after which urea was obtained using 4-nitrophenyl chloroformate and di-*tert*-butyl ester hydrochloride of glutamic acid. The derivative **145** obtained in this way was introduced into the reaction of removing the Mtt protective group under the action of a 1.8% solution of trifluoroacetic acid in chloroform. Under such conditions, removal takes place selectively, without affecting the *tert*-butyl groups of the glutamic acid residue, and the immobilized fragment was not removed from the carrier.

**Scheme 72:**
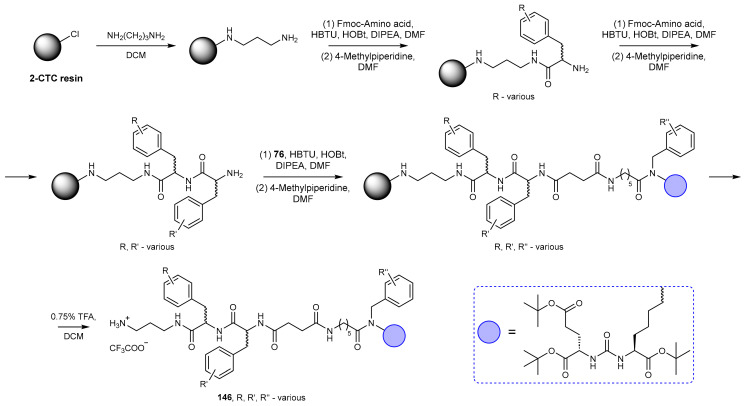
Synthesis of protected ligands based on a fragment of 1,3-diaminopropane immobilized on 2-CTC resin.

The approaches to the solid-phase synthesis of protected forms of ligands presented in the works [146,147] are also worthy of mention. Unlike the examples discussed above (Scheme 69, Scheme 70 and Scheme 71), this method involves immobilization on a 2-CTC resin, not an amino acid residue but a 1,3-diaminopropane residue (Scheme 72). The methodology described in the paper [148] was used as the basis for this stage. After this, the amino group of diaminopropane was subjected to modification using a standard Fmoc strategy. Thus, it was possible to synthesize a series of ligands, **146**. At the same time, in the work [146] there are certain limitations of this method due to the fact that removal from the resin proceeds more slowly than with the “classical” immobilization of the molecule on the resin with a carboxyl group. This led to the formation of a mixture of the target compound and a derivative that did not contain a *tert*-butyl protective group at the oxygen atom of the tyrosine residue in the linker fragment.

## 5. Removal of Protective Groups

Usually, the last step in the synthesis of PSMA ligands or some conjugates based on them is the removal of protective groups. Regarding the synthesis of PSMA ligands, acid-labile protective groups are used, and the conditions for removing protective groups by trifluoroacetic acid are the most widespread.

**Scheme 73:**
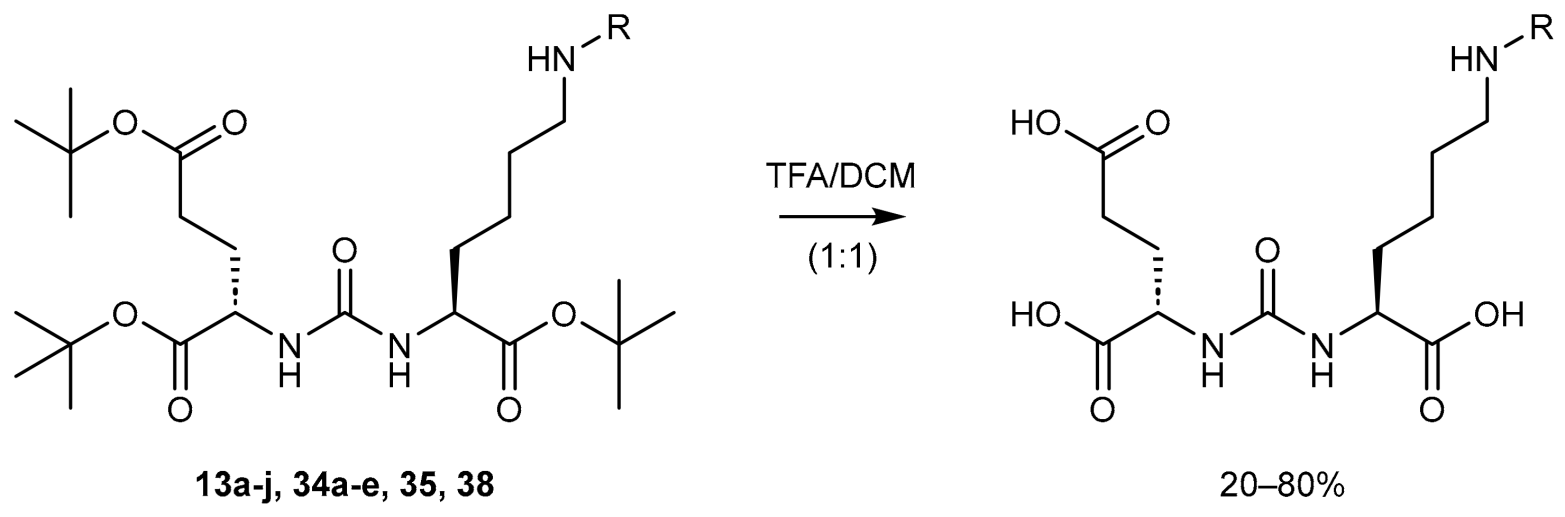
A general scheme for the removal of protective groups, presented in [46].

Thus, when preparing PSMA ligands with various aromatic fragments at the ε-amino group of lysine, based on compounds **13a**–**j**, **34a**–**e**, **35** and **38**, a mixture of TFA and DCM in a 1:1 ratio was used for deprotection (Scheme 73) [46]. In this manner, it was possible to obtain target ligands with yields of 20–90%.

**Scheme 74:**
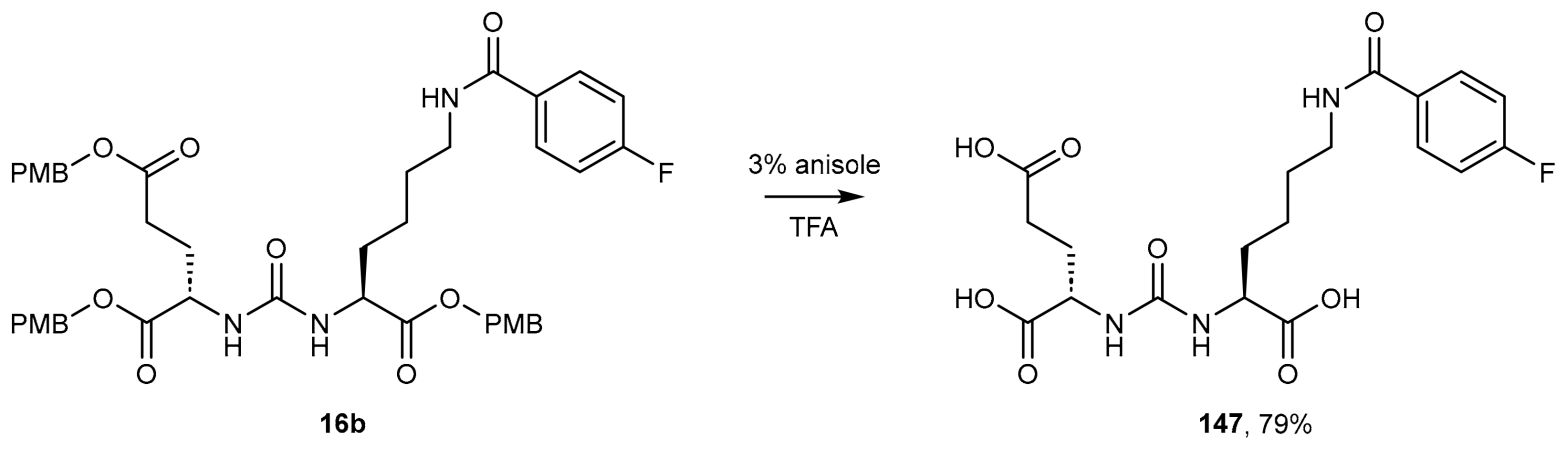
Removal of PMB protective groups using a mixture of anisole and TFA.

In general, the approach using TFA solutions in methylene chloride of various concentrations is used in the largest set of studies. It can also be used to remove PMB protective groups, which was implemented using some examples in [71,72,108]. However, as an alternative, the authors in [71,72] used a mixture of anisole and TFA (Scheme 74). For example, such conditions were used in the preparation of ligand **147**. The anisole solution was added at 0 °C and the resulting mixture was stirred for 20 min at room temperature. The product was obtained with a 79% yield.

A side process that strongly affects the simplicity of PSMA ligand isolation and the yield of target compounds is the alkylation of nucleophilic atoms by carbocations formed during acidolysis. Several modified techniques are used to prevent these processes from occurring. Thus, various trialkylsilanes are often added to the mixture to remove protective groups as scavengers [149]. For example, in studies devoted to the synthesis of PSMA-I&T and its analogs, a mixture of TFA and DCM with the addition of TIBS was used to obtain modified peptides, **71** (Scheme 38). A mixture with the addition of TIPS was used in [116]. Also, in the above example of the synthesis of conjugate BQ7876 and its analogs (Scheme 70), the authors used a mixture of TFA, water, thioanisole and TIPS. There, tri-*iso*-propylsilane and thioanisole also acted as a scavenger.

## 6. Examples of the Various Synthetic Approaches Use to Obtain PSMA-Targeted Diagnostic and Therapeutic Conjugates

**Scheme 75:**
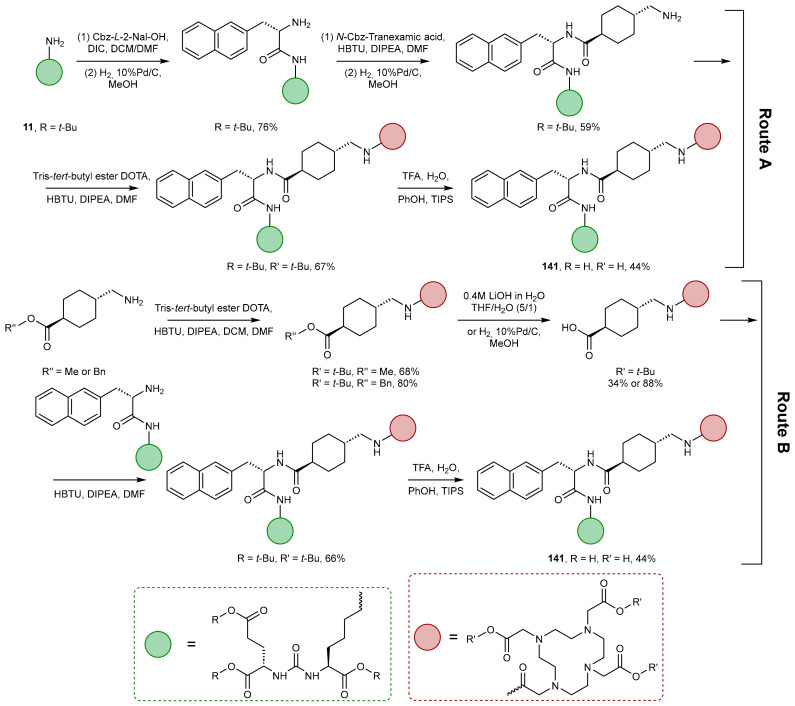
Approaches to the synthesis of the PSMA-617 conjugate without using solid-phase techniques.

The synthetic approaches described in the previous sections to obtain modified forms of DCL urea can be used to produce some diagnostic and therapeutic compounds aimed at treating prostate cancer. In Section 4, a synthetic scheme for obtaining the conjugate PSMA-617 (Scheme 69) was considered in detail. The ^177^Lu-labeled derivative of this compound is approved as a radiotherapy drug by the FDA [18]. However, the approaches discussed in Section 2 and Section 3 allows synthesis of the target compound **141** without using solid-phase methods, which was shown in [99]. The authors proposed two synthetic strategies for obtaining PSMA-617. The first one involved the preparation of compound **11** using DSC, after which the introduction of a peptide linker was carried out using methods of peptide synthesis using a Cbz protecting group (Scheme 75, route A). The total yield of compound **141** using this scheme was 13%. The second approach (Scheme 75, route B) involved modification of compound **11** with Cbz-protected *L*-naphthylalanine. After that, the protective group of the α-amino group of naphthylalanine was removed. At the same time, the tris-*tert*-butyl ester of DOTA was introduced into an acylation reaction with benzyl or methyl ester of tranexamic acid, followed by removal of the protective group at the carboxyl group. The compounds obtained in this way were introduced into an acylation reaction followed by the removal of protective groups. In this manner, compound **141** was obtained, and the total yield of the synthetic scheme was 22% based on compound **11**.

**Scheme 76:**
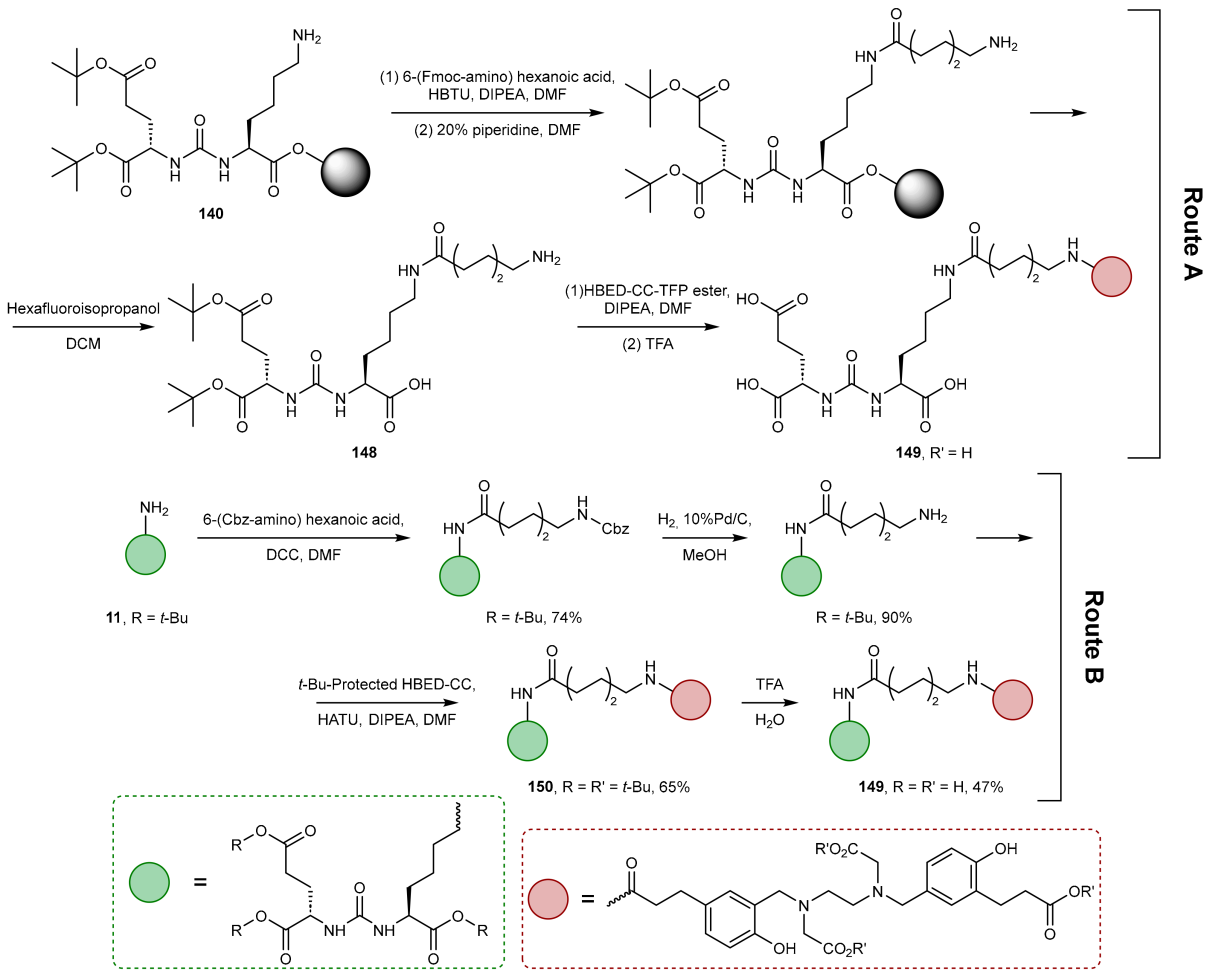
Approaches to the synthesis of the conjugate PSMA-11.

In the case of diagnostic conjugates, synthetic schemes to obtain drugs for PET/CT imaging are of particular interest. Earlier in this review, a method for the synthesis of a protected precursor compound of the drug Pylarify™ (compound **18a**, Scheme 13) has already been described. Another FDA-approved drug for the diagnosis of prostate cancer is the radiolabeled conjugate ^68^Ga-PSMA-11 [17]. As well as in the synthesis of PSMA-617, the solid-phase synthesis method of this compound has become the most popular (Scheme 76, route A) [138]. The following synthetic scheme was used: compound **140** was acylated with 6-(Fmoc-amino)hexanoic acid in the presence of HBTU, the protective group was removed under the action of piperidine, after which compound **148** was removed from the solid-phase carrier under the action of hexafluoroisopropanol. Compound **148** was acylated with the TFP-ester of the HBED-CC chelator, then the acid-labile protective groups were removed by TFA to obtain the target compound **149**. However, an alternative aimed at obtaining this compound without using solid-phase synthesis is also presented in the literature (Scheme 76, route B) [150]. This approach involved acylation of compound **11** with 6-(Cbz-amino)hexanoic acid in the presence of DCC during the first stage, subsequent removal of the protective group by hydrogenolysis and the introduction of the resulting compound into an acylation reaction with the *tert*-butyl protected chelator HBED-CC in the presence of the coupling agent HATU. The protected precursor compound **150** obtained in this way was treated with a mixture of TFA and water to obtain the target product **149**. The total yield of PSMA-11 during synthesis, according to this scheme, was 20%.

It is worth noting that in the case of both PSMA-617 and PSMA-11, some different synthetic approaches to obtain modified DCL urea, which were previously presented in this review (Section 3.1 and Section 3.2), can be applied.

## 7. Synthetic Strategy Selection Based on Target Structure

**Figure 6:**
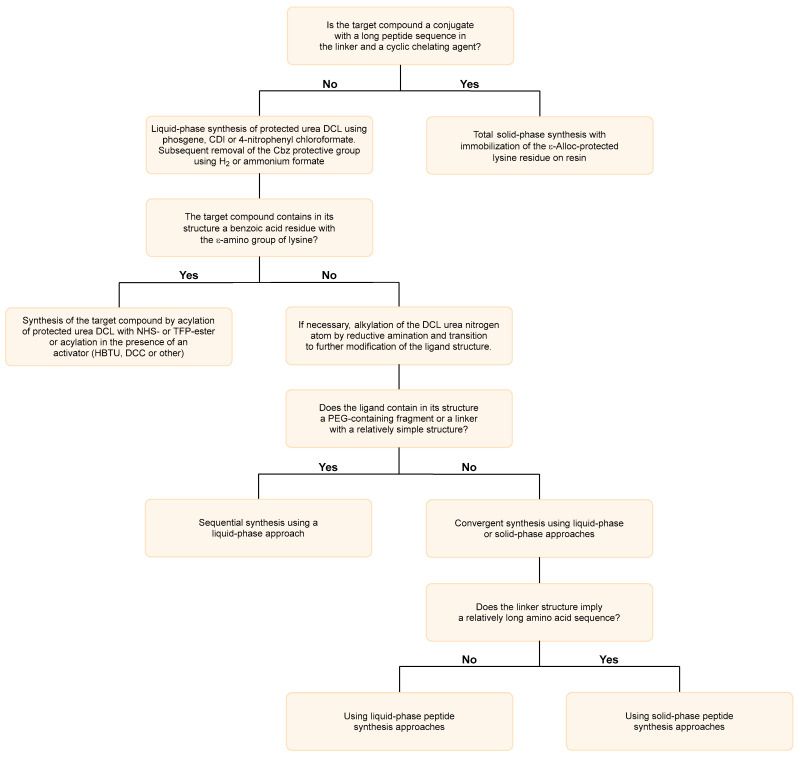
Decision tree for PSMA ligand synthesis strategy selection.

This section presents a decision tree for selecting PSMA ligand synthesis strategies, as shown in Figure 6.

In the first stage, the method for assembling the urea fragment should be determined. In the synthesis of conjugates incorporating complex peptide-based linkers and cyclic chelators such as DOTA, DOTA-GA, NOTA, and related compounds, fully solid-phase synthesis is generally the preferred approach. This strategy has been successfully applied in the synthesis of compounds such as PSMA-617 and BQ7876 (Section 4), although it is not without limitations. Solid-phase peptide synthesis is more difficult to scale up, and monitoring the progression of intermediate steps presents certain preparative challenges. Moreover, structural confirmation of intermediate compounds by spectroscopic methods requires test cleavage of the immobilized fragment from the resin. Another major limitation of SPPS-based approaches is that fragment coupling can be achieved only through an acylation step. Nevertheless, advantages such as ease of automation, rapid reaction rates, and straightforward purification between steps make SPPS a highly convenient tool for the preparation of PSMA-targeting compounds. The most widely used approach involves phosgene for isocyanate generation and ε-Alloc-protected lysine as the first immobilized amino acid (Scheme 69). This route is relatively simple from a preparative standpoint and has been described in detail in the literature, which makes it the principal option for such synthetic schemes. Approaches using CDI or 4-nitrophenyl chloroformate (Scheme 71) are also suitable for this stage, although they may require additional optimization when the Alloc protecting group is used instead of Dde or Mtt.

For the synthesis of ligands containing fragments that are not simple peptide sequences, as well as ligands with a relatively simple structure similar to that of Pylarify™, a solution-phase approach is more convenient. When choosing between the PMB-protected and *t*-Bu-protected DCL derivatives, compound **3** is the more attractive option. The orthogonality of the protecting groups makes it possible to obtain derivative **11** in a higher overall two-step yield than compound **12**. When selecting a strategy for constructing the urea bridge, three options are of particular interest: the triphosgene-based approach under strong cooling, the CDI-based method, and the 4-nitrophenyl chloroformate route (Section 2.1). The first two options are described in the literature in greater detail. The triphosgene-based variant is more laborious, but it affords a somewhat higher yield. The CDI-based approach is simpler from a preparative standpoint and avoids the use of toxic triphosgene. The 4-nitrophenyl chloroformate route provides the highest yields; however, it is less thoroughly described in the literature, so its scalability remains somewhat unclear.

When selecting a method for removing the Cbz protecting group, two main options are available. The first is a method with hydrogen, which is somewhat more laborious because it involves working with an explosive gas. At the same time, it is the most thoroughly described and optimized approach. A good alternative is the use of ammonium formate, which is somewhat simpler from a preparative standpoint. Both methods afford the target product in high yields, and it is not possible to identify a clearly preferable option.

The next step in selecting a synthetic route is modification of the ε-amino group of lysine. In the first stage, it is possible to introduce either an aromatic fragment or a linker. When an aromatic fragment is introduced, the two most common options are acylation with a substituted benzoic acid or alkylation via reductive amination (Section 3.1). Acylation appears to be the more favorable option when the synthesis is aimed at obtaining derivatives containing an aromatic moiety with a group that will later undergo substitution with a radioactive isotope, as in compounds structurally related to Pylarify™. In this case, strategies based on TFP- and NHS-esters provide somewhat higher yields than those using uronium- or phosphonium-based activators. Although the need to prepare these reagents separately may be a drawback, the use of NHS- and TFP-esters still appears preferable. If the fragment is introduced solely to increase affinity for PSMA, with further structural elaboration of the ligand planned thereafter, reductive amination should be preferred. In this case, methods employing NaBH_4_, STAB, and NaBH_3_CN are available. In many publications, the yields at this stage are rather moderate, around 50%, indicating that reaction conditions require optimization. However, the use of sodium borohydride is associated with side reactions involving reduction of the carbonyl group to the corresponding alcohol, which complicates chromatographic purification of the target product and lowers the yield. For this reason, sodium cyanoborohydride or STAB appears to be preferable.

Further introduction of the linker can be accomplished by two approaches. In the case of relatively simple linkers, the DCL urea moiety can be sequentially modified starting from the ε-amino group of lysine. Similar strategies are also commonly used for the introduction of PEG-containing linkers. The most common method for linker installation is acylation of the unprotected amino group of the DCL urea. Depending on the fragment being introduced, either activated esters (NHS or TFP) or various coupling reagents (HBTU, HATU, DCC, etc.) should be used. Both approaches are applicable, and it is not possible to identify a clearly superior one.

For more complex linkers, both linear ones and those containing cyclic fragments, a convergent synthesis is the preferable option. In this case, a linker of peptide nature can be synthesized separately using either solution-phase approaches or SPPS. When selecting the optimal strategy, the disadvantages and advantages of each approach should be taken into account—some of which have already been mentioned above and are summarized in Table 2.

Based on the data summarized in Table 2, a peptide-based linker consisting of a small number of amino acid residues (2–3) can be conveniently synthesized using a solution-phase approach. This is particularly advantageous when target quantities above ten grams are required. At the same time, for the preparation of larger series of compounds and for the incorporation of linkers with a greater number of amino acid residues, an SPPS-based strategy is preferable.

The decision-making algorithm for selecting a synthetic route outlined above provides a general framework for identifying an optimal strategy for the preparation of PSMA ligands. However, this decision tree is not exhaustive. For example, linkers containing several cyclic fragments are difficult to fit into this scheme, as their synthesis typically involves a much broader range of chemical transformations.

## 8. Future Perspectives and Conclusions

The literature analysis shows that the synthesis of protected DCL urea derivatives and the removal of protecting groups have been thoroughly studied and, in many cases, well optimized. At the same time, methods for the subsequent modification of DCL continue to evolve actively. Solution-phase synthesis, solid-phase synthesis, and their combination make it possible to access a broad range of PSMA-targeted ligands with complex structures, as well as conjugates based on them. In this context, the functional fragment intended for subsequent conjugation plays a decisive role, and groups suitable for click reactions, such as CuAAC, are attracting increasing attention and strongly influence the choice of synthetic strategy.

The prospects for further development of synthetic approaches to PSMA ligands should also be highlighted. Although many steps are already well optimized (for example, the preparation of protected urea derivatives or peptide synthesis steps), some relatively common transformations still require additional refinement. One such process is the reductive amination used to introduce a benzyl fragment at the ε-amino group of lysine. In the publications considered, the yield of monoalkylation varies substantially, from approximately 30% to about 90%. Another example is provided by methods for introducing linker fragments bearing a thiol group. This functional group is widely used for conjugating PSMA ligands to therapeutic agents [67,151]. In such conjugates, the release of the therapeutic payload is triggered by intracellular glutathione [152,153]. However, the methods for introducing such functional fragments described in this work (compounds 48 and 49) afford the corresponding products in rather modest yields. Further optimization of these synthetic protocols would enable more efficient access to PSMA ligands of this type. In this particular case, special attention should be paid to the use of protecting groups on the sulfur atom of the thiol fragment, which would prevent disulfide formation.

Another important aspect of the development of synthetic methods for PSMA ligands is the use of green chemistry approaches. To date, no studies specifically devoted to this topic have been reported. In the synthesis of PSMA ligands, solvents such as DCM, DMF, CHCl_3_, and others classified as problematic or hazardous are widely used [154]. In addition, peptide synthesis approaches are extensively employed, and these methods often involve hazardous reagents such as triethylamine. Protecting groups are also widely used, which runs counter to the principle of atom economy. At the same time, extensive literature data are available on the application of green chemistry approaches in peptide synthesis [155,156,157]. Therefore, the application of these approaches to the synthesis of PSMA ligands is a logical direction for the further development of synthetic strategies.

In this review, the main approaches to the synthesis of DCL urea-based PSMA ligands were summarized. The preparation of protected DCL urea derivatives from the corresponding amino acids was described, and the scalability of this stage, together with the differences between the reported methods of urea fragment formation, was analyzed. The principal strategies for the subsequent modification of DCL through the introduction of aromatic moieties and linker fragments were systematized. Methods for the incorporation of PEG-containing linkers and linkers incorporating various cyclic systems were also discussed. In addition, the main approaches to the introduction of more complex linkers into PSMA ligands were outlined. Fully solid-phase synthesis of PSMA inhibitors and related conjugates, as well as alternative synthetic routes for obtaining therapeutic and diagnostic PSMA-targeted conjugates, was considered. On this basis, an algorithm for selecting the optimal synthetic strategy for PSMA ligands according to the structure of the target compounds was proposed.

## Figures and Tables

**Table 1:** Methods for the synthesis of protected DCL ureas (**3**, **10**).

Target Compound	Reagent for Urea Fragment Formation	Temperature, °C	Purification Method	Yield, %	Scalability
**3**	Triphosgene	0	Chromatography (except a few examples)	28–82	From hundreds of mg to tens of g
		−90–−78		56–92	From hundreds of mg to tens of g
	CDI	0 then 40		69–95	From hundreds of mg to tens of g
	DSC	rt		53–76	From hundreds of mg to g
	*para*-Nitrophenyl chloroformate	0 then rt		86–92	Grams
**10**	Triphosgene	−90–−78		62	Grams

**Table 2:** Advantages and disadvantages of liquid-phase and solid-phase approaches to peptide synthesis.

Liquid-Phase Peptide Synthesis		Solid-Phase Peptide Synthesis	
Advantages	Disadvantages	Advantages	Disadvantages
Relative ease of scale-up; straightforward characterization of intermediate compounds; and the possibility of using protecting groups removable by hydrogenolysis, such as Cbz.	Laborious purification at each step; low yields in the synthesis of long peptide sequences; and prolonged reaction times for certain steps (in many cases, the acylation step is carried out overnight).	Possibility of automation; simple work-up after each reaction; and preparative simplicity with shorter reaction times for each step (according to standard protocols for 2-CTC resin, the acylation step is typically carried out for about 2 h).	The need to cleave intermediates from the resin for characterization; difficulties associated with scale-up; and the frequent use of more expensive reagents.

## Data Availability

No new data were created or analyzed in this study. Data sharing is not applicable to this article.

## Abbreviations

The following abbreviations are used in this manuscript:

ACN	Acetonitrile
Ar	Aryl
Boc	*tert*-Butoxycaronyl
Bpin	Pinacolborane group
^t^Bu	*tert*-Butyl
Cbz, Z	Benzyloxycarbonyl
CDI	Carbonyldiimidazole
DBU	1,8-Diazabicyclo[5.4.0]undec-7-ene
DCC	N,N′-Dicyclohexylcarbodiimide
DCE	Dichloroethane
DCL	N-[N-[(*S*)-1,3-Dicarboxypropyl]carbamoyl]-(*S*)-*L*-lysine
DCM	Dichloromethane
Dde	1-(4,4-dimethyl-2,6-dioxocyclohex-1-ylidene)ethyl
DIC	N,N’-Diisopropylcarbodiimide
DIPEA	N,N-Diisopropylethylamine
DMAc	N,N-Dimethylacetamide
DMAP	4-(Dimethylamino)pyridine
DMF	Dimethylformamide
DSC	N,N′-Disuccinimidyl carbonate
DSS	Disuccinimidyl suberate
Fmoc	Fluorenylmethyloxycarbonyl group
GRPr	Gastrin-releasing peptide receptor
HATU	1-[Bis(dimethylamino)methylene]-1*H*-1,2,3-triazolo[4,5-*b*]pyridinium 3-oxid hexafluorophosphate
HBTU	N,N,N′,N′-Tetramethyl-*O*-(1*H*-benzotriazol-1-yl)uronium hexafluorophosphate
HOAt	1-Hydroxy-7-azabenzotriazole
HOBt	1-Hydroxybenzotriazole
Me	Methyl
MeOTf	Methyl trifluoromethanesulfonate
NAAG	N-Acetylaspartylglutamate
NHS	N-Hyrdoxysuccinimid
PCa	Prostate cancer
PDLLA	poly(*d*,*l*-lactide)
PEG	Polyethylene glycol
PET/CT	Positron emission tomography/computed tomography
PFBME	Perfluorobutyl methyl ether
Pfp	Pentafluorophenyl
PG	Protective group
PLGA	poly(lactide-*co*-glycolide)
PMB	*para*-Methoxybenzyl
PSMA	Prostate-specific membrane antigen
PyBOP	(Benzotriazol-1-yloxy)tripyrrolidinophosphonium hexafluorophosphate
rt	Room temperature
STAB	Sodium triacetoxyborohydride
TBTU	N,N,N′,N′-Tetramethyl-*O*-(1*H*-benzotriazol-1-yl)uronium tetrafluoroborate
TEABC	Tetraethylammonium bicarbonate
TFA	Trifluoroacetic acid
TFP	2,3,5,6-Tetrafluorophenyl
THF	Tetrahydrofuran
TIBS	Tri-*iso*-butylsilane
TIPS	Tri-*iso*-propylsilane
TMS	Trimethylsilyl
Trt	Trityl
TsOH	*para*-Toluenesulfonic acid
TSTU	N,N,N′,N′-tetramethyl-*O*-(*N*-succinimidyl)uronium tetrafluoroborate
ZBG	Zinc-binding group
